# The global, regional, and national burden of oesophageal cancer and its attributable risk factors in 195 countries and territories, 1990–2017: a systematic analysis for the Global Burden of Disease Study 2017

**DOI:** 10.1016/S2468-1253(20)30007-8

**Published:** 2020-04-01

**Authors:** GBD 2017 Oesophageal Cancer Collaborators, Farin Kamangar, Dariush Nasrollahzadeh, Saeid Safiri, Sadaf G Sepanlou, Christina Fitzmaurice, Kevin S Ikuta, Catherine Bisignano, Farhad Islami, Gholamreza Roshandel, Stephen S Lim, Hassan Abolhassani, Eman Abu-Gharbieh, Rufus Adesoji Adedoyin, Shailesh M Advani, Muktar Beshir Ahmed, Miloud Taki Eddine Aichour, Tomi Akinyemiju, Chisom Joyqueenet Akunna, Fares Alahdab, Vahid Alipour, Amir Almasi-Hashiani, Abdulaziz M Almulhim, Nahla Hamed Anber, Alireza Ansari-Moghaddam, Jalal Arabloo, Morteza Arab-Zozani, Atalel Fentahun Awedew, Alaa Badawi, Kathleen S Sachiko Berfield, Kidanemaryam Berhe, Krittika Bhattacharyya, Antonio Biondi, Tone Bjørge, Antonio Maria Borzì, Cristina Bosetti, Giulia Carreras, Felix Carvalho, Clara Castro, Dinh-Toi Chu, Vera Marisa Costa, Baye Dagnew, Jiregna Darega Gela, Ahmad Daryani, Feleke Mekonnen Demeke, Gebre Teklemariam Demoz, Mostafa Dianatinasab, Iffat Elbarazi, Mohammad Hassan Emamian, Arash Etemadi, Pawan Sirwan Faris, Eduarda Fernandes, Irina Filip, Florian Fischer, Mohamed M Gad, Silvano Gallus, Abadi Kahsu Gebre, Tsegaye Tewelde Gebrehiwot, Gebreamlak Gebremedhn Gebremeskel, Begashaw Melaku Gebresillassie, Fatemeh Ghasemi-kebria, Ahmad Ghashghaee, Nermin Ghith, Mahaveer Golechha, Giuseppe Gorini, Rahul Gupta, Nima Hafezi-Nejad, Arvin Haj-Mirzaian, James D. Harvey, Maryam Hashemian, Hamid Yimam Hassen, Simon I Hay, Andualem Henok, Chi Linh Hoang, H Dean Hosgood, Mowafa Househ, Olayinka Stephen Ilesanmi, Milena D. Ilic, Seyed Sina Naghibi Irvani, Charvi Jain, Spencer L James, Sun Ha Jee, Ravi Prakash Jha, Farahnaz Joukar, Ali Kabir, Amir Kasaeian, Mesfin Wudu Kassaw, Supreet Kaur, Andre Pascal Kengne, Esma Kerboua, Yousef Saleh Khader, Rovshan Khalilov, Ejaz Ahmad Khan, Abdullah T Khoja, Jonathan M Kocarnik, Hamidreza Komaki, Vivek Kumar, Carlo La Vecchia, Savita Lasrado, Bingyu Li, Alan D Lopez, Azeem Majeed, Navid Manafi, Ana Laura Manda, Fariborz Mansour-Ghanaei, Manu Raj Mathur, Varshil Mehta, Dhruv Mehta, Walter Mendoza, Prasanna Mithra, Karzan Abdulmuhsin Mohammad, Abdollah Mohammadian-Hafshejani, Reza Mohammadpourhodki, Jemal Abdu Mohammed, Farnam Mohebi, Ali H Mokdad, Lorenzo Monasta, Delaram Moosavi, Mahmood Moosazadeh, Ghobad Moradi, Farhad Moradpour, Rahmatollah Moradzadeh, Gurudatta Naik, Ionut Negoi, Haruna Asura Nggada, Huong Lan Thi Nguyen, Rajan Nikbakhsh, Molly R Nixon, Andrew T Olagunju, Tinuke O Olagunju, Jagadish Rao. Padubidri, Keyvan Pakshir, Shanti Patel, Mona Pathak, Hai Quang Pham, Akram Pourshams, Navid Rabiee, Mohammad Rabiee, Amir Radfar, Alireza Rafiei, Kiana Ramezanzadeh, Goura Kishor Rath, Priya Rathi, Salman Rawaf, David Laith Rawaf, Nima Rezaei, Elias Merdassa Roro, Anas M Saad, Hamideh Salimzadeh, Abdallah M Samy, Benn Sartorius, Arash Sarveazad, Mario Sekerija, Feng Sha, Morteza Shamsizadeh, Sara Sheikhbahaei, Reza Shirkoohi, Sudeep K Siddappa Malleshappa, Jasvinder A Singh, Dhirendra Narain Sinha, Catalin-Gabriel Smarandache, Sergey Soshnikov, Hafiz Ansar Rasul Suleria, Degena Bahrey Tadesse, Berhe Etsay Tesfay, Bhaskar Thakur, Eugenio Traini, Khanh Bao Tran, Bach Xuan Tran, Irfan Ullah, Marco Vacante, Yousef Veisani, Isidora S Vujcic, Girmay Teklay Weldesamuel, Rixing Xu, Vahid Yazdi-Feyzabadi, Deniz Yuce, Vesna Zadnik, Zoubida Zaidi, Zhi-Jiang Zhang, Reza Malekzadeh, Mohsen Naghavi

## Abstract

**Background:**

Oesophageal cancer is a common and often fatal cancer that has two main histological subtypes: oesophageal squamous cell carcinoma and oesophageal adenocarcinoma. Updated statistics on the incidence and mortality of oesophageal cancer, and on the disability-adjusted life-years (DALYs) caused by the disease, can assist policy makers in allocating resources for prevention, treatment, and care of oesophageal cancer. We report the latest estimates of these statistics for 195 countries and territories between 1990 and 2017, by age, sex, and Socio-demographic Index (SDI), using data from the Global Burden of Diseases, Injuries, and Risk Factors Study 2017 (GBD).

**Methods:**

We used data from vital registration systems, vital registration-samples, verbal autopsy records, and cancer registries, combined with relevant modelling, to estimate the mortality, incidence, and burden of oesophageal cancer from 1990 to 2017. Mortality-to-incidence ratios (MIRs) were estimated and fed into a Cause of Death Ensemble model (CODEm) including risk factors. MIRs were used for mortality and non-fatal modelling. Estimates of DALYs attributable to the main risk factors of oesophageal cancer available in GBD were also calculated. The proportion of oesophageal squamous cell carcinoma to all oesophageal cancers was extracted by use of publicly available data, and its variation was examined against SDI, the Healthcare Access and Quality (HAQ) Index, and available risk factors in GBD that are specific for oesophageal squamous cell carcinoma (eg, unimproved water source and indoor air pollution) and for oesophageal adenocarcinoma (gastro-oesophageal reflux disease).

**Findings:**

There were 473 000 (95% uncertainty interval [95% UI] 459 000–485 000) new cases of oesophageal cancer and 436 000 (425 000–448 000) deaths due to oesophageal cancer in 2017. Age-standardised incidence was 5·9 (5·7–6·1) per 100 000 population and age-standardised mortality was 5·5 (5·3–5·6) per 100 000. Oesophageal cancer caused 9·78 million (9·53–10·03) DALYs, with an age-standardised rate of 120 (117–123) per 100 000 population. Between 1990 and 2017, age-standardised incidence decreased by 22·0% (18·6–25·2), mortality decreased by 29·0% (25·8–32·0), and DALYs decreased by 33·4% (30·4–36·1) globally. However, as a result of population growth and ageing, the total number of new cases increased by 52·3% (45·9–58·9), from 310 000 (300 000–322 000) to 473 000 (459 000–485 000); the number of deaths increased by 40·0% (34·1–46·3), from 311 000 (301 000–323 000) to 436 000 (425 000–448 000); and total DALYs increased by 27·4% (22·1–33·1), from 7·68 million (7·42–7·97) to 9·78 million (9·53–10·03). At the national level, China had the highest number of incident cases (235 000 [223 000–246 000]), deaths (213 000 [203 000–223 000]), and DALYs (4·46 million [4·25–4·69]) in 2017. The highest national-level age-standardised incidence rates in 2017 were observed in Malawi (23·0 [19·4–26·5] per 100 000 population) and Mongolia (18·5 [16·4–20·8] per 100 000). In 2017, age-standardised incidence was 2·7 times higher, mortality 2·9 times higher, and DALYs 3·0 times higher in males than in females. In 2017, a substantial proportion of oesophageal cancer DALYs were attributable to known risk factors: tobacco smoking (39·0% [35·5–42·2]), alcohol consumption (33·8% [27·3–39·9]), high BMI (19·5% [6·3–36·0]), a diet low in fruits (19·1% [4·2–34·6]), and use of chewing tobacco (7·5% [5·2–9·6]). Countries with a low SDI and HAQ Index and high levels of indoor air pollution had a higher proportion of oesophageal squamous cell carcinoma to all oesophageal cancer cases than did countries with a high SDI and HAQ Index and with low levels of indoor air pollution.

**Interpretation:**

Despite reductions in age-standardised incidence and mortality rates, oesophageal cancer remains a major cause of cancer mortality and burden across the world. Oesophageal cancer is a highly fatal disease, requiring increased primary prevention efforts and, possibly, screening in some high-risk areas. Substantial variation exists in age-standardised incidence rates across regions and countries, for reasons that are unclear.

**Funding:**

Bill & Melinda Gates Foundation.

**Research in context**

**Evidence before this study**

Oesophageal cancer is one of the leading causes of cancer mortality worldwide. Previous publications have reported on various health metrics of oesophageal cancer, but to our knowledge, no study has provided detailed estimates of incidence, mortality, disability-adjusted life-years (DALYs), and DALYs attributable to major risk factors for 195 countries and territories around the world. Furthermore, previous studies have not investigated the country-level correlation between the proportion of new oesophageal squamous cell carcinoma to all oesophageal cancer cases and potential risk factors for oesophageal squamous cell carcinoma, such as indoor air pollution and inadequate access to improved water sources.

**Added value of this study**

We used data from the Global Burden of Diseases, Injuries, and Risk Factors Study (GBD) 2017 to provide the most up-to-date estimates on a wide range of health measures related to oesophageal cancer at global, regional, and country-specific levels for 195 countries and territories, and by sex, age group, and Socio-demographic Index (SDI). We present estimated numbers and age-standardised incidence rates for oesophageal cancer in 2017, as well as trends from 1990 to 2017. We also describe DALYs attributed to several major risk factors for oesophageal cancer. Furthermore, for the first time, we present the correlation of the country-level proportion of new oesophageal squamous cell carcinoma to all oesophageal cancer cases with several potential risk factors for oesophageal squamous cell carcinoma, including indoor air pollution, inadequate access to improved water sources, and SDI. These correlations can guide further investigations into the causes of oesophageal squamous cell carcinoma.

**Implications of all the available evidence**

Oesophageal cancer remains a major cause of cancer mortality and disease burden across the world, requiring substantial preventive measures and possibly screening by use of upper gastrointestinal endoscopy in certain high-risk countries. The highest burden of oesophageal cancer (in terms of the number of DALYs) is in east Asia, particularly China, where screening has been shown to be effective in increasing survival rates. Other high-incidence regions are in central Asia and sub-Saharan Africa. The main reasons for these high rates are unknown. Further research into the causes of oesophageal cancer in these high-risk areas is warranted. Our findings are consistent with previous studies that suggest indoor air pollution, inadequate access to improved water sources, and low socioeconomic status are associated with increased risk of oesophageal squamous cell carcinoma. Several epidemiological studies are ongoing by different research groups, and we expect to see new findings over the next few years.

## Introduction

Oesophageal cancer is one of the leading causes of cancer mortality worldwide.^1^, ^2^, ^3^ Together with other forms of gastrointestinal cancers, such as stomach cancer, colorectal cancer, pancreatic cancer, and hepatocellular carcinoma, oesophageal cancer causes approximately a third of all disability-adjusted life-years (DALYs) due to cancer.^1^, ^3^

Previous publications have reported on various health metrics of oesophageal cancer, including its incidence, mortality, and DALY rates, as well as its risk factors.^3^ Some of these studies have presented comprehensive global data for all cancers combined and more sparse data for each individual cancer.^1^, ^2^, ^3^ Others have provided more detailed data about oesophageal cancer for a certain country or region,^4^, ^5^ or used global data that were several years old and did not have information about DALYs.^6^, ^7^ We provide, to the best of our knowledge, the first set of updated estimates about a wide range of oesophageal cancer health measures at global, regional, and country-specific levels for 195 countries and territories.

There are two main histological subtypes of oesophageal cancer: oesophageal adenocarcinoma (which is linked to obesity, smoking, and gastro-oesophageal reflux disease) and oesophageal squamous cell carcinoma (which is linked to alcohol and tobacco consumption). Although several high-risk regions for oesophageal squamous cell carcinoma are adjacent to regions with medium or low risk, such a pattern has not been observed for oesophageal adenocarcinoma. Globally, more than 85% of all incident oesophageal cancer cases are oesophageal squamous cell carcinoma.^8^

In this analysis of data from the Global Burden of Diseases, Injuries, and Risk Factors Study (GBD) 2017, we present estimates of the number of incident cases and deaths, as well as age-standardised incidence, mortality, and DALY rates for oesophageal cancer from 1990 to 2017, by age and sex, for 195 countries and territories. Furthermore, we provide age-standardised DALYs by Socio-demographic Index (SDI). Finally, we provide estimates of the proportion of DALYs attributable to several major oesophageal cancer risk factors, including tobacco smoking and chewing, alcohol consumption, low intake of fruits, and high body-mass index (BMI). Through this comprehensive evaluation of global oesophageal cancer measures, we hope to provide additional information for policy makers, funding agencies, and researchers, so they can develop strategies to help prevent and treat oesophageal cancer in high-risk locations, determine allocation of scarce health resources, and guide the direction of future research on oesophageal cancer.

## Methods

### Overview

This study is part of GBD 2017, which includes estimates for 195 countries and territories, 21 regions, and seven super-regions. GBD 2017 presented estimates for 359 diseases and injuries; 282 causes of death; and 84 behavioural, environmental, occupational, and metabolic risk factors. The methodology used to collect data for this research has been discussed previously.^9^, ^10^, ^11^, ^12^ Rates per 100 000 population were age-standardised according to the GBD world population.^13^ 95% uncertainty intervals (95% UIs) were reported for estimates, including for sources of uncertainty arising from measurement error, potential biases, and modelling. Statistical analyses were done with Python (version 2.7.14), R (version 3.3.2), and Stata (release 15) software. This study is compliant with the Guidelines for Accurate and Transparent Health Estimates Reporting (GATHER) statement.

### Data and estimation framework

Data came from vital registration systems (19 323 site-years), vital registration-samples (793 site-years), verbal autopsy records (419 site-years), and cancer registries (5497 site-years).^9^ Of these, only cancer registries had both mortality and incidence data, whereas vital registries and verbal autopsies had only mortality data. By use of International Classification of Diseases 10 (ICD-10) codes,^10^ all cancers coded as C15.0-C15.9, D00.1, and D13.0 were considered to be oesophageal cancer. Four sequelae and associated disability weights were used, including diagnosis, controlled, metastatic, and terminal phases (appendix p 9).^10^ Disability weights ranged from 0 (perfect health) to 1 (equivalent to death).

### Mortality modelling

Some locations had high-quality incidence and mortality cancer registry data, whereas data were sparse in other locations.^9^ Therefore, incidence and mortality estimates were obtained with a combination of available data and modelling. We first estimated mortality-to-incidence ratios (MIRs) using sources that had accurate incidence and mortality data for the same year. MIRs were estimated for other locations by use of a linear-step mixed-effects model with logit link functions, with the Healthcare Access and Quality (HAQ) Index, age, and sex as covariates. The resulting estimates were then smoothed over space and time and adjusted with spatiotemporal Gaussian process regression.^14^ Estimated mortality data for each site were obtained by multiplying incidence data by MIRs. As the next step, both observed and estimated mortality data were fed into a Cause of Death Ensemble model (CODEm), along with the mortality data from vital registration and verbal autopsy.^9^ The covariates included in CODEm that were assumed to have a positive relationship with oesophageal cancer mortality were smoking prevalence, tobacco consumption (cigarettes per capita), mean BMI, log-transformed age-standardised summary exposure value scalar for oesophageal cancer, alcohol (L per person), and indoor air pollution (prevalence of households cooking with coal or biomass). The covariates included in the CODEm that were assumed to have a negative relationship with oesophageal cancer were fruit intake, adjusted (g); improved water source (proportion with access); HAQ Index; vegetable intake, adjusted (g); sanitation (proportion with access); and education (years per person). Finally, the covariates included in the CODEm that had no prior assumptions about the direction of the relationship were lag-distributed income (international dollar [Int$] per person) and SDI. All the covariates had a plausible association with oesophageal cancer deaths based on the published literature, although evidence of causality was not required for their inclusion in the model.^12^, ^15^

### Non-fatal modelling

The final mortality estimates were obtained from CoDCorrect,^9^ with modelling described above, and were divided by the MIR to compute oesophageal cancer incidence. The prevalence of oesophageal cancer was calculated by modelling survival for each incidence cohort by using the MIR as a scalar and adjusting for expected background mortality.^10^ The cohort members who had survived more than 10 years were assumed to be cured, and these cases were divided into two sequelae: the diagnosis and primary therapy phase and the controlled phase. Furthermore, the prevalence for the cohort that died during the 10-year period was categorised into four sequelae (appendix p 9). A time duration of 5 months was used to define the diagnosis and primary therapy phase,^16^ 4·6 months used to define the metastatic phase,^17^ and 1 month used to define the terminal phase. The remaining time was assigned to the controlled phase.

Following this process, each sequela-specific prevalence rate was multiplied by a sequela-specific disability weight to estimate the sequelae-specific years lived with disability (YLDs). DALYs were calculated as the sum years of life lost (YLLs) due to premature death and YLDs.

### Socio-demographic Index (SDI)

SDI was used in this study to determine the relationship of a country's socioeconomic development status with age-standardised incidence and mortality rates for oesophageal cancer. In GBD 2017, SDI was revised to better reflect the development status of each country.^9^, ^10^, ^11^ SDI ranges from 0 (worst) to 1 (best) and is composed of the total fertility rate under the age of 25 years, mean education for those aged 15 years and older, and lag-distributed income per capita.^13^ The HAQ Index determines the personal access to and quality of health care for 195 countries and territories and is calculated on the basis of amenable mortality, or deaths from causes that should not occur in the presence of effective medical care. The HAQ Index ranges from 0 (worst) to 100 (best). More details about the HAQ Index are presented in previous publications.^18^

### Risk factors

We calculated and reported the percentage of DALYs due to oesophageal cancer that were attributable to smoking, alcohol use, high BMI, a diet low in fruits, and chewing tobacco (established oesophageal cancer risk factors with available data in GBD). Details about the definitions of these risk factors and their relative risk for oesophageal cancer are described elsewhere.^12^

Publicly available data for the two main histological subtypes of oesophageal cancer (ie, oesophageal squamous cell carcinoma and oesophageal adenocarcinoma) were obtained from Cancer Incidence in Five Continents (Vol XI).^7^ Local cancer registries from 151 countries provided data about the incidence of oesophageal squamous cell carcinoma, oesophageal adenocarcinoma, and all oesophageal cancer. The proportion of the incidence of oesophageal squamous cell carcinoma out of all oesophageal cancer cases was calculated for each country and territory and linked to GBD estimates using country codes. We regressed the proportion of oesophageal squamous cell carcinoma to all oesophageal cancer cases (as a continuous response variable) against SDI and the HAQ Index, and against risk factors for oesophageal squamous cell carcinoma (percentage of the population with access to improved water sources, and prevalence of households cooking with coal or biomass [indoor air pollution]) and oesophageal adenocarcinoma (gastro-oesophageal reflux disease).

### Role of the funding source

The funder of the study had no role in study design, data collection, data analysis, data interpretation, or the writing of the report. All authors had full access to the data in the study and had final responsibility for the decision to submit for publication.

## Results

In 2017, there were 473 000 (95% UI 459 000–485 000) new cases of oesophageal cancer and 436 000 (425 000–448 000) deaths due to oesophageal cancer. Age-standardised incidence was 5·9 (5·7–6·1) per 100 000 population and the age-standardised mortality rate was 5·5 (5·3–5·6) per 100 000 population. 9·78 million (9·53–10·03) DALYs were due to oesophageal cancer, with an age-standardised rate of 119·9 (116·9–123·0) per 100 000 population (appendix pp 10–33).

Between 1990 and 2017, global age-standardised incidence decreased by 22·0% (95% UI 18·6–25·2), mortality decreased by 29·0% (25·8–32·0), and DALYs decreased by 33·4% (30·4–36·1; appendix pp 10–33). However, during the same period, the total number of new cases increased by 52·3% (45·9–58·9), from 310 000 (301 000–322 000) to 473 000 (459 000–485 000); the number of deaths increased by 40·0% (34·1–46·3), from 311 000 (301 000–323 000) to 436 000 (425 000–448 000); and total DALYs increased by 27·4% (22·1–33·1), from 7·68 million (7·42–7·97) to 9·78 million (9·53–10·03; appendix pp 2, 10–33).

**Figure 1:**
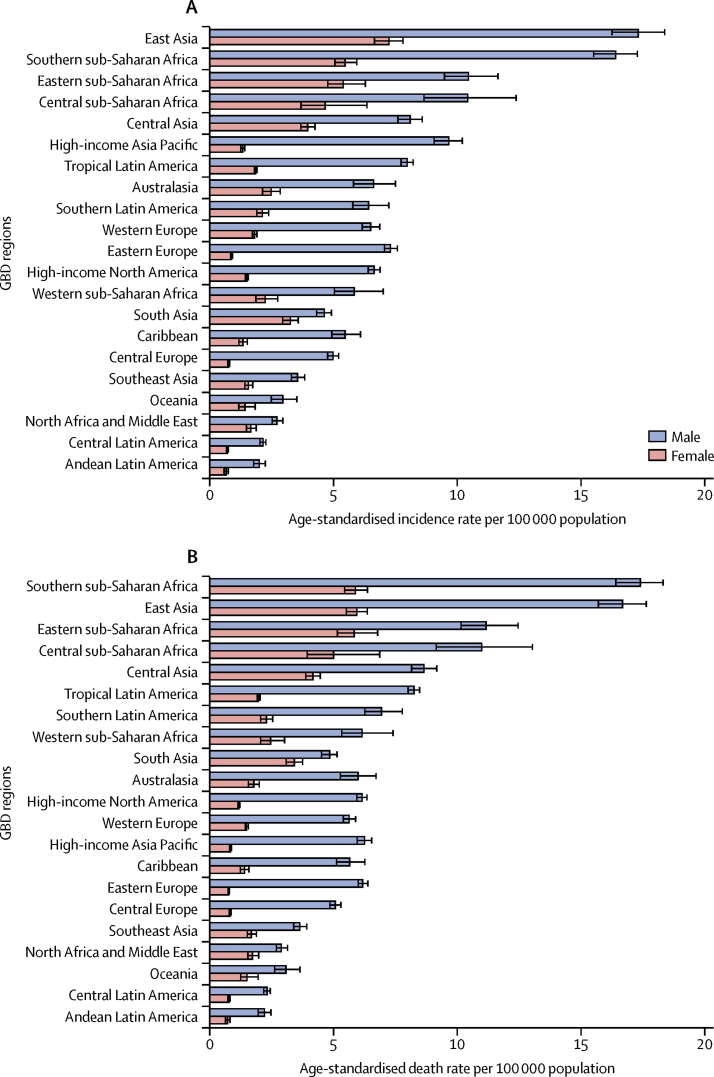
The age-standardised incidence (A) and death rates (B) of oesophageal cancer in 2017 for 21 GBD regions, by sex GBD=Global Burden of Diseases, Injuries, and Risk Factors Study.

From among 21 GBD regions, in 2017, the highest age-standardised incidence rates were in east Asia (12·1 [95% UI 11·5–12·7] per 100 000 population), southern sub-Saharan Africa (10·0 [9·5–10·4] per 100 000 population), eastern sub-Saharan Africa (7·8 [7·2–8·6] per 100 000 population), central sub-Saharan Africa (7·3 [6·2–8·5] per 100 000 population), and central Asia (5·7 [5·4–5·9]; figure 1A, appendix pp 10–17). In 2017, the highest regional age-standardised incidence rate (12·1 per 100 000 population in east Asia) was 9·3 times higher than the lowest (1·3 [1·2–1·4] per 100 000 population in Andean Latin America; appendix pp 10–17). With some minor change in rank order, regions with high age-standardised incidence rates also had the highest age-standardised mortality and DALY rates (figure 1B, appendix pp 18–33). Because of the high incidence rates and its large population, over half of all new cases of oesophageal cancer in 2017 came from east Asia (245 000 [233 000–256 000] of 473 000 [459 000–485 000] global incident cases; 51·8%; appendix pp 10–17). By contrast, the lowest age-standardised incidence, mortality, and DALY rates were seen in Andean Latin America, central Latin America, Oceania, north Africa and the Middle East, and southeast Asia (figure 1, appendix pp 10–33).

Stark differences were observed in age-standardised incidence rates of oesophageal cancer between nearby regions. For example, the age-standardised incidence rate in southeast Asia, which is close to east Asia, was low (2·5 [95% UI 2·4–2·7] per 100 000 population in 2017). Similarly, in contrast to the high age-standardised incidence rate seen in east sub-Saharan Africa (7·8 [7·2–8·6] per 100 000 population in 2017), the age-adjusted incidence rate in western sub-Saharan Africa was low (4·0 [3·5–4·7] per 100 000 population in 2017).

Between 1990 and 2017, the regional age-standardised incidence, mortality, and DALY rates for oesophageal cancer decreased in all GBD regions except for high-income North America for age-standardised incidence rates (7·4% [95% UI 3·9–11·1]), and western sub-Saharan Africa for all three measures (percentage change 35·7% [15·6–58·9] for age-standardised incidence, 37·2% [17·1–60·3] for age-standardised mortality, and 31·4% [12·3–55·4] for age-standardised DALYs; appendix pp 3–4, 10–33). The sharpest declines in age-standardised incidence rates over the study period were seen in central Asia (55·1% [95% UI 52·5–57·5]), east Asia (36·1% [31·3–40·9]), southern Latin America (40·9% [34·8–45·7]), Andean Latin America (33·5% [26·5–40·4]), central Latin America (32·8% [29·6–36·0]), central sub-Saharan Africa (30·1% [18·5–41·2]), and eastern sub-Saharan Africa (27·4% [19·0–34·7]).

**Figure 2:**
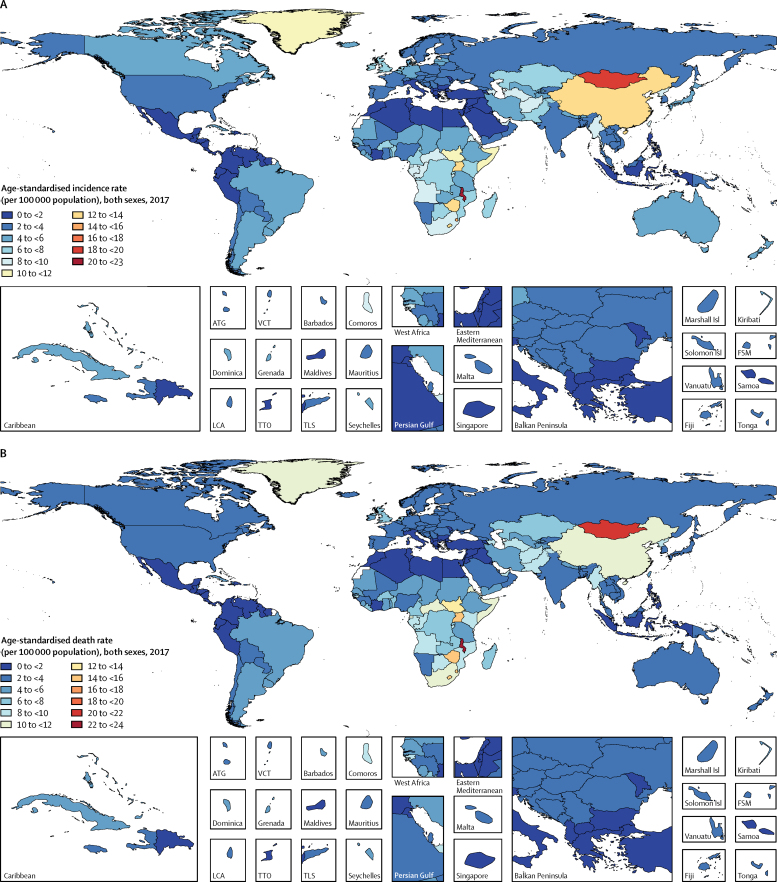
Age-standardised incidence (A) and death rates (B) of oesophageal cancer per 100 000 population in 2017, by country and territory ATG=Antigua and Barbuda. VCT=Saint Vincent and the Grenadines. LCA=Saint Lucia. TTO=Trinidad and Tobago. Isl=Islands. FSM=Federated States of Micronesia. TLS=Timor-Leste.

At the national level, China had the highest number of incident cases (235 000 [95% UI 223 000–246 000]), deaths (213 000 [203 000–223 000]), and DALYs (4·46 million [4·25–4·69]) in 2017, comprising nearly half of new cases, deaths, and DALYs globally that year. The highest estimated national-level age-standardised incidence, mortality, and DALY rates in 2017 were observed in certain countries in sub-Saharan Africa (eg, Malawi, eSwatini, Lesotho, Zimbabwe, and Uganda), central Asia (eg, Mongolia, Turkmenistan, and Azerbaijan), and east Asia (eg, China; figure 2; appendix pp 10–33). Malawi had the highest age-standardised incidence rate (23·0 [19·4–26·5] per 100 000 population), followed by Mongolia (18·5 (16·4–20·8) per 100 000 population). In 2017, the ratio of the highest age-standardised incidence rate to the lowest (0·6 [0·6–0·7] per 100 000 population in Iraq) was 1. Patterns of age-standardised mortality and DALY rates closely followed those of age-standardised incidence rates, which reflects the poor survival of patients with oesophageal cancer, particularly in low-income and middle-income countries.

**Figure 3:**
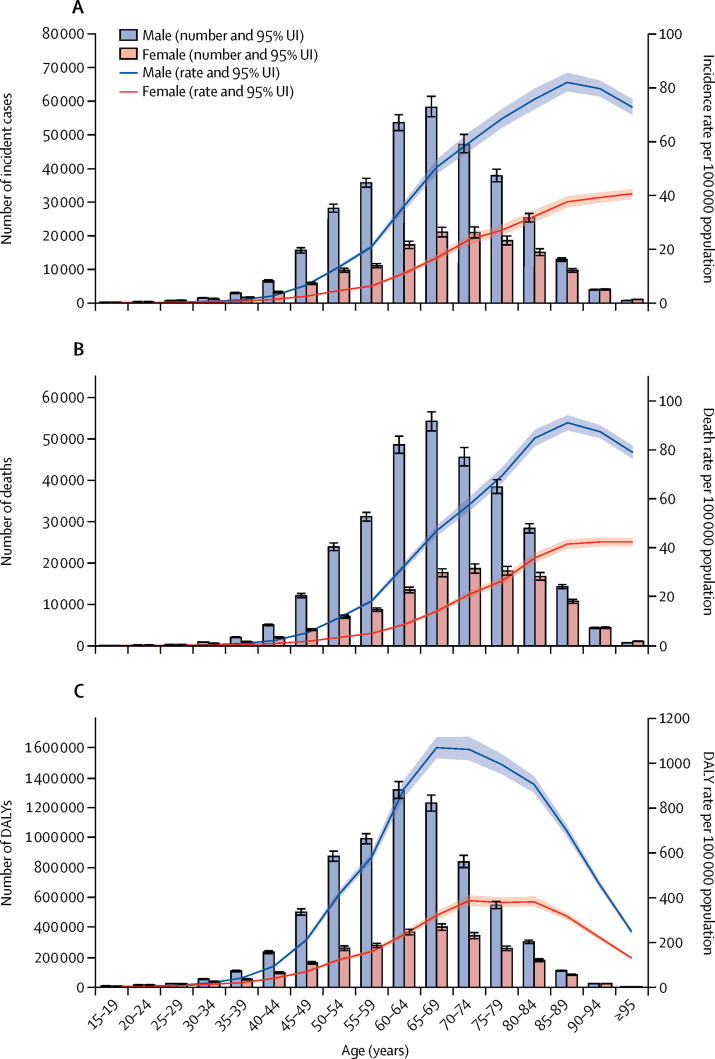
Global number and age-standardised rates of incidence (A), mortality (B), and DALYs (C) of oesophageal cancer per 100 000 population by age and sex, 2017 Shading indicates the upper and lower limits of the 95% uncertainty intervals (95% UIs). DALY=disability-adjusted life-year.

**Figure 4:**
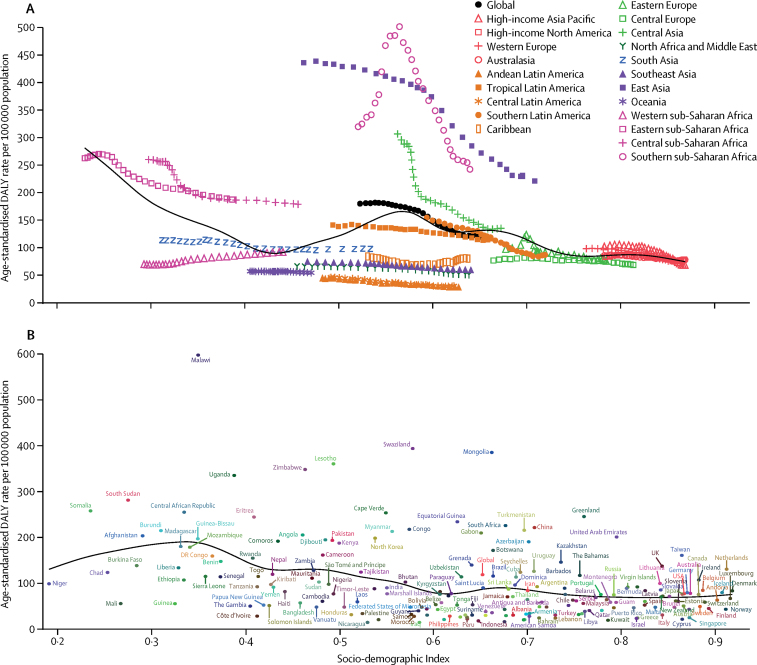
Age-standardised DALY rates for oesophageal cancer for 21 GBD regions (A) and 195 countries and territories (B) by Socio-demographic Index, 1990–2017 Expected values based on Socio-demographic Index and disease rates in all locations are shown as the black line. DALY=disability-adjusted life-year. GBD=Global Burden of Diseases, Injuries, and Risk Factors Study.

Similar to the global and regional trends, between 1990 and 2017, most countries and territories had a decrease in age-standardised incidence, mortality, and DALY rates due to oesophageal cancer (appendix pp 10–33). For example, age-standardised incidence rates decreased in Turkmenistan (by 71·9% [95% UI 69·1 to 74·6]), Uzbekistan (by 68·1% [64·1 to 71·7]), Kazakhstan (by 64·3% [61·4 to 67·2]), Kyrgyzstan (by 55·4% [51·1 to 59·3]), and China (by 36·9% [32·1 to 41·7]). However, there were clear exceptions to the overall trend, particularly among high-income countries. For example, age-standardised incidence rates increased in the USA (6·7% [95% UI 3·1 to 10·4]), Canada (10·6% [–0·4 to 23·5]), Austria (23·1% [9·9 to 38·4]), Germany (38·4% [19·9 to 60·2]), Latvia (53·3% [28·0 to 81·4]), and the Netherlands (86·6% [68·2 to 106·6]). Likewise, some countries in western sub-Saharan Africa, such as Sierra Leone (61·4% [23·9 to 109·9]), Benin (74·6% [38·9 to 124·2]), and Chad (84·1% [46·4 to 128·9]), had increased age-standardised incidence rates. Incidence, mortality, and DALY rates varied by age, sex, and SDI in 2017. Incidence and mortality rates started climbing at age 50 years but DALYs started climbing at age 40 years and dropped in the oldest age groups, as younger age groups lost more years of life (figure 3). Incidence, mortality, and DALY rates were also higher in males than in females in all age groups (figure 3). Age-standardised incidence rates were 2·7 times higher, mortality rates 2·9 times higher, and DALY rates 3·0 times higher in males than in females (appendix pp 10–33). Because oesophageal cancer is a highly fatal disease with a high MIR (0·91), almost all DALYs (98·7%) were attributable to YLLs due to premature death (appendix p 5). The observed regional and national age-standardised DALY rates in relation to SDI, versus the expected level for each location on the basis of SDI, are shown in figure 4. The high-income regions, central and eastern Europe, and tropical and southern Latin America closely followed expected trends over the study period. Among many of the middle SDI regions, however, the observed patterns varied widely, with some regions staying well below expected levels throughout the study period with little change in age-standardised rates and others well above expected levels but with fluctuating or decreasing age-standardised rates (figure 4A). In 2017, there was an inverse association between age-standardised DALY rates for oesophageal cancer and SDI at the national level, with some exceptions (figure 4B). Similar patterns were observed for incidence and mortality in relationship to SDI (appendix p 6).

**Figure 5:**
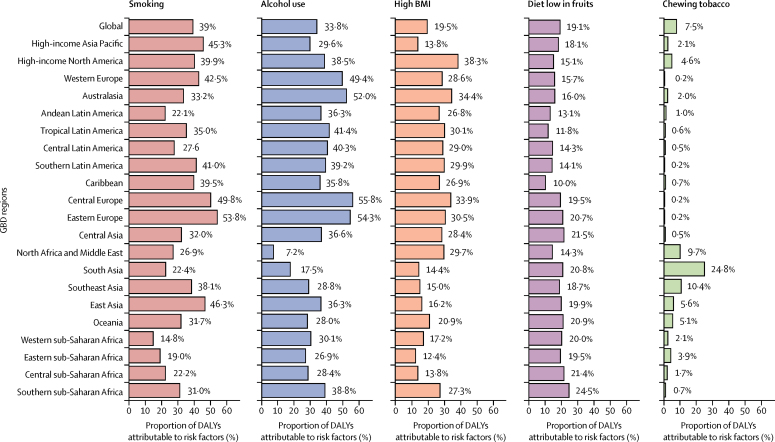
Proportion of oesophageal cancer DALYs attributable to tobacco smoking and chewing, alcohol use, high BMI, and low intake of fruits, for 21 GBD regions, 2017 BMI=body-mass index. DALY=disability-adjusted life-year. GBD=Global Burden of Diseases, Injuries, and Risk Factors Study.

At the global level, a substantial proportion of DALYs were attributable to the five risk factors for which GBD estimates were available, including 39·0% (95% UI 35·5–42·2) attributable to tobacco smoking, 33·8% (27·3–39·9) to alcohol consumption, 19·5% (6·3–36·0) to high BMI, 19·1% (4·2–34·6) to a diet low in fruits, and 7·5% (5·2–9·6) to use of chewing tobacco (figure 5). The impact of these risk factors varied by region. For example, the impact of smoking was highest in eastern Europe (53·7% of DALYs were attributable to smoking) and central Europe (49·8%), where smoking is still prevalent, and lowest in western sub-Saharan Africa (14·8%). Likewise, the impact of alcohol consumption was highest in central Europe (55·8% of DALYs attributable to alcohol) and eastern Europe (54·3%), and lowest in north Africa and the Middle East (7·2%), where alcohol consumption is relatively low.

Clear patterns emerged by generating graphs of the proportion of oesophageal squamous cell carcinoma to oesophageal adenocarcinoma cases and to all oesophageal cancer cases versus SDI, HAQ Index, and household air pollution, which were obtained by linking to data from Cancer Incidence in Five Continents (Vol XI; appendix pp 7, 34–37). At the country level, this proportion was strongly positively correlated with indoor air pollution, even after adjustment for SDI, meaning that countries and territories with worse indoor air pollution had higher proportions of oesophageal squamous cell carcinoma from among all oesophageal cancer cases. The proportion of oesophageal squamous cell carcinoma to all oesophageal cancer cases was also positively correlated with inadequate access to improved water sources, but the strength of this correlation decreased after adjusting for SDI. A higher proportion of oesophageal squamous cell carcinoma to all oesophageal cancer cases was inversely correlated with SDI and the HAQ Index, meaning that countries and territories higher on the SDI and HAQ Indexes had lower proportions of oesophageal squamous cell carcinoma from among all oesophageal cancer cases. No clear pattern was observed for the association between the proportion of oesophageal squamous cell carcinoma to all oesophageal cancer cases versus the prevalence of gastro-oesophageal reflux disease (appendix p 8).

## Discussion

The results of GBD 2017 show that the large variations in age-standardised incidence, mortality, and DALY rates across countries and territories are still a main epidemiological feature of oesophageal cancer (with the majority of cases being oesophageal squamous cell carcinoma), indicating the importance of finer delineation of hotspots to specify at-risk populations. GBD estimates show a decrease in age-adjusted incidence rates in most parts of the world, particularly in regions where oesophageal squamous cell carcinoma is the dominant histological subtype (eg, in central Asia), but an increase in these rates in regions where oesophageal adenocarcinoma is the major subtype (eg, high-income North America), emphasising the importance of collecting data to differentiate these two subtypes. Further investigation is needed of the causes of the high age-standardised incidence rates seen in some parts of the world, particularly in high-risk areas of sub-Saharan Africa, Mongolia, China, and Iran. The observed global decrease in age-standardised incidence rate from 1990 to 2017 is perhaps due to improvements in several social and environmental factors, such as the SDI.

Our findings add to the literature by providing the most up-to-date data on a wide range of oesophageal cancer health metrics, including age-standardised rates of DALYs for 195 countries and territories, DALYs attributable to several major risk factors of oesophageal cancer, and trends of these measures from 1990 to 2017. Although age-standardised incidence and mortality rates of oesophageal cancer have decreased at the global level over the past three decades, absolute numbers of new oesophageal cancer cases and deaths and DALYs attributable to oesophageal cancer have increased as a result of population growth and ageing. These trends over time indicate that oesophageal cancer still causes a substantial disease burden across the world, and the overall burden could continue to rise. Our results corroborate findings from previous studies but use different sources of data and estimation methods. The country-level positive correlations between the proportion of oesophageal squamous cell carcinoma to all oesophageal cancer cases and high indoor pollution, low access to improved water sources, low SDI, and low HAQ Index are novel and support the hypothesis that these factors could contribute to the risk of oesophageal squamous cell carcinoma. Gastro-oesophageal reflux disease, an established risk factor for oesophageal adenocarcinoma (but not oesophageal squamous cell carcinoma), showed a U-shaped relationship with the proportion of oesophageal squamous cell carcinoma to all oesophageal cancer cases. No clear explanation exists for this unexpected finding, although we can suggest some hypotheses. This U-shaped relationship was primarily due to aberrations seen in eastern Europe, an area that is close to geographical areas of Asia where oesophageal squamous cell carcinoma is prevalent, and adjacent to western Europe, where gastro-oesophageal reflux disease and oesophageal adenocarcinoma are highly prevalent. This geographical proximity, combined with spatial modelling, might have affected the estimates. Another possibility is a potential association between gastro-oesophageal reflux disease prevalence and one or more oesophageal squamous cell carcinoma risk factors in these regions.

The estimated numbers of new cases of oesophageal cancer based on GBD estimates were lower than those estimated with the 2018 Global Cancer Observatory (GLOBOCAN).^2^ We estimated 473 000 new cases and 436 000 deaths in 2017 using GBD, versus 572 000 new cases and 509 000 deaths in GLOBOCAN data. These discrepancies are due to incomplete data in both datasets and differences in modelling. For example, weighting the data by the level of their completeness in GBD might give more weight to data from high-income countries, where data are more complete and oesophageal cancer incidence rates are low.

Some countries in east and central Asia, as well as in eastern, central, and southern sub-Saharan Africa, had high age-standardised incidence, mortality, and DALY rates over the study period. These findings are consistent with previous literature. Since at least the early 1970s, vast areas of Asia, extending from China and Mongolia to the Caspian Sea, have been known to have high rates of oesophageal cancer.^19^ This area, dubbed the so-called Asian oesophageal cancer belt,^19^, ^20^, ^21^ was along the path of the Silk Road, which has led to hypotheses that some shared environmental risk or genes passed along this route were the common causes of the observed high risks.^19^, ^21^, ^22^ More than 90% of oesophageal cancer cases in this part of the world are of the squamous cell type (oesophageal squamous cell carcinoma).^22^, ^23^, ^24^ However, tobacco and alcohol consumption, which are the major risk factors for oesophageal squamous cell carcinoma in many parts of the world, are not strong risk factors in the Asian oesophageal cancer belt.^22^, ^23^, ^25^, ^26^ Case-control and cohort studies have found that opium use, consumption of hot tea, low intake of fresh fruits and vegetables, indoor air pollution, exposure to polycyclic aromatic hydrocarbons, lack of access to piped water, poor oral health, and low socioeconomic status are also associated with a higher risk of oesophageal squamous cell carcinoma.^26^, ^27^, ^28^, ^29^, ^30^, ^31^, ^32^ However, as yet, no single dominant risk factor has been identified for oesophageal squamous cell carcinoma in the Asian oesophageal cancer belt. Most published findings of risk factors for oesophageal cancer in the Asian oesophageal cancer belt come from studies done in China and Iran. No recent studies have been done in Mongolia to address the high incidence and mortality rates in this population, although several risk factors are shared with other high-risk areas, such as high level of fluoride in drinking water or drinking hot milk tea.^33^ High rates of oesophageal cancer in eastern, central, and southern sub-Saharan Africa, an area dubbed the African oesophageal cancer corridor, are also well established.^34^ The majority of cases in this area are also oesophageal squamous cell carcinoma. Until recently, few studies had been done to identify risk factors for oesophageal cancer in this region. Recent studies, however, have suggested that tobacco use,^35^ alcohol consumption,^35^, ^36^ dental fluorosis,^34^ consumption of hot beverages,^36^ and exposure to biomass smoke^37^, ^38^ are risk factors. Case-control studies have been completed or are ongoing in several countries in this region, including Kenya, South Africa, Malawi, Uganda, Tanzania, and Zambia, and further results are expected to be published within the next few years.

Although GBD estimates show major variations in age-standardised incidence, mortality, and DALYs across regions and countries, there might be finer variations within countries not captured by these data. A prominent epidemiological feature of oesophageal squamous cell carcinoma, which constitutes the majority of oesophageal cancer cases worldwide, is that, for unknown reasons, rates of this disease can change sharply over relatively short distances. Therefore, countries marked as medium risk or low risk might have areas with a very high risk of oesophageal squamous cell carcinoma. For example, although Iran is not marked as a high-risk country for oesophageal cancer, Golestan Province in the northern part of Iran, bordering Turkmenistan, has some of the highest rates of oesophageal squamous cell carcinoma in the world.^21^, ^39^ Likewise, although India and Brazil are not considered to be high-risk countries, certain areas of these countries (eg, the Kashmir Valley in India and Rio Grande do Sul in Brazil) have high rates of oesophageal cancer.^20^, ^40^, ^41^ Such stark differences in incidence over short distances could lead to erroneous results when spatiotemporal smoothing methods are used. A country with no data and low incidence rates might be considered a high-incidence country if it has a neighbouring country with high incidence rates. Therefore, more granular geographical data need to be collected.^3^

Although there was a global decrease in age-standardised incidence rates of oesophageal cancer between 1990 and 2017, the age-standardised rates increased in several countries, particularly in high-income countries. This is likely to be because the rates of oesophageal adenocarcinoma (but not oesophageal squamous cell carcinoma) have increased in high-income countries (eg, the USA, Germany, and the Netherlands) since the 1990s.^5^, ^8^ The two main histological subtypes of oesophageal cancer, oesophageal squamous cell carcinoma and oesophageal adenocarcinoma, have very distinct risk factors, incidence trends, and geographical distributions.^22^ For example, although alcohol consumption is a major risk factor for oesophageal squamous cell carcinoma, it is not a risk factor for oesophageal adenocarcinoma.^42^, ^43^ Conversely, although obesity and gastro-oesophageal reflux are major risk factors for oesophageal adenocarcinoma,^44^, ^45^ they are not risk factors for oesophageal squamous cell carcinoma. Thus, although we observed an overall decline in the age-standardised rates of oesophageal cancer, the picture is different for oesophageal squamous cell carcinoma and oesophageal adenocarcinoma. The age-adjusted incidence rates of oesophageal cancer increased in a minority of oesophageal squamous cell carcinoma-dominant countries, particularly in countries in sub-Saharan Africa (eg, Chad, Benin, and Sierra Leone), which could be due to improvements in cancer registry systems in this region.

This study found tobacco smoking, tobacco chewing, alcohol consumption, low intake of fruit, and high BMI to be important oesophageal cancer risk factors, accounting for most of the burden. Of these factors, high BMI is an established risk factor for oesophageal adenocarcinoma but has no proven association with oesophageal squamous cell carcinoma risk. Given the poor survival rate of patients with oesophageal cancer, major efforts to reduce these risk factors should be undertaken, particularly given that many oesophageal cancer risk factors (eg, smoking and obesity) are also important risk factors for other major chronic diseases, such as cardiovascular diseases and other cancers.

Although there were some exceptions, we found an overall inverse association between the age-standardised incidence of oesophageal cancer and SDI at the country level. These findings are consistent with previous epidemiological studies that have shown an inverse association between oesophageal squamous cell carcinoma and socioeconomic status.^22^, ^27^, ^30^, ^46^, ^47^ Despite recent rises in the age-standardised incidence rates of oesophageal adenocarcinoma, oesophageal squamous cell carcinoma still constitutes the majority of global oesophageal cancer cases. Therefore, on a global basis, an inverse association between oesophageal cancer and SDI is expected. It has been argued that substantial decreases in the rate of oesophageal squamous cell carcinoma in some of parts of the world might be due to improved socioeconomic status.^48^ Low SDI is a proxy for multiple correlated and interconnected variables such as unimproved water sources and high indoor air pollution. Indeed, recent studies have suggested that exposure to biomass smoke and indoor air pollution, lack of access to piped water, poor oral health, opium consumption, and consumption of very hot drinks are risk factors for oesophageal squamous cell carcinoma.^27^, ^32^, ^36^

The poor prognosis of oesophageal cancer, indicated by the high MIR, might also call for screening efforts in the general population or in high-risk groups. Early detection of oesophageal cancer is also appealing because premalignant lesions and early cancers can now be treated with methods that are less invasive than surgery, such as endoscopic mucosal resection and endoscopic submucosal dissection, photodynamic therapy, argon plasma coagulation, radiofrequency ablation, and cryotherapy.^49^, ^50^ In some areas of China that have high incidence rates of oesophageal squamous cell carcinoma and its precursor lesions (ie, squamous dysplasia), mass upper gastrointestinal endoscopy has been shown to increase survival^51^ and to be highly cost-effective.^52^ However, in most other parts of the world, the prevalence rates of these precursor lesions are too low, even among cigarette smokers and alcohol drinkers, to merit upper gastrointestinal endoscopy and potentially invasive treatment. Other screening methods, such as cytological detection of dysplasia by use of balloon samplers, have shown low sensitivity and specificity for precursor lesions of oesophageal squamous cell carcinoma,^53^ resulting in high false positive and false negative rates. Due to its low prevalence and low progression rates, screening for oesophageal adenocarcinoma via its precursor lesion (Barrett's oesophagus) is not cost-effective.^54^ Early detection of oesophageal cancer might also occur as a byproduct of screening for gastric cancer. For example, in South Korea, mass screening for gastric cancer by use of upper endoscopy or upper gastrointestinal series, which is done as part of a national screening programme,^55^ might also help in early detection of oesophageal cancer. In some parts of the world, such as in China, incidence rates of both oesophageal cancer and gastric cancer are high, and mass endoscopic screening efforts might be beneficial to detect both cancers at early stages.^56^

Primary prevention of oesophageal cancer with chemopreventive measures has been the subject of many observational studies and randomised trials.^57^, ^58^ Long-term use of aspirin and other non-steroidal anti-inflammatory drugs, statins, and anti-reflux therapy have been suggested to reduce the risk of oesophageal adenocarcinoma,^57^ whereas supplemental selenium and other micronutrients might reduce the risk of oesophageal squamous cell carcinoma.^58^, ^59^ However, the level of evidence is not high enough to suggest widespread use of any of these drugs for primary prevention of oesophageal squamous cell carcinoma or oesophageal adenocarcinoma.^59^ The use of statins, however, has been growing over the past few decades, primarily because of their cardioprotective effects.

Presenting data from 195 countries and territories over three decades, this study is, to our knowledge, the most comprehensive and up-to-date analysis of the global burden, trends, and risk factors of oesophageal cancer. Linking data to Cancer Incidence in Five Continents provided, we believe for the first time, an opportunity to investigate associations with several potential risk factors of oesophageal squamous cell carcinoma at the country level. The data and findings, however, have several limitations. The two main histological subtypes of oesophageal cancer, oesophageal squamous cell carcinoma and oesophageal adenocarcinoma, have distinct risk factors, incidence trends, and geographical distributions,^22^ but data for these two subtypes are not currently captured independently in GBD. We suggest collection of data by histological subtype, where possible. By linking the GBD estimates to Cancer Incidence in Five Continents, we were able to find a partial solution to this limitation. As with other GBD cancer data, some countries did not have complete mortality or incidence data, and hence estimates were obtained by use of modelling. This modelling could be more problematic for oesophageal cancer than for other cancers, as rates of oesophageal squamous cell carcinoma might be drastically different across relatively short geographical distances. There is growing evidence that consumption of very hot drinks is a risk factor for oesophageal squamous cell carcinoma, particularly in very high-risk areas of the world, but GBD did not have data on this variable.

In conclusion, despite reductions in age-standardised incidence and mortality rates, oesophageal cancer remains a major cause of cancer mortality and burden across the world. The bulk of the burden of oesophageal cancer comes from east Asia, particularly from China. Areas with the highest age-standardised incidence, mortality, and DALY rates are located in east and central Asia and eastern, central, and southern sub-Saharan Africa. Although the exact reasons for these high rates are unclear, many epidemiological studies are ongoing, and we expect to see new findings over the next few years. In the meantime, intensive reduction of major known and potential oesophageal squamous cell carcinoma risk factors (primarily tobacco use, alcohol consumption, indoor air pollution, and consumption of very hot drinks) and oesophageal adenocarcinoma (primarily obesity and tobacco use) are strongly recommended. Where possible, it would be useful to collect country-level data on other risk factors such as consumption of opium and hot drinks, and water pipes (eg, hookahs). It would also be advisable to collect data on the histology of oesophageal cancer, and to treat oesophageal squamous cell carcinoma and oesophageal adenocarcinoma as two distinct entities for future analyses. High mortality-to-incidence ratios highlight the importance of primary and secondary prevention. Screening might be advised only in certain geographical areas with a very high risk of oesophageal squamous cell carcinoma and high prevalence of squamous dysplasia. Further development of less invasive screening methods should be highly encouraged.
