# Supplementary material for: The global, regional, and national burden of oesophageal cancer and its attributable risk factors in 195 countries and territories, 1990–2017: a systematic analysis for the Global Burden of Disease Study 2017
Source: Lancet Gastroenterol Hepatol. 2020 Apr 1;5(6):582–97. doi: 10.1016/S2468-1253(20)30007-8 (PMC7232026; doi:10.1016/S2468-1253(20)30007-8)
Supplement: Supplementary appendix [file mmc1.pdf]

THE LANCET
Gastroenterology & Hepatology

Supplementary appendix

This appendix formed part of the original submission and has been peer reviewed. We post it as supplied by the authors.

Supplement to: GBD 2017 Oesophageal Cancer Collaborators. The global, regional, and national burden of oesophageal cancer and its attributable risk factors in 195 countries and territories, 1990–2017: a systematic analysis for the Global Burden of Disease Study 2017. *Lancet Gastroenterol Hepatol* 2020; published online April 1. https://doi.org/10.1016/S2468-1253(20)30007-8.

Supplementary appendix

Supplement to: **Global, regional, and national burden of oesophageal cancer and its attributable risk factors, 1990–2017: a systematic analysis for the Global Burden of Disease Study 2017**

Contents:

Appendix Figure 1: Number of incident cases of oesophageal cancer for 21 Global Burden of Disease regions from 1990 to 2017

Appendix Figure 2: Number of deaths due to oesophageal cancer for 21 Global Burden of Disease regions from 1990 to 2017

Appendix Figure 3: The percentage change in age-standardised incidence rates of oesophageal cancer for 21 Global Burden of Disease regions, by sex, 1990–2017

Appendix Figure 4: The percentage change in age-standardised death rates of oesophageal cancer from 1990 to 2017 for 21 Global Burden of Disease regions, by sex

Appendix Figure 5: The percentage change in age-standardised incidence rates of oesophageal cancer for 195 countries and territories from 1990 to 2017

Appendix Figure 6: The percentage change in age-standardised death rates of oesophageal cancer for 195 countries and territories from 1990 to 2017

Appendix Figure 7: Global number of YLLs and YLDs and age-standardised rates of YLLs and YLDs per 100 000 population due to oesophageal cancer by age, 2017; Dotted and dashed lines indicate 95% upper and lower uncertainty intervals, respectively. YLDs=years lived with disability. YLLs=years of life lost.

Appendix Figure 8: Age-standardised incidence rates for oesophageal cancer for 21 Global Burden of Disease regions by Socio-demographic Index, 1990–2017; Expected values based on Socio-demographic Index and disease rates in all locations are shown as the black line. For each region, points from left to right depict estimates from each year from 1990 to 2017.

Appendix Figure 9: Age-standardised mortality rates for oesophageal cancer for 21 Global Burden of Disease regions by Socio-demographic Index, 1990–2017; Expected values based on Socio-demographic Index and disease rates in all locations are shown as the black line. For each region, points from left to right depict estimates from each year from 1990 to 2017.

Appendix Figure 10: The association between Sociodemographic index (SDI), Healthcare Access and Quality (HAQ) index, indoor air pollution, access to piped water, and proportion of oesophageal squamous cell carcinoma (OSCC) to oesophageal adenocarcinoma (OA) for 151 countries

Appendix Figure 11: The association between gastro-oesophageal reflux diseases and proportion of oesophageal squamous cell carcinoma (OSCC) to all oesophageal cancer (OC) for 151 countries

Appendix Table 1: Sequelae for oesophageal cancer and associated disability weights from Global Burden of Disease 2017.

Appendix Table 2: Incident cases of oesophageal cancer in 1990 and 2017 for both sexes and percentage change of age-standardised rates per 100,000 by location

Appendix Table 3: Deaths due to oesophageal cancer in 1990 and 2017 for both sexes and percentage change of age-standardised rates per 100,000 by location

Appendix Table 4: DALYs due to oesophageal cancer in 1990 and 2017 for both sexes and percentage change of age-standardised rates per 100,000 by location

Appendix Table 5. Country-level proportion of oesophageal squamous cell carcinoma (OSCC) to all oesophageal cancer (OC) cases, Socio-demographic Index (SDI), access to piped water, indoor air pollution index, and Healthcare Access and Quality (HAQ) Index. Results are sorted by OSCC to OC proportion.

Appendix Figure 1: Number of incident cases of oesophageal cancer for 21 Global Burden of Disease regions from 1990 to 2017

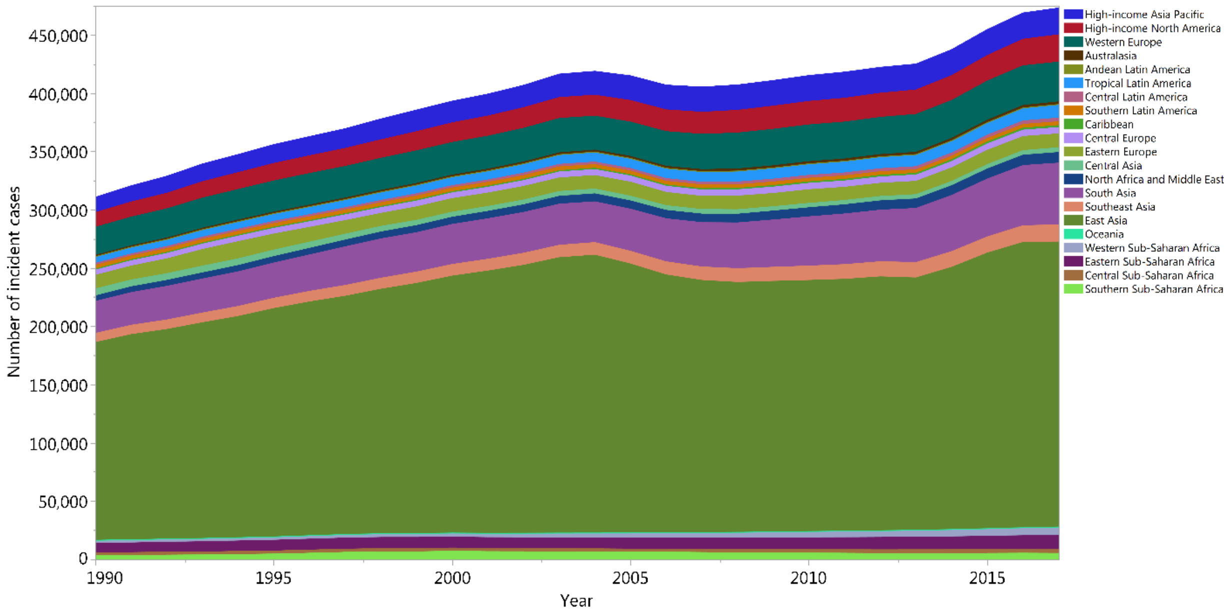

Appendix Figure 2: Number of deaths due to oesophageal cancer for 21 Global Burden of Disease regions from 1990 to 2017

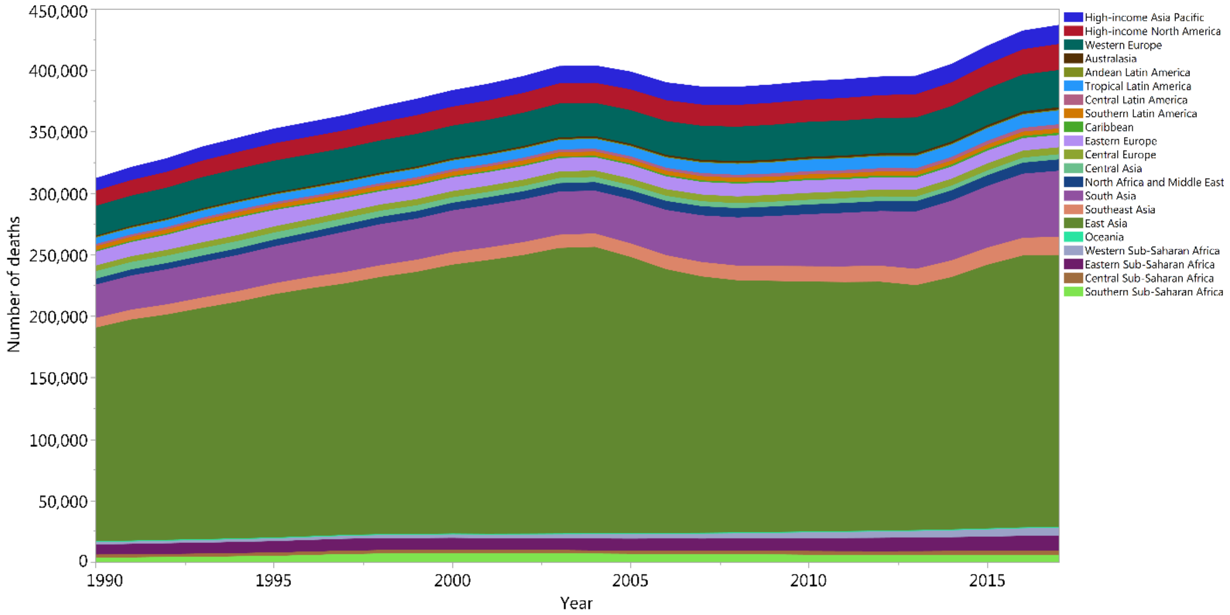

Appendix Figure 3: The percentage change in age-standardised incidence rates of oesophageal cancer for 21 Global Burden of Disease regions, by sex, 1990–2017

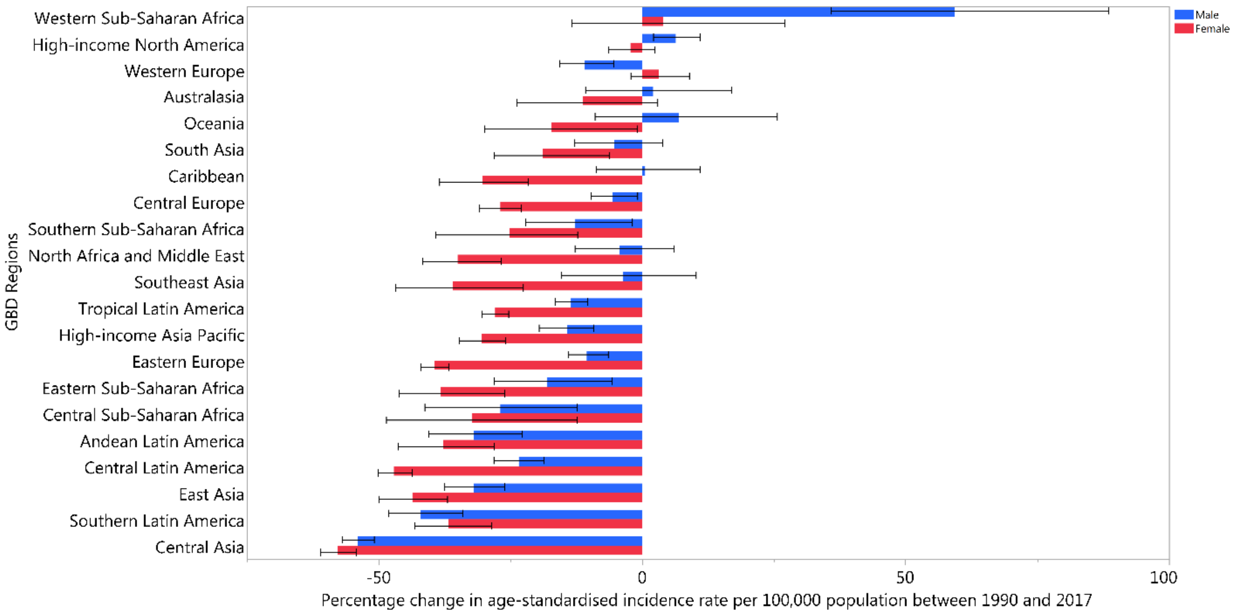

Appendix Figure 4: The percentage change in age-standardised death rates of oesophageal cancer from 1990 to 2017 for 21 Global Burden of Disease regions, by sex

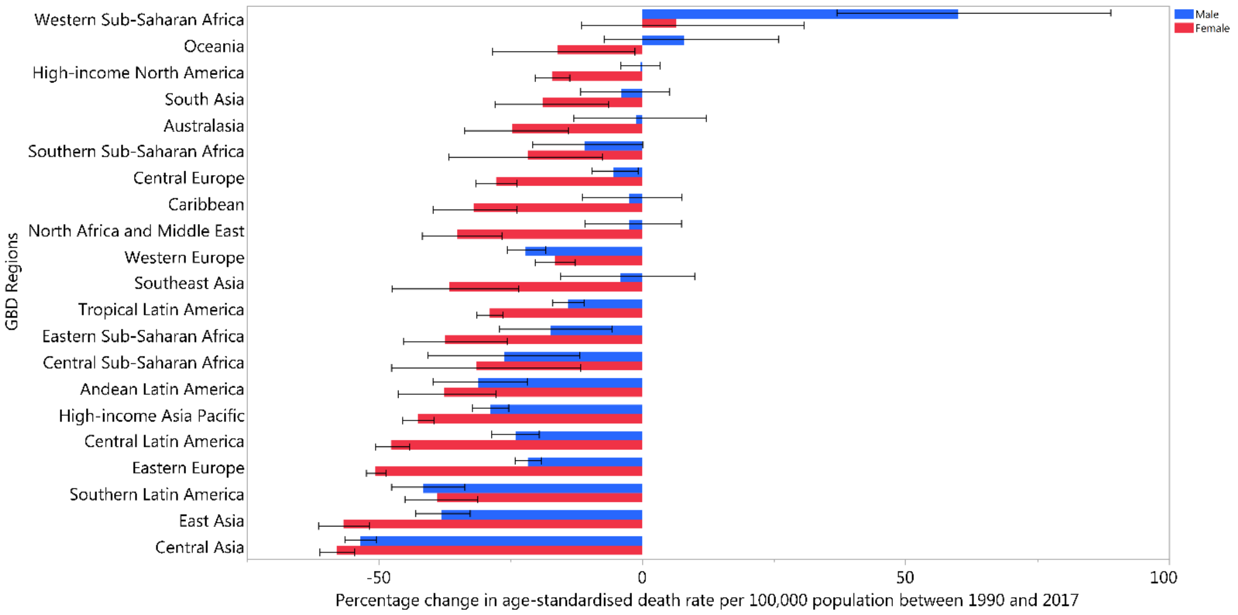

Appendix Figure 5: The percentage change in age-standardised incidence rates of oesophageal cancer for 195 countries and territories from 1990 to 2017

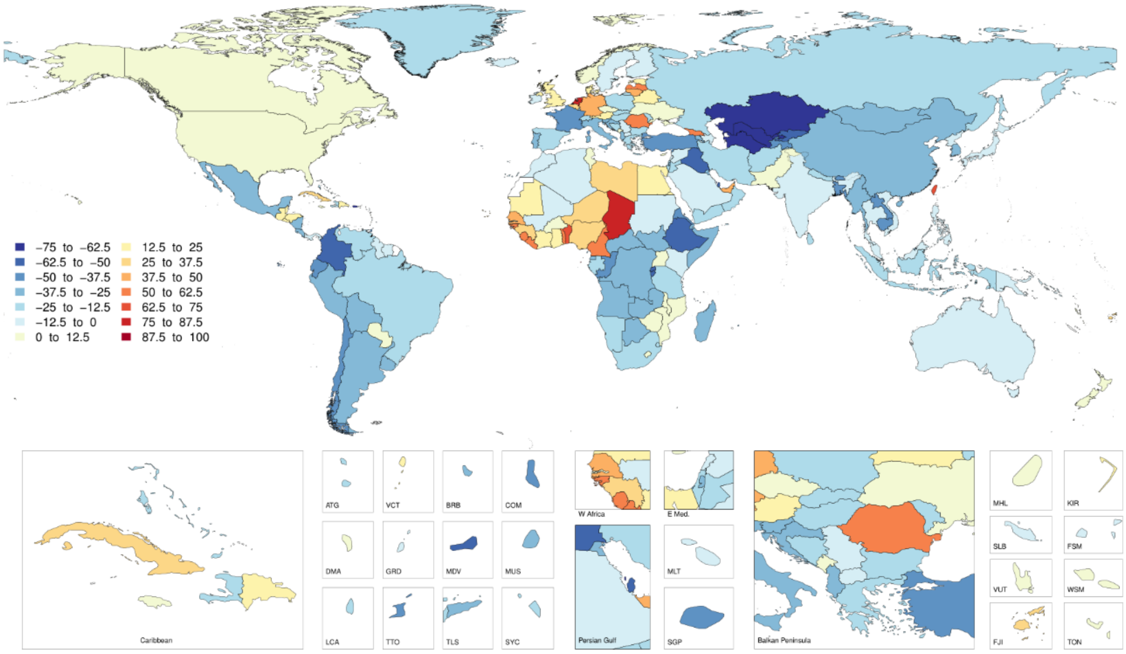

Appendix Figure 6: The percentage change in age-standardised death rates of oesophageal cancer for 195 countries and territories from 1990 to 2017

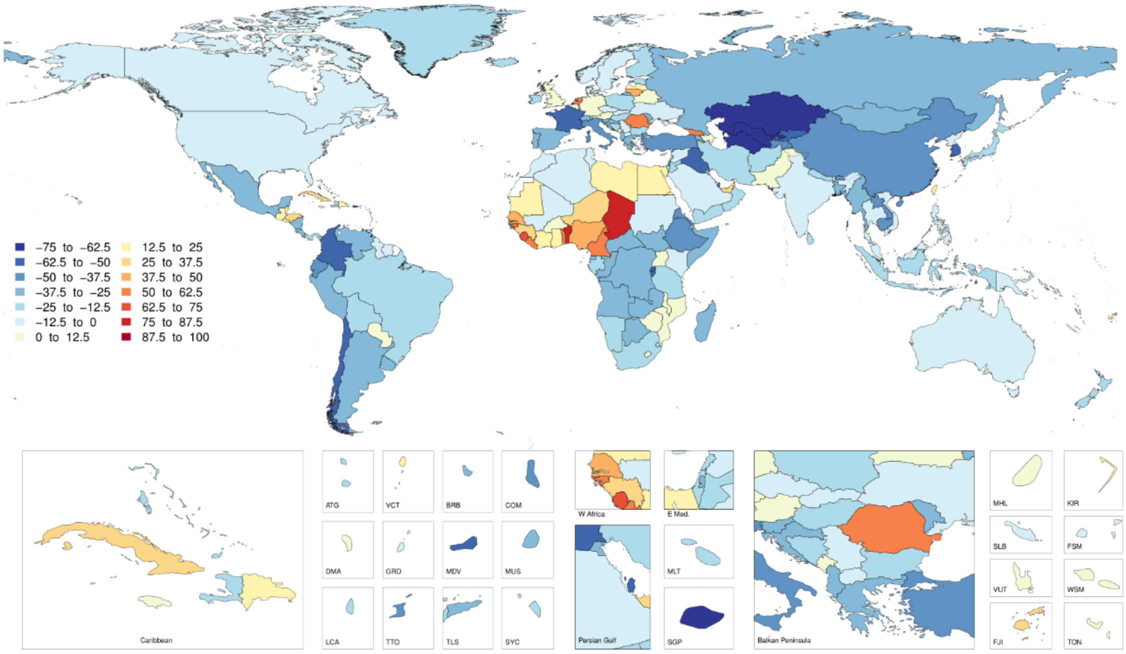

Appendix Figure 7: Global number of YLLs and YLDs and age-standardised rates of YLLs and YLDs per 100 000 population due to oesophageal cancer by age, 2017. Dotted and dashed lines indicate 95% upper and lower uncertainty intervals, respectively. YLDs=years lived with disability. YLLs=years of life lost.

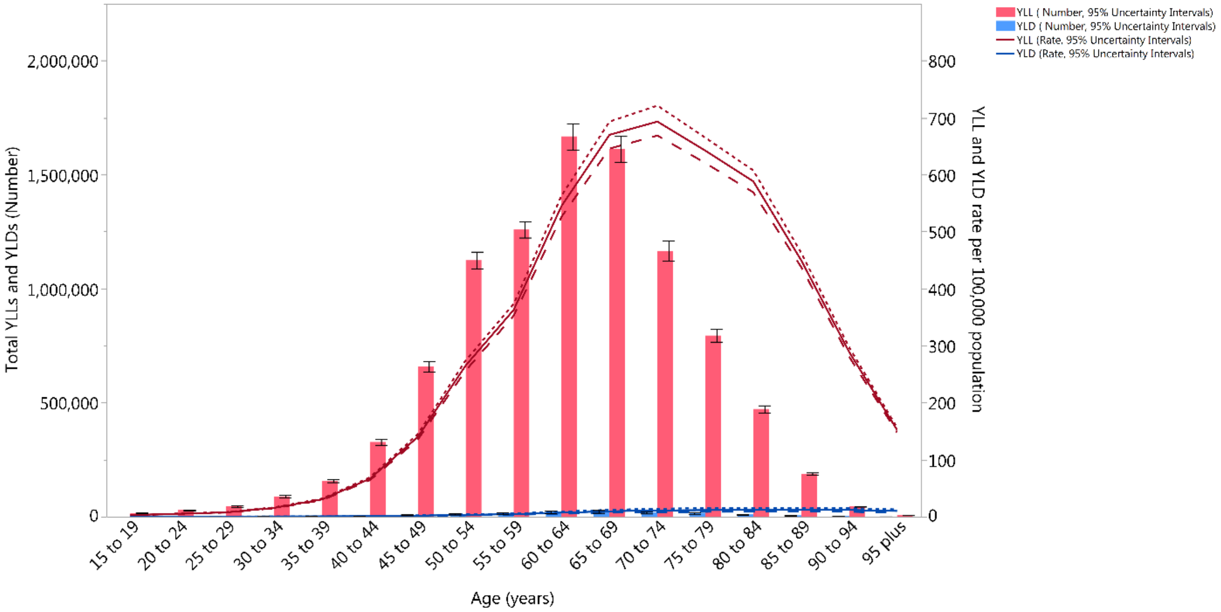

Appendix Figure 8: Age-standardised incidence rates for oesophageal cancer for 21 Global Burden of Disease regions by Socio-demographic Index, 1990–2017. Expected values based on Socio-demographic Index and disease rates in all locations are shown as the black line. For each region, points from left to right depict estimates from each year from 1990 to 2017.

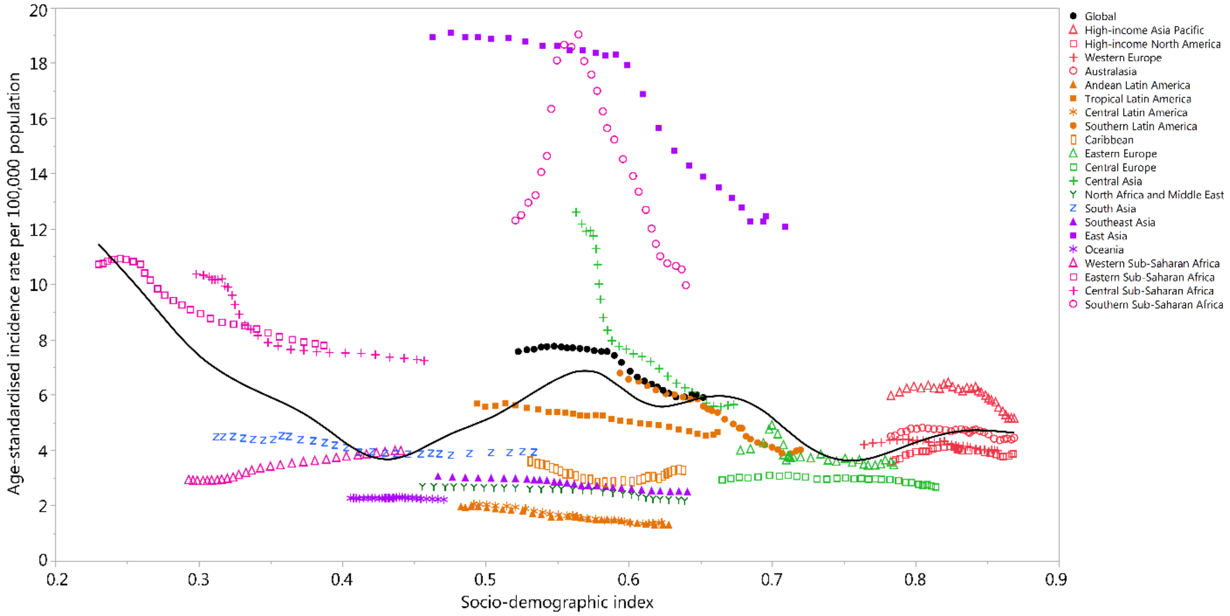

Appendix Figure 9: **Age-standardised mortality rates for oesophageal cancer for 21 Global Burden of Disease regions by Socio-demographic Index, 1990–2017**. Expected values based on Socio-demographic Index and disease rates in all locations are shown as the black line. For each region, points from left to right depict estimates from each year from 1990 to 2017.

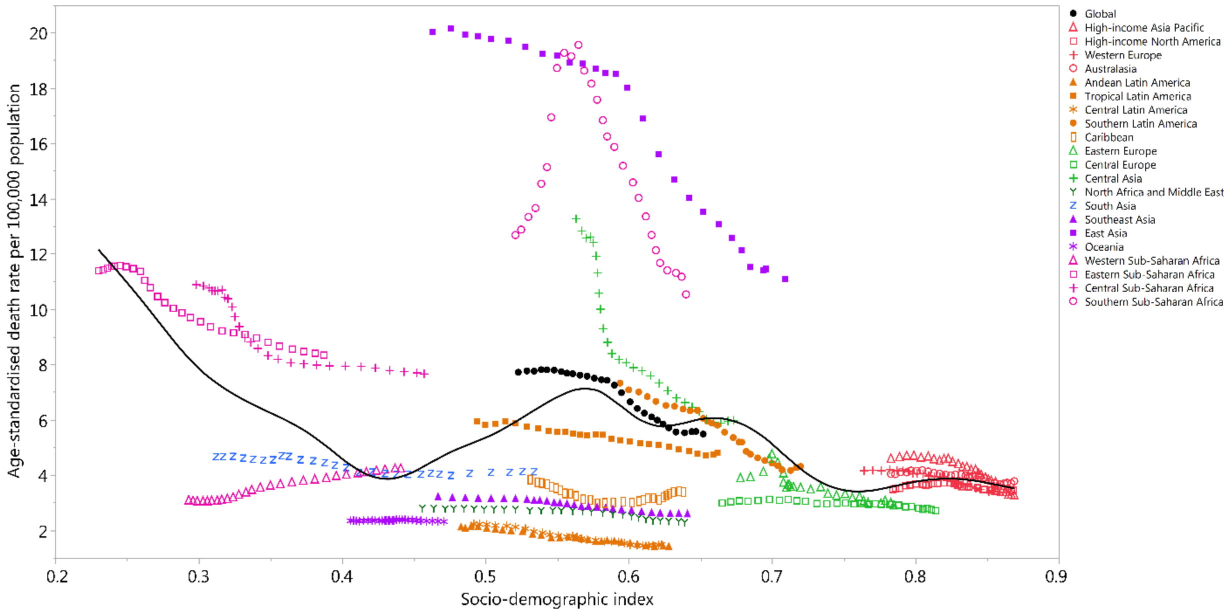

Appendix Figure 10: **The association between Sociodemographic index (SDI), Healthcare Access and Quality (HAQ) index, indoor air pollution, access to piped water, and proportion of oesophageal squamous cell carcinoma (OSCC) to oesophageal adenocarcinoma (OA) for 151 countries**

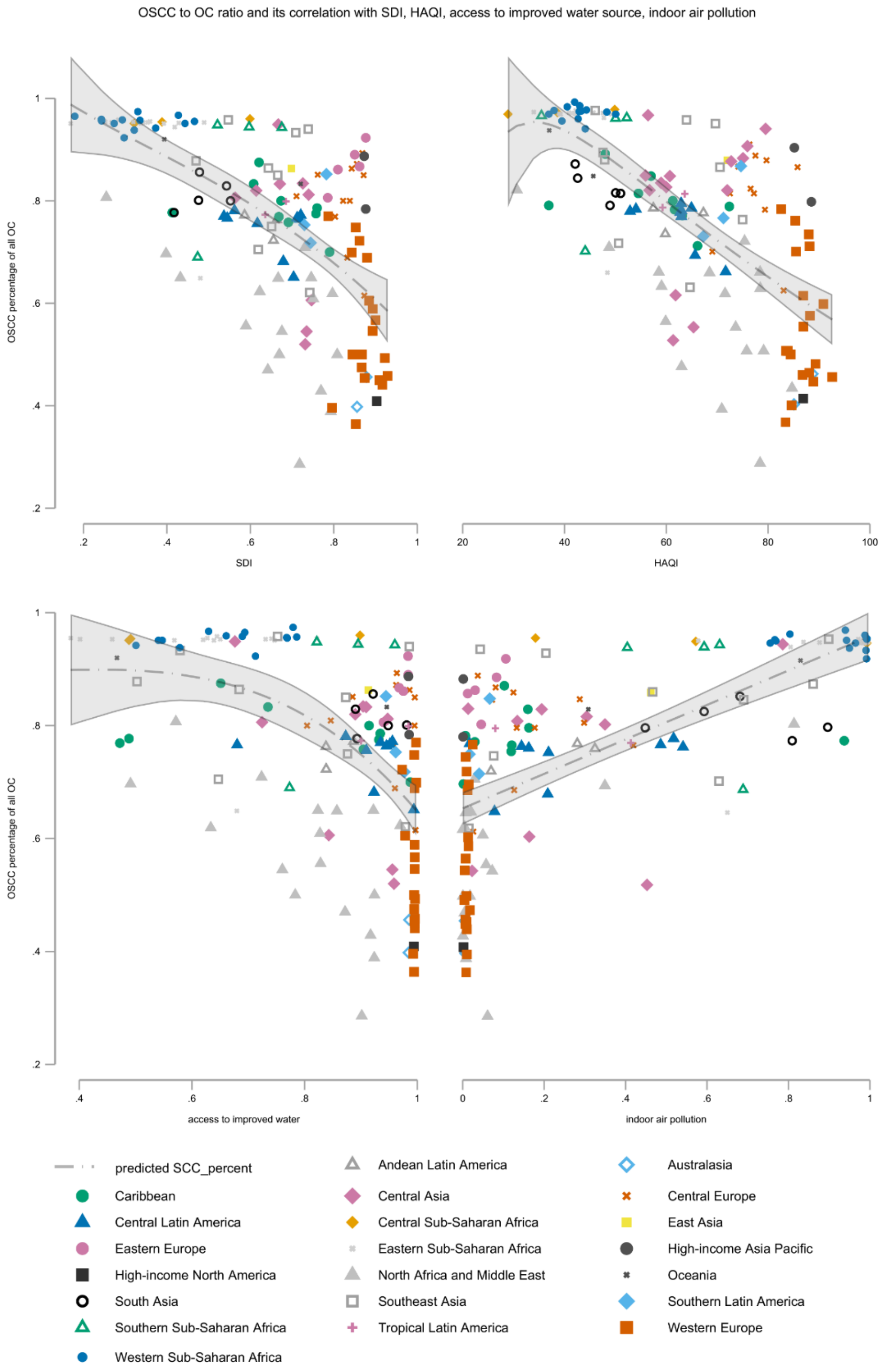

Appendix Figure 11: The association between gastro-oesophageal reflux diseases and proportion of oesophageal squamous cell carcinoma (OSCC) to all oesophageal cancer (OC) for 151 countries

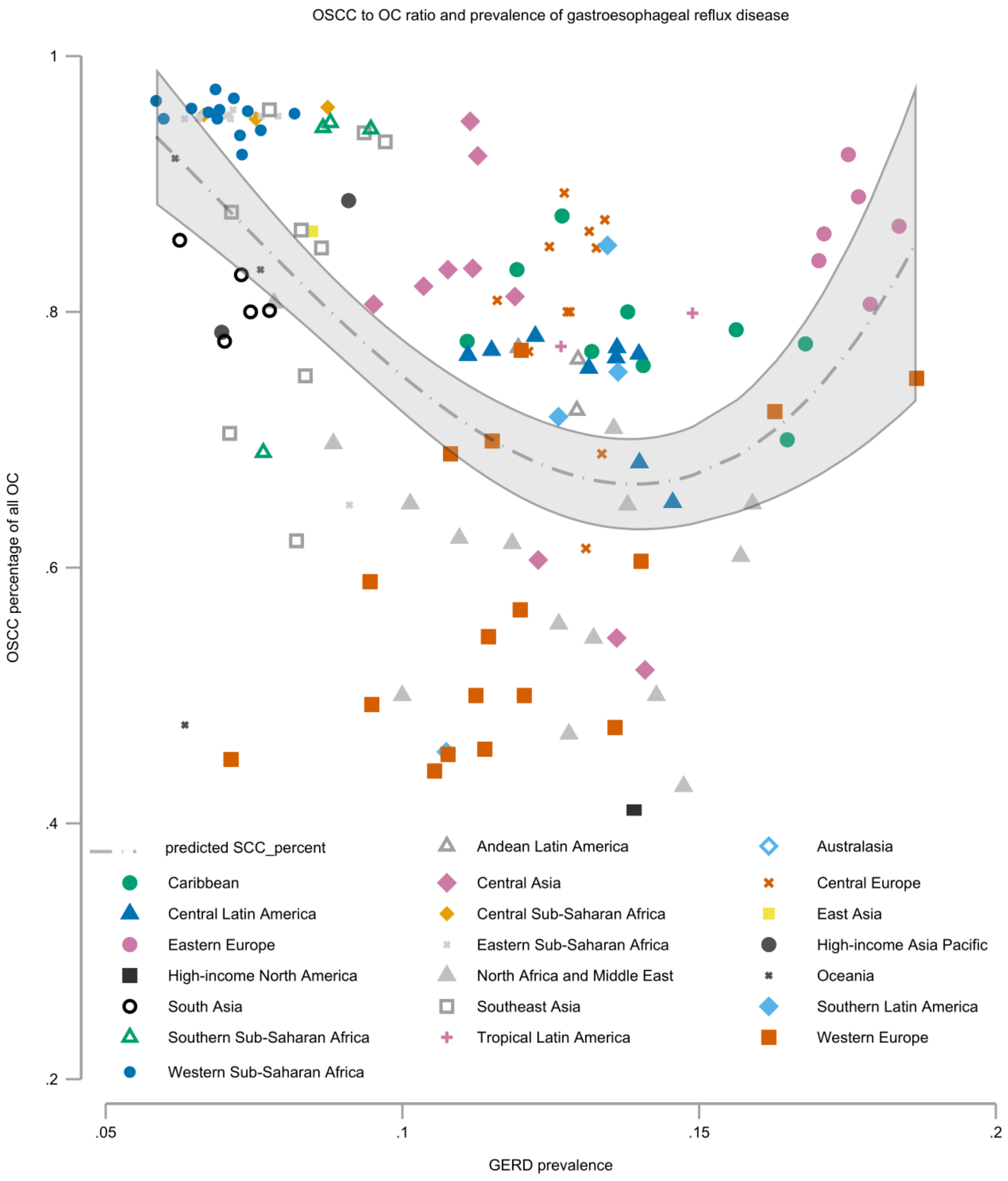

Appendix Table 1: **Sequelae for oesophageal cancer and associated disability weights from Global Burden of Disease 2017**

**Sequela**	**Health state name**	**Lay description**	**Disability weight (95% CI)**
Diagnosis and primary therapy phase	Cancer, diagnosis and primary therapy	Has pain, nausea, fatigue, weight loss and high anxiety.	0·288 (0·193–0·399)
Controlled phase	Generic uncomplicated disease: worry and daily medication	Has a chronic disease that requires medication every day and causes some worry but minimal interference with daily activities.	0·049 (0·031–0·072)
Metastatic phase	Cancer, metastatic	Has severe pain, extreme fatigue, weight loss, and high anxiety.	0·451 (0·307–0·600)
Terminal phase	Terminal phase, with medication (for cancers, end-stage kidney/liver disease)	Has lost a lot of weight and regularly uses strong medication to avoid constant pain. The person has no appetite, feels nauseated, and needs to spend most of the day in bed.	0·540 (0·377–0·687)

GBD=Global Burden of Disease. CI=Confidence interval

Appendix Table 2: Incident cases of oesophageal cancer in 1990 and 2017 for both sexes and percentage change of age-standardised rates per 100 000 by location

	1990		2017		Percentage change in age-standardised rates between 1990 and 2017
	Counts (95% UI)	Rate (95% UI)	Counts (95% UI)	Rate (95% UI)	
**Global**	**310236 (300690, 322028)**	**7·6 (7·3, 7·9)**	**472525 (459485, 485294)**	**5·9 (5·7, 6·1)**	**-22 (-25·2, -18·6)**
**High-income North America**	**12625 (12476, 12793)**	**3·6 (3·6, 3·7)**	**23420 (22732, 24124)**	**3·9 (3·8, 4)**	**7·4 (3·9, 11·1)**
Canada	1229 (1181, 1278)	3·7 (3·6, 3·9)	2722 (2472, 3004)	4·1 (3·7, 4·5)	10·6 (-0·4, 23·5)
Greenland	5 (4, 5)	13·2 (12·1, 14·4)	7 (7, 8)	10·8 (9·7, 11·8)	-18·7 (-28·8, -7·1)
USA	11391 (11248, 11556)	3·6 (3·6, 3·7)	20690 (20041, 21331)	3·9 (3·7, 4)	6·7 (3·1, 10·4)
**Australasia**	**1086 (1054, 1121)**	**4·5 (4·4, 4·7)**	**2165 (1961, 2382)**	**4·4 (4, 4·9)**	**-1·4 (-10·8, 9·2)**
Australia	901 (871, 934)	4·5 (4·3, 4·7)	1796 (1597, 2006)	4·4 (3·9, 4·9)	-2·6 (-13·6, 9·3)
New Zealand	185 (173, 197)	4·6 (4·3, 4·9)	370 (333, 410)	4·8 (4·3, 5·3)	5·5 (-6·6, 18·5)
**High-income Asia-Pacific**	**12361 (12150, 12560)**	**6 (5·9, 6·1)**	**22145 (20956, 23212)**	**5·1 (4·9, 5·4)**	**-13·8 (-18·5, -9·3)**
Brunei	4 (3, 4)	3·6 (3·2, 4)	7 (6, 8)	2·4 (2·1, 2·8)	-32·6 (-43·1, -20·5)
Japan	10667 (10485, 10848)	6·1 (6, 6·2)	19202 (18203, 20140)	5·8 (5·5, 6·1)	-4·3 (-9·4, 0·5)
Singapore	83 (78, 88)	3·7 (3·5, 3·9)	133 (118, 151)	1·9 (1·7, 2·2)	-47·7 (-54·4, -40·8)
South Korea	1608 (1541, 1679)	5 (4·8, 5·3)	2801 (2414, 3216)	3·2 (2·8, 3·7)	-35·6 (-45·3, -25·8)
**Western Europe**	**24078 (23712, 24466)**	**4·2 (4·2, 4·3)**	**34183 (32753, 35615)**	**4 (3·8, 4·2)**	**-5·2 (-9·6, -0·5)**
Andorra	2 (2, 2)	3·5 (3, 4·1)	5 (4, 6)	3·5 (2·9, 4·2)	1 (-18·5, 22·4)
Austria	254 (244, 267)	2·2 (2·1, 2·3)	453 (410, 501)	2·7 (2·5, 3)	23·1 (9·9, 38·4)
Belgium	504 (484, 524)	3·3 (3·2, 3·5)	892 (812, 977)	4·2 (3·8, 4·6)	25·5 (13·4, 39)
Cyprus	9 (8, 10)	1 (0·9, 1·2)	21 (19, 24)	1·1 (1, 1·3)	8·4 (-9·2, 31·5)
Denmark	338 (324, 352)	4·2 (4·1, 4·4)	537 (489, 584)	4·8 (4·4, 5·2)	14 (3·4, 25·6)
Finland	227 (215, 240)	3·1 (3, 3·3)	344 (312, 379)	2·9 (2·7, 3·3)	-6·2 (-16, 5·8)
France	5492 (5314, 5691)	7 (6·8, 7·2)	4550 (4150, 4973)	3·6 (3·3, 4)	-47·9 (-52·6, -42·4)
Germany	4195 (4011, 4411)	3·4 (3·3, 3·6)	8010 (7000, 9118)	4·8 (4·1, 5·4)	38·4 (19·9, 60·2)
Greece	243 (232, 254)	1·6 (1·5, 1·6)	280 (255, 309)	1·2 (1·1, 1·4)	-20·7 (-28·8, -11·6)
Iceland	13 (12, 14)	4·6 (4·3, 4·9)	23 (21, 26)	4·4 (4, 4·9)	-2·6 (-13·8, 11)
Ireland	264 (254, 275)	6·3 (6·1, 6·6)	443 (394, 490)	6·1 (5·4, 6·8)	-3·2 (-14·1, 7·9)
Israel	75 (70, 79)	1·5 (1·4, 1·6)	144 (131, 159)	1·3 (1·2, 1·4)	-17 (-25·1, -7·8)
Italy	2438 (2359, 2525)	2·8 (2·7, 2·9)	2468 (2242, 2728)	1·8 (1·7, 2)	-33·7 (-40·4, -25·8)
Luxembourg	23 (21, 24)	4·1 (3·9, 4·4)	34 (30, 38)	3·6 (3·1, 4·1)	-13·3 (-25·7, 1·3)

Malta	11<br>(10, 12)	2·5<br>(2·3, 2·7)	20<br>(18, 22)	2·4<br>(2·1, 2·6)	-5·8<br>(-15·9, 5·2)
Netherlands	782<br>(754, 813)	3·9<br>(3·8, 4·1)	2391<br>(2182, 2630)	7·3<br>(6·6, 8)	86·6<br>(68·2, 106·6)
Norway	164<br>(158, 172)	2·5<br>(2·4, 2·6)	251<br>(234, 269)	2·7<br>(2·5, 2·9)	11·3<br>(2·6, 20·9)
Portugal	573<br>(551, 597)	4·2<br>(4, 4·3)	649<br>(591, 717)	3·1<br>(2·8, 3·5)	-25·2<br>(-32·7, -16·5)
Spain	1902<br>(1824, 1988)	3·5<br>(3·4, 3·7)	2485<br>(2229, 2784)	2·8<br>(2·5, 3·2)	-20·1<br>(-29·2, -9·8)
Sweden	411<br>(395, 428)	2·7<br>(2·6, 2·8)	539<br>(502, 577)	2·6<br>(2·5, 2·8)	-2·4<br>(-9·9, 5·9)
Switzerland	403<br>(386, 420)	3·9<br>(3·8, 4·1)	548<br>(497, 616)	3·3<br>(3, 3·8)	-15·1<br>(-24·1, -4·2)
United Kingdom	5732<br>(5656, 5821)	6·2<br>(6·1, 6·3)	9062<br>(8824, 9315)	7·2<br>(7, 7·4)	16·4<br>(13, 20·2)
**Southern Latin America**	**3170<br>(3091, 3251)**	**6·8<br>(6·6, 7)**	**3315<br>(3074, 3632)**	**4<br>(3·7, 4·4)**	**-40·9<br>(-45·7, -34·8)**
Argentina	2151<br>(2081, 2227)	6·6<br>(6·4, 6·8)	2206<br>(1978, 2498)	4·1<br>(3·7, 4·7)	-37·1<br>(-44, -28·1)
Chile	675<br>(648, 700)	6·9<br>(6·6, 7·2)	809<br>(727, 906)	3·5<br>(3·1, 3·9)	-49·6<br>(-55·2, -43·2)
Uruguay	343<br>(328, 360)	8·6<br>(8·2, 9)	300<br>(265, 336)	5·6<br>(4·9, 6·3)	-35<br>(-43·1, -25·9)
**Eastern Europe**	**11636<br>(11286, 11990)**	**4<br>(3·9, 4·1)**	**11804<br>(11452, 12183)**	**3·5<br>(3·4, 3·6)**	**-13·3<br>(-16·3, -9·9)**
Belarus	351<br>(335, 368)	2·6<br>(2·5, 2·7)	515<br>(453, 587)	3·3<br>(2·9, 3·7)	24<br>(8·2, 42·6)
Estonia	62<br>(59, 66)	3<br>(2·8, 3·1)	88<br>(73, 107)	3·6<br>(3, 4·4)	22·6<br>(0·6, 50·3)
Latvia	101<br>(96, 107)	2·7<br>(2·6, 2·9)	153<br>(130, 180)	4·2<br>(3·5, 4·9)	53·3<br>(28, 81·4)
Lithuania	129<br>(123, 135)	2·7<br>(2·6, 2·9)	216<br>(196, 239)	4·1<br>(3·7, 4·5)	48·6<br>(33·7, 65·1)
Moldova	105<br>(99, 112)	2·2<br>(2·1, 2·4)	98<br>(89, 108)	1·7<br>(1·6, 1·9)	-23·8<br>(-31·9, -15·4)
Russia	8604<br>(8314, 8903)	4·6<br>(4·4, 4·7)	8372<br>(8169, 8580)	3·6<br>(3·5, 3·7)	-21·5<br>(-24·3, -18·6)
Ukraine	2284<br>(2177, 2396)	3·1<br>(3, 3·2)	2362<br>(2156, 2607)	3·2<br>(2·9, 3·5)	3·1<br>(-7·5, 14·6)
**Central Europe**	**4456<br>(4373, 4539)**	**2·9<br>(2·9, 3)**	**5462<br>(5250, 5679)**	**2·7<br>(2·6, 2·8)**	**-8·7<br>(-12·3, -4·8)**
Albania	43<br>(40, 46)	1·9<br>(1·8, 2·1)	55<br>(45, 69)	1·3<br>(1·1, 1·7)	-31·2<br>(-45·3, -13·6)
Bosnia and Herzegovina	105<br>(98, 113)	2·5<br>(2·3, 2·6)	123<br>(110, 138)	2·1<br>(1·8, 2·3)	-17·1<br>(-26·9, -5·7)
Bulgaria	282<br>(270, 295)	2·2<br>(2·1, 2·3)	241<br>(219, 265)	1·8<br>(1·6, 2)	-18·3<br>(-26·7, -8·7)
Croatia	256<br>(244, 269)	3·8<br>(3·6, 4)	209<br>(190, 228)	2·5<br>(2·3, 2·7)	-34·2<br>(-40·6, -27·4)
Czech Republic	408<br>(395, 423)	3<br>(2·9, 3·1)	598<br>(548, 654)	3<br>(2·8, 3·3)	1·5<br>(-7·5, 11·6)
Hungary	592<br>(567, 619)	4·1<br>(3·9, 4·3)	616<br>(565, 669)	3·5<br>(3·2, 3·8)	-14·8<br>(-22·5, -6·7)
Macedonia	25<br>(23, 27)	1·3<br>(1·2, 1·4)	43<br>(38, 48)	1·3<br>(1·1, 1·4)	-1·6<br>(-14·5, 11·9)
Montenegro	16<br>(14, 17)	2·4<br>(2·1, 2·7)	24<br>(21, 28)	2·4<br>(2·1, 2·8)	0·6<br>(-15, 20)
Poland	1628<br>(1575, 1681)	3·6<br>(3·5, 3·7)	1969<br>(1808, 2132)	2·9<br>(2·7, 3·2)	-19·3<br>(-26·6, -11·6)
Romania	451<br>(432, 473)	1·6<br>(1·5, 1·6)	797<br>(734, 866)	2·4<br>(2·2, 2·6)	52·3<br>(38·7, 66·7)

Serbia	311<br>(278, 348)	2·6<br>(2·3, 2·8)	374<br>(338, 413)	2·4<br>(2·2, 2·7)	-4·3<br>(-16·1, 9·4)
Slovakia	240<br>(225, 255)	4<br>(3·8, 4·3)	312<br>(275, 355)	3·5<br>(3·1, 4)	-13·4<br>(-24·7, -0·4)
Slovenia	99<br>(94, 104)	3·9<br>(3·7, 4·1)	102<br>(91, 112)	2·5<br>(2·3, 2·8)	-35·4<br>(-42·9, -27·9)
**Central Asia**	**6025<br>(5880, 6169)**	**12·6<br>(12·3, 12·9)**	**4115<br>(3915, 4321)**	**5·7<br>(5·4, 5·9)**	**-55·1<br>(-57·5, -52·5)**
Armenia	62<br>(57, 66)	2·2<br>(2·1, 2·4)	65<br>(60, 71)	1·6<br>(1·4, 1·7)	-30·7<br>(-37·6, -22·9)
Azerbaijan	426<br>(402, 451)	8·2<br>(7·7, 8·7)	731<br>(636, 860)	8·1<br>(7·1, 9·5)	-0·5<br>(-14·4, 17·7)
Georgia	103<br>(95, 111)	1·6<br>(1·5, 1·7)	147<br>(134, 159)	2·6<br>(2·4, 2·8)	58·8<br>(41·2, 77·1)
Kazakhstan	2314<br>(2235, 2396)	18<br>(17·4, 18·6)	1062<br>(980, 1149)	6·4<br>(6, 6·9)	-64·3<br>(-67·2, -61·4)
Kyrgyzstan	233<br>(219, 249)	7·5<br>(7·1, 8)	138<br>(129, 149)	3·4<br>(3·1, 3·6)	-55·4<br>(-59·3, -51·1)
Mongolia	251<br>(211, 296)	25·7<br>(21·6, 30·2)	333<br>(296, 374)	18·5<br>(16·4, 20·8)	-28·2<br>(-40·1, -13·4)
Tajikistan	286<br>(271, 301)	10<br>(9·4, 10·5)	263<br>(232, 297)	5<br>(4·4, 5·7)	-49·6<br>(-56·1, -42·2)
Turkmenistan	614<br>(593, 634)	31·1<br>(30, 32·1)	334<br>(303, 367)	8·7<br>(7·9, 9·6)	-71·9<br>(-74·6, -69·1)
Uzbekistan	1738<br>(1669, 1807)	14·9<br>(14·3, 15·5)	1041<br>(920, 1171)	4·7<br>(4·2, 5·3)	-68·1<br>(-71·7, -64·1)
**Central Latin America**	**1707<br>(1676, 1748)**	**2<br>(2, 2·1)**	**3162<br>(3017, 3297)**	**1·4<br>(1·3, 1·4)**	**-32·8<br>(-36, -29·6)**
Colombia	581<br>(559, 606)	3·4<br>(3·3, 3·5)	804<br>(713, 902)	1·5<br>(1·3, 1·7)	-56·2<br>(-61·1, -50·8)
Costa Rica	45<br>(43, 48)	2·6<br>(2·5, 2·7)	84<br>(76, 92)	1·7<br>(1·6, 1·9)	-33·9<br>(-40·8, -26·6)
El Salvador	38<br>(35, 41)	1·3<br>(1·2, 1·4)	81<br>(69, 96)	1·4<br>(1·2, 1·7)	13·7<br>(-5·3, 35·3)
Guatemala	48<br>(46, 51)	1·3<br>(1·3, 1·4)	168<br>(150, 188)	1·6<br>(1·4, 1·7)	17·2<br>(3·4, 33·6)
Honduras	26<br>(22, 29)	1·2<br>(1, 1·4)	86<br>(68, 108)	1·5<br>(1·2, 1·8)	22·6<br>(-4·8, 60·5)
Mexico	713<br>(696, 732)	1·6<br>(1·6, 1·7)	1377<br>(1329, 1418)	1·2<br>(1·2, 1·3)	-26·2<br>(-29·2, -23·5)
Nicaragua	16<br>(15, 18)	1<br>(0·9, 1·1)	32<br>(28, 36)	0·7<br>(0·6, 0·8)	-31·4<br>(-42·3, -18·4)
Panama	22<br>(21, 24)	1·5<br>(1·4, 1·6)	53<br>(48, 58)	1·3<br>(1·2, 1·5)	-11<br>(-21·1, 0·9)
Venezuela	218<br>(207, 228)	2·3<br>(2·2, 2·4)	477<br>(406, 554)	1·7<br>(1·5, 2)	-24·5<br>(-36·7, -11·4)
**Andean Latin America**	**403<br>(382, 427)**	**2<br>(1·9, 2·1)**	**699<br>(631, 767)**	**1·3<br>(1·2, 1·4)**	**-33·5<br>(-40·4, -26·5)**
Bolivia	96<br>(82, 110)	3<br>(2·6, 3·4)	185<br>(146, 234)	2·2<br>(1·8, 2·8)	-25·8<br>(-43·4, -6·2)
Ecuador	116<br>(110, 122)	2·2<br>(2·1, 2·3)	189<br>(169, 211)	1·3<br>(1·2, 1·5)	-40·2<br>(-46·9, -32·1)
Peru	191<br>(174, 210)	1·6<br>(1·4, 1·8)	326<br>(276, 377)	1·1<br>(0·9, 1·2)	-33·1<br>(-44·2, -19)
**Caribbean**	**949<br>(909, 1000)**	**3·6<br>(3·5, 3·8)**	**1684<br>(1546, 1844)**	**3·3<br>(3, 3·6)**	**-8·9<br>(-16, -1·3)**
Antigua and Barbuda	2<br>(1, 2)	3·1<br>(2·9, 3·4)	3<br>(2, 3)	2·6<br>(2·4, 2·9)	-16·4<br>(-25·4, -5·6)
The Bahamas	9<br>(8, 11)	5·9<br>(5·3, 6·5)	18<br>(16, 21)	4·7<br>(4·2, 5·3)	-19·7<br>(-31·8, -3·8)
Barbados	15<br>(14, 17)	5·2<br>(4·9, 5·6)	19<br>(17, 21)	3·9<br>(3·5, 4·3)	-26·1<br>(-34·4, -15·9)

Belize	2<br>(2, 2)	1·8<br>(1·6, 2)	6<br>(5, 6)	2·2<br>(2, 2·4)	23·4<br>(7·8, 42·3)
Bermuda	4<br>(4, 4)	6·3<br>(5·8, 6·8)	5<br>(5, 6)	4·1<br>(3·7, 4·5)	-34·8<br>(-42·5, -26)
Cuba	360<br>(347, 375)	3·5<br>(3·3, 3·6)	862<br>(753, 979)	4·6<br>(4, 5·3)	33·8<br>(16, 52·9)
Dominica	3<br>(3, 3)	3·9<br>(3·6, 4·2)	4<br>(3, 4)	4<br>(3·6, 4·5)	4·2<br>(-8·3, 16·6)
Dominican Republic	59<br>(53, 66)	1·5<br>(1·4, 1·7)	172<br>(145, 200)	1·9<br>(1·6, 2·2)	22·6<br>(0·5, 48·8)
Grenada	4<br>(4, 5)	6·2<br>(5·7, 6·6)	8<br>(7, 9)	5·5<br>(5, 6·1)	-10·2<br>(-20·8, 0·9)
Guyana	7<br>(6, 7)	1·8<br>(1·7, 1·9)	10<br>(9, 12)	1·6<br>(1·4, 1·9)	-7·4<br>(-19·7, 6·9)
Haiti	148<br>(112, 191)	4·5<br>(3·4, 5·8)	229<br>(169, 315)	3·5<br>(2·6, 4·8)	-22·9<br>(-40·1, 0·8)
Jamaica	53<br>(49, 58)	2·9<br>(2·7, 3·2)	88<br>(73, 104)	3·1<br>(2·6, 3·6)	5·3<br>(-13·2, 27·1)
Puerto Rico	218<br>(209, 228)	5·9<br>(5·6, 6·1)	149<br>(135, 164)	2·2<br>(2, 2·4)	-63·2<br>(-66·8, -59)
Saint Lucia	4<br>(4, 5)	4·7<br>(4·4, 5)	8<br>(7, 9)	3·7<br>(3·4, 4·1)	-20·7<br>(-29·8, -10·1)
Saint Vincent and the Grenadines	1<br>(1, 2)	2<br>(1·9, 2·2)	3<br>(3, 4)	2·3<br>(2·1, 2·6)	14·2<br>(-2, 32·1)
Suriname	3<br>(3, 4)	1·3<br>(1·2, 1·4)	8<br>(7, 9)	1·3<br>(1·2, 1·5)	-0·2<br>(-15·3, 16·8)
Trinidad and Tobago	20<br>(18, 21)	2·3<br>(2·1, 2·5)	25<br>(20, 31)	1·4<br>(1·1, 1·7)	-39·6<br>(-51·5, -24·3)
Virgin Islands	3<br>(3, 3)	3·4<br>(3, 3·8)	7<br>(6, 8)	3·9<br>(3·1, 4·4)	14·4<br>(-8·7, 35·8)
**Tropical Latin America**	**5349<br>(5237, 5464)**	**5·7<br>(5·6, 5·8)**	**10981<br>(10709, 11257)**	**4·6<br>(4·5, 4·8)**	**-18·2<br>(-20·6, -15·8)**
Brazil	5281<br>(5170, 5394)	5·7<br>(5·6, 5·9)	10801<br>(10536, 11086)	4·7<br>(4·6, 4·8)	-18·6<br>(-21, -16·1)
Paraguay	69<br>(61, 75)	3·2<br>(2·9, 3·5)	179<br>(146, 219)	3·4<br>(2·8, 4·2)	8·5<br>(-12·4, 36)
**East Asia**	**169650<br>(162162, 179419)**	**18·9<br>(18·1, 20)**	**244509<br>(232910, 256226)**	**12·1<br>(11·5, 12·7)**	**-36·1<br>(-40·9, -31·3)**
China	164473<br>(157194, 173853)	19·4<br>(18·5, 20·5)	234624<br>(223240, 246036)	12·2<br>(11·6, 12·8)	-36·9<br>(-41·7, -32·1)
North Korea	1446<br>(1155, 1762)	8·7<br>(7, 10·5)	2575<br>(1934, 3195)	8·1<br>(6·1, 10·1)	-6·2<br>(-27·2, 18·8)
Taiwan (Province of China)	905<br>(874, 940)	5·5<br>(5·3, 5·7)	3372<br>(3003, 3813)	8·9<br>(7·9, 10·1)	62·5<br>(43·4, 85·4)
**Southeast Asia**	**7898<br>(7074, 8600)**	**3·1<br>(2·7, 3·3)**	**14755<br>(13824, 15840)**	**2·5<br>(2·4, 2·7)**	**-18·1<br>(-25·9, -6·4)**
Cambodia	212<br>(179, 247)	4·5<br>(3·8, 5·2)	289<br>(238, 354)	2·5<br>(2·1, 3·1)	-43·4<br>(-54·6, -29·3)
Indonesia	920<br>(829, 1049)	0·9<br>(0·8, 1·1)	1700<br>(1578, 1825)	0·8<br>(0·7, 0·9)	-12·9<br>(-26, -0·5)
Laos	101<br>(81, 121)	4·7<br>(3·8, 5·5)	105<br>(80, 134)	2·5<br>(1·9, 3·1)	-46·7<br>(-58·4, -32·6)
Malaysia	253<br>(232, 276)	2·9<br>(2·6, 3·1)	648<br>(576, 740)	2·6<br>(2·4, 3)	-7·3<br>(-21·3, 11·4)
Maldives	3<br>(2, 4)	3·6<br>(2·7, 4·4)	4<br>(4, 4)	1·5<br>(1·3, 1·6)	-59·2<br>(-67·9, -43·1)
Mauritius	22<br>(21, 23)	3<br>(2·8, 3·2)	36<br>(33, 40)	2·2<br>(2, 2·4)	-26·1<br>(-33·9, -17·6)
Myanmar	2875<br>(2299, 3496)	12·3<br>(9·9, 14·9)	3948<br>(3439, 4599)	8·9<br>(7·8, 10·3)	-27·4<br>(-43·5, -6·8)
Philippines	427<br>(400, 455)	1·4<br>(1·3, 1·5)	882<br>(765, 1001)	1·2<br>(1·1, 1·4)	-11·6<br>(-23·6, 2·5)

Sri Lanka	500<br>(454, 547)	4·5<br>(4·1, 4·9)	969<br>(809, 1163)	3·9<br>(3·3, 4·7)	-12·7<br>(-29·1, 7·4)
Seychelles	3<br>(3, 4)	6<br>(5·4, 6·6)	6<br>(5, 6)	5·1<br>(4·6, 5·6)	-14·8<br>(-26·8, -1·6)
Thailand	1075<br>(985, 1171)	3<br>(2·8, 3·3)	2942<br>(2576, 3346)	2·9<br>(2·6, 3·3)	-2·5<br>(-16·5, 14·1)
East Timor	9<br>(7, 11)	3·1<br>(2·5, 3·7)	16<br>(13, 21)	2·1<br>(1·6, 2·7)	-31·9<br>(-47·3, -13·7)
Vietnam	1488<br>(1222, 1774)	3·7<br>(3, 4·3)	3190<br>(2669, 3918)	3·4<br>(2·9, 4·1)	-7·7<br>(-29·2, 24)
**Oceania**	**68<br>(59, 79)**	**2·3<br>(2, 2·6)**	**145<br>(123, 171)**	**2·2<br>(1·9, 2·5)**	**-2·2<br>(-13·5, 10·6)**
American Samoa	0<br>(0, 0)	1·2<br>(1, 1·3)	1<br>(0, 1)	1·3<br>(1·1, 1·5)	13·4<br>(-6·8, 35·5)
Federated States of Micronesia	1<br>(1, 1)	2·4<br>(2, 2·8)	1<br>(1, 2)	2·1<br>(1·7, 2·5)	-12·3<br>(-30·6, 9·6)
Fiji	6<br>(6, 7)	1·9<br>(1·6, 2·1)	16<br>(14, 19)	2·4<br>(2·1, 2·7)	29·7<br>(4·9, 60)
Guam	1<br>(1, 2)	1·9<br>(1·7, 2·1)	5<br>(4, 5)	2·5<br>(2·2, 2·8)	32·4<br>(12·1, 54·9)
Kiribati	1<br>(1, 1)	3·6<br>(3·2, 4)	3<br>(2, 3)	4·2<br>(3·6, 4·9)	15·4<br>(-4·7, 40·1)
Marshall Islands	1<br>(0, 1)	3·2<br>(2·7, 3·8)	1<br>(1, 1)	3·2<br>(2·7, 3·9)	1<br>(-16·3, 22·1)
Northern Mariana Islands	0<br>(0, 0)	1·4<br>(1·2, 1·7)	1<br>(1, 1)	1·7<br>(1·5, 2)	18·5<br>(-5·1, 45·9)
Papua New Guinea	45<br>(37, 55)	2·3<br>(1·9, 2·8)	96<br>(76, 121)	2·1<br>(1·7, 2·6)	-8·7<br>(-24·4, 9·9)
Samoa	1<br>(1, 2)	1·5<br>(1·2, 1·9)	2<br>(2, 3)	1·5<br>(1·2, 2)	0·7<br>(-19·2, 23·6)
Solomon Islands	3<br>(3, 4)	2·4<br>(1·9, 2·9)	7<br>(5, 8)	2·2<br>(1·7, 2·6)	-8·6<br>(-24·9, 10·5)
Tonga	1<br>(1, 1)	2·2<br>(1·9, 2·6)	2<br>(2, 2)	2·4<br>(2, 2·8)	5·3<br>(-14·4, 31·4)
Vanuatu	1<br>(1, 2)	2·1<br>(1·5, 2·6)	3<br>(2, 4)	2·1<br>(1·6, 2·6)	0<br>(-20·7, 26)
**North Africa and Middle East**	**4763<br>(4261, 5246)**	**2·7<br>(2·4, 3)**	**9080<br>(8456, 9833)**	**2·2<br>(2, 2·4)**	**-19·2<br>(-25·2, -11·6)**
Afghanistan	698<br>(417, 920)	9·6<br>(5·9, 12·6)	889<br>(682, 1113)	8·1<br>(6·3, 10·3)	-15·7<br>(-34·6, 35·3)
Algeria	133<br>(118, 148)	1·1<br>(1, 1·2)	305<br>(273, 339)	0·9<br>(0·9, 1·1)	-11·5<br>(-24·6, 2)
Bahrain	6<br>(5, 7)	3·6<br>(3·2, 3·9)	10<br>(9, 12)	1·2<br>(1, 1·3)	-67·1<br>(-71·7, -61·6)
Egypt	440<br>(401, 482)	1·5<br>(1·4, 1·7)	1068<br>(939, 1219)	1·8<br>(1·6, 2)	16·5<br>(-0·9, 35·9)
Iran	1252<br>(1155, 1371)	4·9<br>(4·5, 5·4)	2661<br>(2560, 2822)	4<br>(3·9, 4·2)	-18·3<br>(-25·1, -11)
Iraq	117<br>(99, 136)	1·5<br>(1·3, 1·7)	154<br>(140, 170)	0·6<br>(0·6, 0·7)	-56<br>(-63·5, -46·7)
Jordan	18<br>(15, 20)	1·2<br>(1·1, 1·4)	56<br>(47, 65)	1<br>(0·9, 1·1)	-18·3<br>(-32·9, 1·8)
Kuwait	10<br>(9, 10)	1·5<br>(1·4, 1·6)	24<br>(22, 27)	1<br>(0·9, 1·1)	-32·2<br>(-40·2, -23·5)
Lebanon	30<br>(25, 36)	1·4<br>(1·1, 1·6)	71<br>(62, 81)	1·2<br>(1·1, 1·4)	-11·5<br>(-28·2, 16·2)
Libya	26<br>(21, 31)	1·3<br>(1·1, 1·6)	77<br>(62, 94)	1·7<br>(1·4, 2)	25<br>(-5·3, 61·9)
Morocco	192<br>(167, 219)	1·3<br>(1·1, 1·5)	389<br>(323, 459)	1·2<br>(1, 1·4)	-6<br>(-25, 18·8)
Palestine	11<br>(9, 14)	1·3<br>(1, 1·5)	23<br>(20, 25)	0·9<br>(0·8, 1)	-26<br>(-40·2, -8)

Oman	18 (15, 22)	2·6 (2·1, 3·2)	45 (36, 54)	2·3 (1·9, 2·7)	-13·7 (-34·1, 12·1)
Qatar	5 (4, 5)	4·8 (4·1, 5·5)	16 (13, 19)	2·2 (1·8, 2·6)	-53·6 (-63·8, -40·9)
Saudi Arabia	126 (104, 154)	2·1 (1·7, 2·5)	298 (255, 355)	2 (1·7, 2·3)	-5·7 (-25·4, 20)
Sudan	462 (333, 638)	4·9 (3·5, 6·9)	787 (554, 1065)	4·4 (3·1, 6)	-8·9 (-29·3, 16)
Syria	52 (46, 61)	1 (0·9, 1·1)	122 (98, 149)	1 (0·8, 1·2)	-1·7 (-24·7, 27·3)
Tunisia	42 (38, 46)	0·9 (0·8, 0·9)	102 (81, 124)	0·9 (0·7, 1)	0·2 (-21·6, 25·8)
Turkey	848 (763, 943)	2·2 (2, 2·5)	1139 (1034, 1257)	1·3 (1·2, 1·4)	-42·1 (-49·9, -33·3)
United Arab Emirates	29 (20, 39)	6·1 (4·3, 8·4)	344 (251, 469)	8·5 (6·3, 11·7)	38·5 (4, 88·9)
Yemen	246 (162, 347)	4·8 (3·2, 6·9)	493 (361, 659)	3·9 (2·9, 5·1)	-19·1 (-42·3, 17·1)
**South Asia**	**27371 (25764, 28932)**	**4·5 (4·2, 4·8)**	**53022 (50092, 56177)**	**3·9 (3·7, 4·2)**	**-12·3 (-18·4, -4·7)**
Bangladesh	2096 (1841, 2447)	4·2 (3·7, 4·9)	2959 (2550, 3402)	2·5 (2·2, 2·9)	-39·2 (-50·9, -26)
Bhutan	20 (16, 24)	7·5 (6·2, 9·2)	26 (21, 33)	4·3 (3·5, 5·4)	-42·6 (-57·4, -23·5)
India	20479 (19243, 21660)	4·1 (3·9, 4·4)	40072 (37723, 42361)	3·7 (3·4, 3·9)	-11·5 (-17·3, -4·5)
Nepal	620 (524, 745)	6·2 (5·2, 7·5)	1062 (872, 1267)	4·9 (4·1, 5·8)	-20·2 (-35·1, -3·5)
Pakistan	4157 (3649, 4744)	7·1 (6·3, 8·1)	8903 (7372, 10647)	7·8 (6·5, 9·4)	10·1 (-11·9, 36·9)
**Southern sub-Saharan Africa**	**3553 (3276, 3855)**	**12·3 (11·3, 13·4)**	**5484 (5205, 5746)**	**10 (9·5, 10·4)**	**-19·1 (-26·1, -10·7)**
Botswana	69 (55, 85)	11·6 (9·4, 14·1)	99 (83, 118)	7·5 (6·4, 9)	-35 (-48·2, -17·8)
Lesotho	134 (114, 155)	13·2 (11·2, 15·2)	168 (141, 197)	13·9 (11·6, 16·2)	5·5 (-14·9, 29·1)
Namibia	27 (23, 31)	3·6 (3·1, 4·1)	41 (35, 48)	2·9 (2·5, 3·3)	-20 (-34·5, -2·8)
South Africa	2725 (2497, 2975)	12·4 (11·3, 13·5)	4133 (3950, 4323)	9·5 (9, 9·9)	-23·4 (-30·4, -15·2)
Swaziland	54 (44, 66)	17·6 (14·3, 21·4)	86 (67, 106)	14·9 (11·6, 18·1)	-15·6 (-35·1, 6·8)
Zimbabwe	545 (467, 627)	12·8 (11·1, 14·7)	958 (806, 1130)	13·8 (11·8, 16·3)	7·7 (-14·1, 35·5)
**Western sub-Saharan Africa**	**2609 (2277, 3055)**	**2·9 (2·6, 3·4)**	**6901 (6007, 8208)**	**4 (3·5, 4·7)**	**35·7 (15·6, 58·9)**
Benin	73 (64, 84)	3·6 (3·1, 4·1)	286 (228, 362)	6·3 (5·1, 7·8)	74·6 (38·9, 124·2)
Burkina Faso	253 (199, 297)	6·2 (4·9, 7·2)	519 (438, 608)	6·4 (5·4, 7·4)	3·3 (-17·8, 34·1)
Cameroon	201 (155, 236)	4·4 (3·4, 5·2)	759 (584, 938)	6·8 (5·3, 8·3)	52·6 (18·5, 92·7)
Cape Verde	18 (16, 19)	7·7 (7, 8·4)	44 (38, 50)	10·1 (8·9, 11·5)	30·7 (9·3, 52·7)
Chad	82 (69, 97)	2·9 (2·4, 3·4)	279 (220, 352)	5·3 (4·2, 6·6)	84·1 (46·4, 128·9)
Côte d'Ivoire	42 (36, 49)	1 (0·9, 1·2)	125 (101, 155)	1·3 (1, 1·6)	21·2 (-4·7, 52·2)
The Gambia	7 (6, 8)	1·9 (1·5, 2·2)	19 (16, 23)	2·1 (1·7, 2·5)	12·1 (-11·2, 41·7)
Ghana	213 (179, 254)	3·4 (2·9, 4)	573 (491, 659)	3·8 (3·3, 4·4)	13·3 (-9·9, 42·1)

Guinea	60<br>(53, 67)	1·7<br>(1·5, 2)	124<br>(101, 151)	2·3<br>(1·9, 2·8)	32·2<br>(6·5, 65·6)
Guinea-Bissau	22<br>(18, 26)	5·3<br>(4·5, 6·2)	56<br>(45, 71)	8<br>(6·5, 10)	51·8<br>(20·3, 96·4)
Liberia	44<br>(37, 51)	3·8<br>(3·2, 4·4)	107<br>(85, 133)	5·7<br>(4·6, 7)	50·1<br>(19·7, 91·4)
Mali	107<br>(94, 122)	2·5<br>(2·2, 2·9)	193<br>(159, 236)	2·3<br>(1·9, 2·8)	-9<br>(-25·4, 12·1)
Mauritania	44<br>(36, 55)	4·3<br>(3·5, 5·2)	96<br>(73, 122)	5<br>(3·8, 6·3)	17·1<br>(-7·1, 44·3)
Niger	91<br>(68, 112)	3·1<br>(2·4, 3·8)	304<br>(237, 377)	4·3<br>(3·4, 5·2)	35·4<br>(8·5, 69·7)
Nigeria	1137<br>(856, 1529)	2·5<br>(1·9, 3·3)	2736<br>(1967, 3911)	3·5<br>(2·5, 4·9)	36·9<br>(-1·7, 93)
São Tomé and Príncipe	2<br>(1, 2)	2·3<br>(1·8, 2·8)	4<br>(3, 5)	4·2<br>(3·1, 5·3)	83·7<br>(34, 145·3)
Senegal	115<br>(96, 141)	3·5<br>(2·9, 4·3)	347<br>(281, 446)	4·9<br>(4, 6·3)	40·6<br>(14, 69·8)
Sierra Leone	60<br>(45, 72)	3<br>(2·3, 3·6)	164<br>(132, 200)	4·9<br>(4, 6)	61·4<br>(23·9, 109·9)
Togo	39<br>(33, 46)	3·1<br>(2·6, 3·6)	165<br>(129, 211)	4·8<br>(3·8, 6·1)	54·4<br>(22·4, 92·7)
**Eastern sub-Saharan Africa**	**8050<br>(7274, 8878)**	**10·7<br>(9·8, 11·8)**	**11842<br>(10911, 13029)**	**7·8<br>(7·2, 8·6)**	**-27·4<br>(-34·7, -19)**
Burundi	416<br>(338, 533)	18·7<br>(15·4, 23·9)	355<br>(286, 438)	8·9<br>(7·3, 10·8)	-52·3<br>(-62·8, -39·5)
Comoros	32<br>(25, 41)	15·3<br>(12·1, 20)	35<br>(29, 42)	8·1<br>(6·7, 9·6)	-47·2<br>(-59·2, -32·5)
Djibouti	19<br>(13, 31)	12·8<br>(8·9, 20·1)	44<br>(30, 64)	8·2<br>(5·7, 11·7)	-36·4<br>(-58·2, -7)
Eritrea	182<br>(139, 235)	18·3<br>(14·6, 23·1)	218<br>(177, 263)	9·8<br>(8, 11·6)	-46·8<br>(-58·9, -31·8)
Ethiopia	1812<br>(1497, 2169)	9·8<br>(8·2, 11·5)	1770<br>(1584, 1984)	4·9<br>(4·4, 5·5)	-50·2<br>(-60·4, -37·8)
Kenya	733<br>(575, 973)	8·8<br>(6·9, 11·6)	1624<br>(1337, 2063)	7·9<br>(6·5, 10·2)	-9·8<br>(-17·8, -1·6)
Madagascar	603<br>(509, 725)	11·4<br>(9·6, 13·6)	754<br>(605, 921)	7·3<br>(6, 8·8)	-35·6<br>(-49·2, -19·9)
Malawi	922<br>(573, 1134)	21·7<br>(14·3, 26·2)	1736<br>(1459, 2006)	23<br>(19·4, 26·5)	6<br>(-18·3, 64·8)
Mozambique	426<br>(367, 490)	7·3<br>(6·3, 8·3)	780<br>(653, 940)	7·5<br>(6·3, 8·9)	2·8<br>(-15·3, 26·3)
Rwanda	468<br>(297, 568)	15·7<br>(10, 19)	361<br>(254, 452)	6·6<br>(4·7, 8·2)	-58<br>(-65·9, -47·4)
Somalia	427<br>(263, 617)	16·2<br>(10·9, 22·4)	645<br>(482, 833)	10·5<br>(7·9, 13·4)	-35·5<br>(-54·8, -0·4)
South Sudan	410<br>(264, 614)	17·1<br>(11·6, 24·7)	423<br>(309, 568)	11·5<br>(8·7, 15·3)	-32·4<br>(-53, 1·5)
Tanzania	564<br>(440, 688)	5·5<br>(4·4, 6·6)	941<br>(802, 1103)	4·3<br>(3·6, 5)	-22·9<br>(-37·7, -2·5)
Uganda	823<br>(697, 971)	12·4<br>(10·6, 14·5)	1814<br>(1525, 2138)	13·3<br>(11·3, 15·6)	7·1<br>(-13·8, 32·2)
Zambia	209<br>(170, 241)	7·8<br>(6·5, 8·9)	334<br>(286, 385)	5·8<br>(5, 6·6)	-25·9<br>(-39·3, -8·1)
**Central sub-Saharan Africa**	**2427<br>(2099, 2798)**	**10·4<br>(9, 11·9)**	**3653<br>(3100, 4260)**	**7·3<br>(6·2, 8·5)**	**-30·1<br>(-41·2, -18·5)**
Angola	511<br>(394, 636)	12·4<br>(9·9, 15·3)	888<br>(730, 1055)	8·4<br>(7, 9·8)	-32·6<br>(-48·5, -11·7)
Central African Republic	167<br>(136, 196)	13·6<br>(11·2, 16·1)	216<br>(166, 274)	9·8<br>(7·7, 12·4)	-28·2<br>(-42·6, -9·9)
Congo	161<br>(135, 193)	14·2<br>(12·1, 17)	218<br>(173, 271)	8·9<br>(7·3, 10·9)	-37·6<br>(-49·2, -23·6)

DR Congo	1492<br>(1209, 1811)	9·3<br>(7·7, 11·2)	2198<br>(1718, 2745)	6·6<br>(5·2, 8·2)	-29·4<br>(-45, -12)
Equatorial Guinea	31<br>(25, 38)	15·1<br>(12·1, 18·6)	44<br>(31, 61)	9·8<br>(7·1, 13·4)	-34·9<br>(-53·3, -10·9)
Gabon	65<br>(50, 79)	11·3<br>(8·7, 13·6)	89<br>(68, 107)	8·5<br>(6·6, 10·2)	-24·9<br>(-39·1, -6·5)

Appendix Table 3: Deaths due to oesophageal cancer in 1990 and 2017 for both sexes and percentage change of age-standardised rates per 100 000 by location

	1990		2017		Percentage change in age-standardised rates between 1990 and 2017
	Counts (95% UI)	Rate (95% UI)	Counts (95% UI)	Rate (95% UI)	
**Global**	**311289 (301451, 323235)**	**7·7 (7·5, 8)**	**435959 (424994, 447580)**	**5·5 (5·3, 5·6)**	**-29 (-32, -25·8)**
**High-income North America**	**12354 (12218, 12523)**	**3·5 (3·5, 3·5)**	**21211 (20594, 21780)**	**3·5 (3·4, 3·6)**	**-0·7 (-3·8, 2·3)**
Canada	1206 (1171, 1241)	3·6 (3·5, 3·7)	2388 (2202, 2599)	3·5 (3·3, 3·8)	-2·8 (-10·8, 6·7)
Greenland	5 (4, 5)	14 (12·8, 15·3)	8 (7, 8)	11·4 (10·3, 12·6)	-18·5 (-28·6, -7·2)
USA	11143 (11013, 11301)	3·5 (3·4, 3·5)	18815 (18236, 19357)	3·5 (3·3, 3·6)	-0·7 (-4, 2·5)
**Australasia**	**975 (949, 1002)**	**4 (3·9, 4·2)**	**1855 (1682, 2032)**	**3·8 (3·4, 4·1)**	**-6·6 (-15·5, 3)**
Australia	794 (769, 819)	4 (3·8, 4·1)	1582 (1414, 1757)	3·8 (3·4, 4·3)	-3·4 (-14·1, 8·1)
New Zealand	181 (172, 191)	4·5 (4·2, 4·7)	273 (252, 298)	3·5 (3·2, 3·8)	-21 (-28, -13)
**High-income Asia-Pacific**	**9441 (9314, 9582)**	**4·6 (4·5, 4·7)**	**14765 (14124, 15353)**	**3·3 (3·1, 3·4)**	**-28·6 (-31·9, -25·6)**
Brunei	4 (3, 4)	3·7 (3·4, 4·1)	6 (6, 7)	2·3 (2, 2·5)	-39·5 (-48·3, -29·2)
Japan	7780 (7673, 7897)	4·5 (4·4, 4·5)	12807 (12310, 13311)	3·7 (3·5, 3·8)	-18 (-21·4, -14·8)
Singapore	81 (77, 85)	3·7 (3·5, 3·9)	87 (79, 95)	1·3 (1·2, 1·4)	-65·7 (-69·3, -62)
South Korea	1577 (1515, 1644)	5·1 (4·9, 5·3)	1865 (1653, 2078)	2·2 (1·9, 2·4)	-57·7 (-62·9, -52·4)
**Western Europe**	**24228 (23952, 24556)**	**4·2 (4·1, 4·2)**	**30228 (29195, 31378)**	**3·4 (3·3, 3·5)**	**-18·3 (-21·3, -15·2)**
Andorra	2 (2, 2)	3·5 (3·1, 4·2)	4 (4, 5)	3·1 (2·6, 3·7)	-12 (-29·5, 6·2)
Austria	254 (244, 265)	2·2 (2·1, 2·3)	374 (346, 406)	2·2 (2, 2·4)	1·3 (-7·6, 11·3)
Belgium	523 (503, 543)	3·4 (3·3, 3·5)	833 (765, 904)	3·8 (3·5, 4·1)	11 (1·2, 22·1)
Cyprus	9 (8, 10)	1·1 (1, 1·2)	21 (18, 24)	1·1 (0·9, 1·2)	-1·2 (-17, 19·6)
Denmark	325 (314, 337)	4 (3·9, 4·2)	447 (413, 482)	4 (3·6, 4·3)	-1·6 (-9·7, 7)
Finland	204 (197, 212)	2·8 (2·7, 2·9)	265 (244, 287)	2·2 (2, 2·4)	-22·1 (-28·9, -15)
France	5652 (5479, 5849)	7 (6·8, 7·3)	4365 (4001, 4714)	3·4 (3·1, 3·6)	-52·4 (-56·6, -47·8)
Germany	3851 (3736, 3965)	3·1 (3, 3·2)	5991 (5301, 6704)	3·4 (3, 3·8)	9·7 (-2·8, 24·1)
Greece	259 (248, 271)	1·7 (1·6, 1·7)	279 (257, 306)	1·2 (1·1, 1·3)	-29·7 (-36·4, -22·3)
Iceland	13 (12, 14)	4·4 (4·2, 4·7)	20 (18, 22)	3·7 (3·4, 4)	-16·2 (-24·6, -7·1)
Ireland	283 (272, 295)	6·7 (6·5, 7)	401 (365, 436)	5·5 (4·9, 5·9)	-18·8 (-26·6, -10·9)
Israel	82 (78, 88)	1·7 (1·6, 1·8)	153 (140, 168)	1·3 (1·2, 1·4)	-21·9 (-29·4, -13·4)
Italy	2508 (2432, 2583)	2·8 (2·7, 2·9)	2168 (1999, 2347)	1·5 (1·4, 1·6)	-46·1 (-50·8, -41·3)
Luxembourg	23 (21, 25)	4·2 (3·9, 4·4)	29 (26, 33)	3·1 (2·7, 3·5)	-26·4 (-36·4, -14·8)

Malta	11<br>(11, 12)	2·6<br>(2·4, 2·8)	20<br>(18, 21)	2·2<br>(2, 2·4)	-15·1<br>(-23·8, -4·9)
Netherlands	802<br>(778, 829)	3·9<br>(3·8, 4·1)	2113<br>(1969, 2274)	6·3<br>(5·8, 6·7)	58·6<br>(47·1, 71·7)
Norway	160<br>(157, 164)	2·4<br>(2·3, 2·4)	198<br>(188, 208)	2·1<br>(2, 2·2)	-10·8<br>(-15·6, -5·8)
Portugal	607<br>(584, 631)	4·4<br>(4·2, 4·6)	650<br>(594, 715)	3<br>(2·7, 3·3)	-31·5<br>(-38, -23·9)
Spain	1820<br>(1757, 1879)	3·4<br>(3·2, 3·5)	2058<br>(1899, 2228)	2·3<br>(2·1, 2·5)	-32·2<br>(-38, -25·6)
Sweden	386<br>(373, 399)	2·5<br>(2·4, 2·5)	517<br>(487, 552)	2·5<br>(2·3, 2·6)	-0·3<br>(-7·1, 7)
Switzerland	401<br>(387, 415)	3·8<br>(3·7, 4)	485<br>(445, 530)	2·8<br>(2·6, 3·1)	-25·6<br>(-32·2, -17·9)
United Kingdom	6027<br>(5951, 6109)	6·4<br>(6·3, 6·5)	8808<br>(8608, 9022)	6·9<br>(6·7, 7)	7·6<br>(4·8, 10·5)
**Southern Latin America**	**3388<br>(3302, 3471)**	**7·3<br>(7·1, 7·5)**	**3584<br>(3328, 3919)**	**4·3<br>(4, 4·7)**	**-41·2<br>(-45·8, -35·2)**
Argentina	2283<br>(2209, 2363)	7<br>(6·8, 7·3)	2373<br>(2128, 2679)	4·4<br>(4, 5)	-37·3<br>(-44·1, -28·3)
Chile	735<br>(707, 763)	7·6<br>(7·3, 7·9)	885<br>(796, 992)	3·8<br>(3·4, 4·3)	-50·2<br>(-55·7, -44)
Uruguay	369<br>(353, 386)	9·2<br>(8·8, 9·6)	327<br>(290, 366)	5·9<br>(5·2, 6·7)	-35·5<br>(-43·3, -26·9)
**Eastern Europe**	**11319<br>(11062, 11571)**	**3·9<br>(3·8, 4)**	**10032<br>(9787, 10333)**	**2·9<br>(2·8, 3)**	**-25·4<br>(-27·3, -23·1)**
Belarus	348<br>(333, 364)	2·6<br>(2·5, 2·7)	429<br>(385, 481)	2·7<br>(2·4, 3)	3·3<br>(-7·6, 16·4)
Estonia	61<br>(58, 65)	2·9<br>(2·7, 3·1)	63<br>(54, 73)	2·5<br>(2·1, 3)	-13·2<br>(-27, 3·1)
Latvia	100<br>(95, 105)	2·7<br>(2·6, 2·8)	124<br>(109, 141)	3·3<br>(2·9, 3·8)	23·5<br>(6·2, 41·9)
Lithuania	129<br>(124, 135)	2·8<br>(2·6, 2·9)	204<br>(188, 224)	3·8<br>(3·5, 4·2)	39·1<br>(26·2, 54·5)
Moldova	104<br>(98, 110)	2·2<br>(2·1, 2·4)	94<br>(86, 103)	1·6<br>(1·5, 1·8)	-27·2<br>(-34·8, -19·5)
Russia	8382<br>(8178, 8580)	4·5<br>(4·4, 4·6)	7093<br>(6942, 7247)	3<br>(2·9, 3·1)	-32·8<br>(-34·5, -31)
Ukraine	2195<br>(2103, 2293)	3<br>(2·8, 3·1)	2024<br>(1870, 2198)	2·7<br>(2·5, 2·9)	-8·8<br>(-16·6, -0·3)
**Central Europe**	**4530<br>(4450, 4612)**	**3<br>(2·9, 3)**	**5645<br>(5431, 5865)**	**2·7<br>(2·6, 2·8)**	**-9<br>(-12·5, -5·2)**
Albania	44<br>(40, 47)	2<br>(1·9, 2·2)	59<br>(48, 73)	1·4<br>(1·2, 1·8)	-30<br>(-44, -12·1)
Bosnia and Herzegovina	107<br>(100, 115)	2·6<br>(2·5, 2·8)	130<br>(116, 145)	2·2<br>(1·9, 2·4)	-17·3<br>(-27·1, -6)
Bulgaria	288<br>(276, 302)	2·2<br>(2·2, 2·3)	250<br>(228, 276)	1·8<br>(1·6, 2)	-19·3<br>(-27·4, -10·1)
Croatia	260<br>(247, 272)	3·9<br>(3·7, 4·1)	217<br>(198, 236)	2·6<br>(2·3, 2·8)	-34·2<br>(-40·3, -27·5)
Czech Republic	422<br>(408, 437)	3·1<br>(3, 3·2)	597<br>(550, 650)	3<br>(2·8, 3·2)	-2·7<br>(-11·4, 6·6)
Hungary	589<br>(565, 614)	4<br>(3·9, 4·2)	634<br>(583, 688)	3·5<br>(3·2, 3·8)	-12·8<br>(-20·4, -4·5)
Macedonia	25<br>(23, 28)	1·3<br>(1·2, 1·5)	44<br>(39, 49)	1·3<br>(1·2, 1·5)	-2·5<br>(-15·2, 10·7)
Montenegro	16<br>(14, 18)	2·4<br>(2·2, 2·7)	25<br>(21, 29)	2·5<br>(2·2, 2·9)	1·8<br>(-13·5, 21·3)
Poland	1686<br>(1633, 1740)	3·8<br>(3·6, 3·9)	2070<br>(1906, 2244)	3<br>(2·8, 3·3)	-19·4<br>(-26·6, -12·1)
Romania	455<br>(435, 477)	1·6<br>(1·5, 1·7)	814<br>(751, 884)	2·4<br>(2·2, 2·6)	50·1<br>(37, 64)

Serbia	314<br>(283, 351)	2·6<br>(2·4, 2·9)	391<br>(355, 433)	2·5<br>(2·3, 2·8)	-3·5<br>(-15·6, 9·8)
Slovakia	222<br>(209, 235)	3·7<br>(3·5, 3·9)	304<br>(271, 345)	3·4<br>(3, 3·8)	-9·5<br>(-20·7, 3·4)
Slovenia	102<br>(96, 107)	4<br>(3·8, 4·2)	109<br>(98, 120)	2·6<br>(2·4, 2·9)	-34·3<br>(-42, -26·9)
**Central Asia**	**6215<br>(6062, 6366)**	**13·3<br>(13, 13·6)**	**4219<br>(4012, 4423)**	**6<br>(5·7, 6·3)**	**-54·9<br>(-57·2, -52·3)**
Armenia	64<br>(59, 69)	2·4<br>(2·2, 2·6)	70<br>(65, 76)	1·7<br>(1·6, 1·8)	-30<br>(-36·8, -22)
Azerbaijan	430<br>(407, 456)	8·5<br>(8, 9)	749<br>(654, 882)	8·6<br>(7·5, 10·1)	2<br>(-12·3, 20·5)
Georgia	104<br>(97, 112)	1·6<br>(1·5, 1·8)	153<br>(140, 166)	2·6<br>(2·4, 2·9)	59·8<br>(42, 78)
Kazakhstan	2412<br>(2329, 2499)	19·1<br>(18·5, 19·8)	1110<br>(1028, 1200)	6·9<br>(6·4, 7·4)	-64·1<br>(-67, -61·3)
Kyrgyzstan	239<br>(225, 256)	7·9<br>(7·4, 8·4)	144<br>(134, 155)	3·6<br>(3·3, 3·9)	-54·1<br>(-58·3, -49·8)
Mongolia	272<br>(228, 321)	28·6<br>(24, 33·7)	353<br>(313, 396)	20·5<br>(18·3, 23)	-28·4<br>(-40·1, -13·8)
Tajikistan	292<br>(276, 308)	10·4<br>(9·8, 11)	261<br>(230, 295)	5·2<br>(4·6, 5·9)	-49·8<br>(-56·1, -42·4)
Turkmenistan	623<br>(602, 644)	32·4<br>(31·3, 33·4)	336<br>(305, 369)	9·1<br>(8·2, 10)	-71·9<br>(-74·7, -69·1)
Uzbekistan	1778<br>(1705, 1850)	15·5<br>(14·9, 16·2)	1043<br>(924, 1169)	5<br>(4·4, 5·6)	-67·9<br>(-71·5, -63·8)
**Central Latin America**	**1796<br>(1766, 1827)**	**2·2<br>(2·2, 2·3)**	**3358<br>(3205, 3497)**	**1·5<br>(1·4, 1·5)**	**-33·4<br>(-36·6, -30·3)**
Colombia	614<br>(592, 634)	3·7<br>(3·6, 3·9)	881<br>(785, 987)	1·6<br>(1·5, 1·8)	-56·1<br>(-61, -50·9)
Costa Rica	48<br>(46, 51)	2·8<br>(2·7, 3)	90<br>(82, 99)	1·9<br>(1·7, 2)	-34·2<br>(-41, -27·1)
El Salvador	39<br>(36, 42)	1·3<br>(1·2, 1·4)	88<br>(75, 103)	1·5<br>(1·3, 1·8)	14·1<br>(-4·7, 35·9)
Guatemala	49<br>(47, 52)	1·4<br>(1·4, 1·5)	177<br>(158, 198)	1·7<br>(1·5, 1·9)	17·5<br>(3·8, 34·1)
Honduras	26<br>(22, 30)	1·2<br>(1·1, 1·4)	89<br>(70, 112)	1·6<br>(1·2, 2)	25<br>(-3·2, 62·9)
Mexico	750<br>(732, 767)	1·8<br>(1·7, 1·8)	1445<br>(1397, 1487)	1·3<br>(1·3, 1·3)	-27·3<br>(-30·1, -24·9)
Nicaragua	17<br>(15, 19)	1·1<br>(1, 1·2)	33<br>(29, 38)	0·8<br>(0·7, 0·9)	-31·5<br>(-42·1, -19)
Panama	24<br>(22, 25)	1·6<br>(1·5, 1·8)	56<br>(51, 62)	1·4<br>(1·3, 1·6)	-12·9<br>(-22·8, -1·2)
Venezuela	229<br>(219, 241)	2·5<br>(2·4, 2·6)	498<br>(426, 578)	1·8<br>(1·6, 2·1)	-25·8<br>(-37·6, -13·8)
**Andean Latin America**	**424<br>(401, 449)**	**2·1<br>(2, 2·3)**	**760<br>(687, 834)**	**1·4<br>(1·3, 1·6)**	**-32·9<br>(-39·8, -25·7)**
Bolivia	99<br>(85, 114)	3·2<br>(2·8, 3·7)	200<br>(158, 252)	2·5<br>(2, 3·1)	-23·9<br>(-41·9, -3·4)
Ecuador	124<br>(118, 130)	2·4<br>(2·3, 2·5)	207<br>(186, 232)	1·5<br>(1·3, 1·6)	-39·1<br>(-46, -31)
Peru	201<br>(183, 221)	1·7<br>(1·6, 1·9)	353<br>(301, 409)	1·2<br>(1, 1·3)	-33<br>(-44·1, -19·5)
**Caribbean**	**1000<br>(959, 1050)**	**3·8<br>(3·7, 4)**	**1736<br>(1598, 1900)**	**3·4<br>(3·1, 3·7)**	**-11·7<br>(-18·4, -4·4)**
Antigua and Barbuda	2<br>(2, 2)	3·3<br>(3·1, 3·6)	3<br>(3, 3)	2·8<br>(2·5, 3)	-15·9<br>(-25·1, -4·9)
The Bahamas	10<br>(9, 11)	6·1<br>(5·4, 6·7)	19<br>(16, 21)	4·9<br>(4·4, 5·5)	-18·9<br>(-31, -3·2)
Barbados	17<br>(16, 18)	5·6<br>(5·2, 6)	20<br>(18, 22)	4·1<br>(3·7, 4·6)	-26·4<br>(-34·6, -16·4)

Belize	2<br>(2, 2)	1·9<br>(1·7, 2·1)	6<br>(5, 7)	2·3<br>(2·1, 2·5)	20·3<br>(5·1, 38·8)
Bermuda	4<br>(4, 5)	6·6<br>(6·1, 7·1)	5<br>(5, 6)	4·2<br>(3·8, 4·7)	-36·1<br>(-43·4, -27·5)
Cuba	384<br>(370, 400)	3·7<br>(3·6, 3·8)	887<br>(776, 1008)	4·7<br>(4·1, 5·4)	28<br>(11·2, 45·8)
Dominica	3<br>(3, 3)	4·1<br>(3·9, 4·4)	4<br>(4, 4)	4·3<br>(3·8, 4·7)	3·4<br>(-9·1, 15·6)
Dominican Republic	60<br>(54, 67)	1·6<br>(1·4, 1·8)	178<br>(151, 207)	2<br>(1·7, 2·3)	22·5<br>(0·2, 48·4)
Grenada	5<br>(4, 5)	6·4<br>(6, 6·9)	9<br>(8, 9)	5·7<br>(5·1, 6·3)	-11·8<br>(-22·2, -1)
Guyana	7<br>(7, 8)	1·9<br>(1·7, 2)	10<br>(9, 12)	1·7<br>(1·5, 1·9)	-8<br>(-20, 6)
Haiti	148<br>(112, 191)	4·7<br>(3·6, 6)	232<br>(171, 319)	3·7<br>(2·7, 5·1)	-21·9<br>(-39, 2·2)
Jamaica	57<br>(53, 62)	3·1<br>(2·9, 3·4)	91<br>(77, 108)	3·2<br>(2·7, 3·8)	2·8<br>(-14·8, 23·3)
Puerto Rico	234<br>(224, 245)	6·3<br>(6, 6·6)	157<br>(143, 172)	2·2<br>(2, 2·4)	-64·7<br>(-68·2, -60·9)
Saint Lucia	4<br>(4, 5)	4·9<br>(4·6, 5·3)	8<br>(7, 9)	3·9<br>(3·5, 4·3)	-21·5<br>(-30·7, -11·2)
Saint Vincent and the Grenadines	2<br>(1, 2)	2·1<br>(2, 2·3)	3<br>(3, 4)	2·4<br>(2·1, 2·7)	12·6<br>(-2·5, 30·3)
Suriname	4<br>(3, 4)	1·4<br>(1·3, 1·5)	8<br>(7, 9)	1·4<br>(1·2, 1·6)	-0·8<br>(-15·1, 15·6)
Trinidad and Tobago	21<br>(19, 22)	2·4<br>(2·3, 2·6)	26<br>(21, 32)	1·5<br>(1·2, 1·8)	-40·1<br>(-51·5, -25·4)
Virgin Islands	3<br>(3, 3)	3·6<br>(3·2, 4)	8<br>(6, 9)	4<br>(3·3, 4·6)	12·4<br>(-10, 32·6)
**Tropical Latin America**	**5426**<br>**(5325, 5537)**	**5·9**<br>**(5·8, 6·1)**	**11256**<br>**(10993, 11508)**	**4·8**<br>**(4·7, 4·9)**	**-19·1**<br>**(-21·4, -16·8)**
Brazil	5352<br>(5251, 5464)	6<br>(5·9, 6·1)	11065<br>(10818, 11333)	4·8<br>(4·7, 5)	-19·4<br>(-21·8, -17·1)
Paraguay	73<br>(66, 80)	3·5<br>(3·1, 3·8)	190<br>(157, 231)	3·7<br>(3·1, 4·5)	6·8<br>(-13·2, 33·3)
**East Asia**	**173619**<br>**(165927, 183613)**	**20**<br>**(19·1, 21·2)**	**221066**<br>**(210944, 231096)**	**11·1**<br>**(10·6, 11·6)**	**-44·6**<br>**(-48·9, -40·6)**
China	168455<br>(160933, 178231)	20·5<br>(19·6, 21·7)	212586<br>(202673, 222654)	11·2<br>(10·7, 11·8)	-45·2<br>(-49·6, -41·1)
North Korea	1456<br>(1168, 1773)	9<br>(7·3, 11)	2632<br>(1990, 3269)	8·4<br>(6·4, 10·5)	-7<br>(-27·6, 17·1)
Taiwan (Province of China)	816<br>(789, 845)	5·1<br>(5, 5·3)	2287<br>(2120, 2472)	6<br>(5·5, 6·4)	16·3<br>(6·7, 25·8)
**Southeast Asia**	**7930**<br>**(7103, 8626)**	**3·2**<br>**(2·9, 3·5)**	**14818**<br>**(13909, 15905)**	**2·6**<br>**(2·5, 2·8)**	**-19·3**<br>**(-26·9, -7·8)**
Cambodia	211<br>(177, 246)	4·7<br>(3·9, 5·4)	292<br>(242, 358)	2·7<br>(2·2, 3·3)	-42·6<br>(-53·7, -28·8)
Indonesia	911<br>(821, 1041)	1<br>(0·9, 1·1)	1717<br>(1592, 1843)	0·8<br>(0·8, 0·9)	-12·6<br>(-25·9, 0)
Laos	100<br>(81, 120)	4·8<br>(3·9, 5·7)	106<br>(81, 134)	2·6<br>(2, 3·3)	-46<br>(-57·8, -32)
Malaysia	263<br>(241, 289)	3·1<br>(2·8, 3·4)	656<br>(583, 750)	2·8<br>(2·5, 3·2)	-10·2<br>(-24, 8·2)
Maldives	3<br>(2, 4)	3·7<br>(2·9, 4·6)	4<br>(4, 4)	1·5<br>(1·3, 1·7)	-59·8<br>(-68·4, -44·5)
Mauritius	22<br>(21, 24)	3·2<br>(3, 3·4)	37<br>(33, 40)	2·3<br>(2·1, 2·5)	-28·4<br>(-36·1, -20·1)
Myanmar	2849<br>(2286, 3454)	12·9<br>(10·4, 15·5)	4051<br>(3525, 4689)	9·5<br>(8·3, 11)	-26<br>(-42·2, -5·4)
Philippines	424<br>(397, 452)	1·5<br>(1·4, 1·6)	883<br>(769, 1006)	1·3<br>(1·1, 1·5)	-12<br>(-23·6, 1·5)

Sri Lanka	505<br>(459, 552)	4·7<br>(4·3, 5·1)	953<br>(798, 1129)	3·9<br>(3·3, 4·6)	-16·3<br>(-32·2, 2·2)
Seychelles	3<br>(3, 4)	6·1<br>(5·5, 6·8)	6<br>(5, 6)	5·2<br>(4·7, 5·7)	-15·9<br>(-27·9, -3·5)
Thailand	1094<br>(1002, 1188)	3·2<br>(2·9, 3·5)	2868<br>(2526, 3255)	2·9<br>(2·5, 3·3)	-10·2<br>(-23·1, 5)
East Timor	9<br>(7, 11)	3·2<br>(2·6, 3·9)	17<br>(13, 22)	2·2<br>(1·7, 2·9)	-30·9<br>(-46, -13·4)
Vietnam	1524<br>(1256, 1808)	3·8<br>(3·2, 4·5)	3208<br>(2709, 3897)	3·5<br>(3, 4·2)	-8·9<br>(-29·5, 21·9)
**Oceania**	**66<br>(56, 77)**	**2·3<br>(2, 2·7)**	**140<br>(119, 165)**	**2·3<br>(2, 2·6)**	**-1·3<br>(-12, 10·8)**
American Samoa	0<br>(0, 0)	1·3<br>(1·1, 1·4)	1<br>(0, 1)	1·4<br>(1·2, 1·6)	12·4<br>(-7·2, 35·1)
Federated States of Micronesia	1<br>(1, 1)	2·5<br>(2·1, 3)	1<br>(1, 2)	2·2<br>(1·9, 2·6)	-11·5<br>(-28·9, 9·9)
Fiji	6<br>(6, 7)	2<br>(1·7, 2·3)	17<br>(14, 19)	2·6<br>(2·2, 2·9)	29·7<br>(5·1, 60·1)
Guam	1<br>(1, 2)	2<br>(1·8, 2·2)	4<br>(4, 5)	2·5<br>(2·3, 2·9)	26·9<br>(8·2, 48·2)
Kiribati	1<br>(1, 1)	3·8<br>(3·4, 4·3)	3<br>(2, 3)	4·5<br>(3·8, 5·2)	15·9<br>(-3·9, 40·8)
Marshall Islands	1<br>(0, 1)	3·4<br>(2·9, 4)	1<br>(1, 1)	3·4<br>(2·9, 4·1)	1·3<br>(-15·5, 21·3)
Northern Mariana Islands	0<br>(0, 0)	1·5<br>(1·3, 1·9)	1<br>(1, 1)	1·7<br>(1·5, 2)	12·9<br>(-9·5, 38·8)
Papua New Guinea	43<br>(35, 52)	2·3<br>(1·9, 2·8)	91<br>(72, 113)	2·2<br>(1·7, 2·6)	-7·2<br>(-22·8, 11·2)
Samoa	1<br>(1, 2)	1·6<br>(1·3, 2)	2<br>(2, 3)	1·6<br>(1·3, 2·1)	2·7<br>(-17·1, 25·3)
Solomon Islands	3<br>(3, 4)	2·4<br>(1·9, 3)	7<br>(5, 8)	2·3<br>(1·8, 2·8)	-7·2<br>(-23·5, 11·4)
Tonga	1<br>(1, 1)	2·4<br>(2, 2·8)	2<br>(2, 2)	2·5<br>(2·2, 3)	4·7<br>(-14·2, 30·5)
Vanuatu	1<br>(1, 2)	2·2<br>(1·6, 2·8)	3<br>(3, 4)	2·2<br>(1·7, 2·8)	1·6<br>(-18·3, 26·8)
**North Africa and Middle East**	**4760<br>(4273, 5242)**	**2·8<br>(2·5, 3·1)**	**9193<br>(8552, 9938)**	**2·3<br>(2·1, 2·5)**	**-18·4<br>(-24·5, -11·1)**
Afghanistan	693<br>(425, 912)	9·7<br>(6·1, 12·7)	862<br>(667, 1078)	8·3<br>(6·5, 10·6)	-14·8<br>(-32·8, 32·3)
Algeria	137<br>(122, 153)	1·1<br>(1, 1·3)	319<br>(285, 356)	1<br>(0·9, 1·1)	-11·5<br>(-25, 2·2)
Bahrain	6<br>(6, 7)	3·9<br>(3·5, 4·2)	10<br>(9, 12)	1·3<br>(1·1, 1·4)	-67·4<br>(-72·2, -62)
Egypt	431<br>(392, 476)	1·6<br>(1·4, 1·7)	1045<br>(921, 1191)	1·8<br>(1·6, 2·1)	16·5<br>(-0·7, 35·5)
Iran	1264<br>(1174, 1379)	5·3<br>(4·9, 5·7)	2816<br>(2708, 2983)	4·4<br>(4·2, 4·6)	-17·4<br>(-24·1, -10·1)
Iraq	118<br>(101, 137)	1·5<br>(1·3, 1·8)	154<br>(140, 168)	0·7<br>(0·6, 0·7)	-56·1<br>(-63·4, -46·8)
Jordan	17<br>(15, 20)	1·3<br>(1·1, 1·4)	56<br>(48, 65)	1·1<br>(0·9, 1·2)	-16<br>(-31·3, 4·2)
Kuwait	9<br>(9, 10)	1·6<br>(1·5, 1·7)	24<br>(22, 27)	1·1<br>(1, 1·2)	-31·9<br>(-40·1, -22·6)
Lebanon	31<br>(25, 36)	1·4<br>(1·2, 1·7)	68<br>(60, 77)	1·2<br>(1·1, 1·3)	-17·3<br>(-32·3, 8)
Libya	26<br>(22, 31)	1·4<br>(1·2, 1·7)	76<br>(62, 94)	1·7<br>(1·4, 2·1)	24·5<br>(-5·3, 60·7)
Morocco	196<br>(171, 224)	1·4<br>(1·2, 1·6)	404<br>(335, 476)	1·3<br>(1·1, 1·5)	-4·8<br>(-24, 19·9)
Palestine	12<br>(10, 14)	1·3<br>(1·1, 1·6)	23<br>(21, 25)	1<br>(0·9, 1·1)	-25·4<br>(-39·7, -6·9)

Oman	18<br>(15, 23)	2·7<br>(2·2, 3·4)	43<br>(35, 52)	2·3<br>(1·9, 2·8)	-15<br>(-35, 9·5)
Qatar	4<br>(4, 5)	5·3<br>(4·6, 6·1)	15<br>(12, 18)	2·4<br>(2, 2·9)	-54·4<br>(-64·3, -41·9)
Saudi Arabia	130<br>(107, 159)	2·2<br>(1·8, 2·7)	288<br>(249, 341)	2·1<br>(1·8, 2·4)	-5·9<br>(-24·3, 19·7)
Sudan	469<br>(339, 658)	5·1<br>(3·7, 7·3)	806<br>(563, 1095)	4·7<br>(3·3, 6·5)	-7·6<br>(-28·3, 17·7)
Syria	53<br>(46, 62)	1<br>(0·9, 1·2)	125<br>(101, 153)	1<br>(0·8, 1·2)	-0·8<br>(-23·9, 28·1)
Tunisia	42<br>(38, 47)	0·9<br>(0·8, 1)	105<br>(84, 128)	0·9<br>(0·7, 1·1)	-0·3<br>(-21·8, 24·8)
Turkey	826<br>(744, 921)	2·2<br>(2, 2·5)	1134<br>(1029, 1254)	1·3<br>(1·2, 1·4)	-42·5<br>(-50·1, -33·6)
United Arab Emirates	27<br>(19, 37)	6·5<br>(4·6, 8·9)	315<br>(231, 432)	8·9<br>(6·6, 12·2)	36·7<br>(2·7, 86·9)
Yemen	244<br>(163, 347)	5<br>(3·4, 7·4)	496<br>(367, 659)	4·1<br>(3·1, 5·5)	-18·1<br>(-41·6, 18·3)
**South Asia**	**27043<br>(25433, 28572)**	**4·7<br>(4·4, 5)**	**53633<br>(50604, 56781)**	**4·1<br>(3·9, 4·4)**	**-11·6<br>(-17·9, -3·5)**
Bangladesh	2072<br>(1829, 2421)	4·3<br>(3·8, 5)	3093<br>(2664, 3553)	2·8<br>(2·4, 3·2)	-35·7<br>(-47·9, -21·9)
Bhutan	20<br>(16, 24)	7·8<br>(6·4, 9·5)	27<br>(21, 33)	4·6<br>(3·7, 5·7)	-41·4<br>(-56·3, -21·8)
India	20156<br>(18926, 21299)	4·3<br>(4, 4·5)	40584<br>(38137, 42936)	3·8<br>(3·6, 4·1)	-10·8<br>(-16·7, -3·5)
Nepal	613<br>(517, 739)	6·4<br>(5·4, 7·7)	1094<br>(897, 1299)	5·2<br>(4·3, 6·2)	-18·2<br>(-33·2, -1·1)
Pakistan	4183<br>(3680, 4781)	7·4<br>(6·5, 8·4)	8834<br>(7258, 10593)	8·2<br>(6·8, 9·8)	11·5<br>(-10·7, 38·5)
**Southern sub-Saharan Africa**	**3549<br>(3271, 3849)**	**12·7<br>(11·7, 13·8)**	**5639<br>(5349, 5903)**	**10·5<br>(10, 11)**	**-16·9<br>(-24·1, -8·4)**
Botswana	71<br>(57, 87)	12·3<br>(10, 14·8)	103<br>(86, 123)	8·2<br>(6·9, 9·7)	-33·4<br>(-46·9, -16)
Lesotho	137<br>(116, 159)	13·7<br>(11·7, 15·9)	170<br>(143, 199)	14·4<br>(12·1, 16·9)	5<br>(-14·6, 28·6)
Namibia	27<br>(24, 32)	3·7<br>(3·2, 4·2)	42<br>(36, 49)	3<br>(2·6, 3·4)	-19·2<br>(-33·5, -2·6)
South Africa	2706<br>(2471, 2959)	12·6<br>(11·5, 13·8)	4278<br>(4089, 4479)	10<br>(9·6, 10·5)	-20·5<br>(-27·9, -12·1)
Swaziland	54<br>(44, 66)	18·4<br>(14·9, 22·1)	87<br>(68, 107)	15·5<br>(12·2, 18·8)	-15·6<br>(-34·6, 6·2)
Zimbabwe	554<br>(474, 637)	13·5<br>(11·6, 15·4)	959<br>(811, 1128)	14·4<br>(12·4, 17)	7·1<br>(-14·6, 34·1)
**Western sub-Saharan Africa**	**2661<br>(2326, 3108)**	**3·1<br>(2·7, 3·6)**	**7050<br>(6148, 8363)**	**4·2<br>(3·7, 5)**	**37·2<br>(17·1, 60·3)**
Benin	75<br>(65, 86)	3·8<br>(3·3, 4·3)	289<br>(230, 363)	6·6<br>(5·3, 8·1)	75·1<br>(39·6, 122·8)
Burkina Faso	258<br>(205, 302)	6·6<br>(5·3, 7·6)	524<br>(444, 615)	6·8<br>(5·8, 7·9)	2·7<br>(-17·7, 32)
Cameroon	202<br>(156, 237)	4·6<br>(3·5, 5·4)	769<br>(593, 951)	7·1<br>(5·6, 8·7)	54<br>(20, 94·5)
Cape Verde	19<br>(17, 21)	8·2<br>(7·4, 9)	46<br>(40, 52)	10·6<br>(9·3, 12)	28·4<br>(8·3, 50)
Chad	85<br>(71, 100)	3<br>(2·5, 3·5)	283<br>(224, 355)	5·5<br>(4·4, 6·9)	84·9<br>(47·6, 128·4)
Côte d'Ivoire	41<br>(35, 48)	1·1<br>(0·9, 1·3)	124<br>(100, 152)	1·3<br>(1·1, 1·6)	21·9<br>(-3·4, 52·1)
The Gambia	7<br>(6, 8)	1·9<br>(1·6, 2·3)	20<br>(16, 23)	2·2<br>(1·8, 2·6)	13·2<br>(-10·1, 42·6)
Ghana	213<br>(179, 254)	3·6<br>(3, 4·2)	577<br>(496, 663)	4<br>(3·5, 4·6)	13·3<br>(-9·2, 42)

Guinea	61 (54, 69)	1·8 (1·6, 2)	126 (103, 152)	2·4 (2, 2·9)	31·8 (6, 65·1)
Guinea-Bissau	22 (18, 26)	5·4 (4·6, 6·3)	55 (45, 69)	8·3 (6·8, 10·2)	53·1 (21·4, 95·5)
Liberia	45 (38, 53)	4 (3·4, 4·6)	109 (87, 135)	6 (4·9, 7·4)	52·8 (22·8, 93·8)
Mali	106 (93, 121)	2·6 (2·3, 3)	194 (160, 236)	2·4 (2, 2·9)	-8·1 (-24, 13·8)
Mauritania	44 (36, 55)	4·4 (3·6, 5·3)	99 (76, 124)	5·3 (4·1, 6·6)	20·5 (-4, 48·2)
Niger	91 (68, 111)	3·3 (2·5, 4)	306 (239, 379)	4·5 (3·6, 5·5)	37·2 (10·5, 70·5)
Nigeria	1173 (889, 1558)	2·7 (2·1, 3·5)	2836 (2058, 4024)	3·8 (2·8, 5·3)	39·5 (1·4, 94·4)
São Tomé and Príncipe	2 (1, 2)	2·4 (1·9, 2·9)	4 (3, 5)	4·4 (3·2, 5·6)	85·8 (37, 147·3)
Senegal	117 (98, 143)	3·7 (3·1, 4·5)	356 (292, 455)	5·2 (4·3, 6·6)	42·2 (16·2, 70·6)
Sierra Leone	62 (46, 74)	3·2 (2·4, 3·8)	168 (136, 205)	5·2 (4·3, 6·3)	63·1 (25·7, 112·5)
Togo	39 (33, 46)	3·2 (2·8, 3·8)	165 (130, 211)	5 (4, 6·4)	55·2 (24, 93·4)
**Eastern sub-Saharan Africa**	**8143 (7392, 8937)**	**11·4 (10·4, 12·5)**	**12100 (11150, 13251)**	**8·4 (7·7, 9·2)**	**-26·8 (-34, -18·7)**
Burundi	418 (342, 536)	19·6 (16·2, 25·2)	360 (292, 445)	9·6 (7·8, 11·6)	-51·2 (-61·7, -38·6)
Comoros	32 (25, 42)	16·1 (12·8, 21)	37 (30, 44)	8·6 (7·2, 10·3)	-46·1 (-58·2, -31·9)
Djibouti	19 (13, 30)	13·6 (9·5, 21·2)	45 (31, 64)	8·8 (6·1, 12·6)	-35·8 (-57·3, -6·8)
Eritrea	180 (139, 230)	19·2 (15·4, 24·2)	218 (177, 261)	10·4 (8·5, 12·4)	-45·8 (-58·1, -31)
Ethiopia	1813 (1510, 2163)	10·5 (8·9, 12·3)	1840 (1648, 2067)	5·3 (4·8, 6)	-49·3 (-59·5, -37·2)
Kenya	743 (584, 987)	9·2 (7·3, 12·2)	1644 (1351, 2118)	8·4 (6·9, 10·9)	-8·8 (-16·5, -1)
Madagascar	609 (514, 729)	11·9 (10·1, 14·3)	760 (612, 928)	7·8 (6·4, 9·4)	-34·6 (-48·3, -18·7)
Malawi	928 (594, 1132)	22·6 (15·3, 27·2)	1758 (1478, 2028)	23·8 (20·1, 27·5)	5·4 (-17·9, 59·2)
Mozambique	440 (380, 505)	7·9 (6·9, 9)	796 (669, 950)	8 (6·8, 9·5)	1·7 (-16·3, 24·4)
Rwanda	472 (299, 571)	16·6 (10·5, 19·9)	373 (264, 464)	7·2 (5·1, 8·9)	-56·7 (-64·9, -46)
Somalia	423 (266, 606)	17·1 (11·7, 23·5)	651 (485, 843)	11·2 (8·5, 14·2)	-34·8 (-53·9, -0·1)
South Sudan	418 (275, 619)	18·1 (12·5, 26·2)	426 (313, 573)	12·3 (9·3, 16·1)	-32·2 (-52·4, 0·8)
Tanzania	588 (466, 713)	6·1 (5, 7·2)	993 (846, 1167)	4·7 (4, 5·5)	-22·7 (-37·2, -4)
Uganda	839 (712, 987)	13·2 (11·3, 15·3)	1844 (1558, 2162)	14·1 (12, 16·4)	7·2 (-13·2, 31·7)
Zambia	216 (178, 248)	8·5 (7·2, 9·7)	346 (297, 398)	6·3 (5·5, 7·2)	-25·7 (-38·6, -8·1)
**Central sub-Saharan Africa**	**2423 (2096, 2793)**	**10·9 (9·5, 12·5)**	**3670 (3113, 4279)**	**7·7 (6·5, 9)**	**-29·6 (-40·6, -18·1)**
Angola	504 (391, 626)	12·9 (10·4, 15·9)	887 (730, 1049)	8·8 (7·4, 10·3)	-31·7 (-47·4, -11·6)
Central African Republic	165 (134, 194)	14·2 (11·7, 16·7)	214 (165, 270)	10·2 (8, 12·8)	-27·7 (-41·9, -9·7)
Congo	161 (136, 193)	14·8 (12·7, 17·6)	219 (176, 271)	9·4 (7·8, 11·5)	-36·3 (-47·9, -22·5)

DR Congo	1495<br>(1212, 1814)	9·8<br>(8·1, 11·8)	2215<br>(1734, 2768)	6·9<br>(5·5, 8·7)	-29·2<br>(-44·2, -12·1)
Equatorial Guinea	31<br>(25, 38)	15·6<br>(12·7, 19·1)	45<br>(32, 62)	10·5<br>(7·7, 14·2)	-32·9<br>(-51·3, -8·6)
Gabon	67<br>(51, 80)	11·8<br>(9·2, 14·2)	91<br>(70, 109)	9<br>(7·1, 10·7)	-24·4<br>(-38·4, -6·1)

Appendix Table 4: DALYs due to oesophageal cancer in 1990 and 2017 for both sexes and percentage change of age-standardised rates per 100 000 by location

	1990		2017		Percentage change in age-standardised rates between 1990 and 2017
	Counts (95% UI)	Rate (95% UI)	Counts (95% UI)	Rate (95% UI)	
**Global**	**7677078 (7422100, 7970949)**	**179·9 (174·1, 186·8)**	**9777771 (9532791, 10031497)**	**119·9 (116·9, 123)**	**-33·4 (-36·1, -30·4)**
**High-income North America**	**278702 (275146, 282753)**	**83·3 (82·2, 84·5)**	**454008 (439322, 466732)**	**78·4 (75·9, 80·7)**	**-5·8 (-9·2, -2·6)**
Canada	25910 (25123, 26718)	79·7 (77·2, 82·2)	48328 (44103, 52897)	76·9 (70·2, 83·9)	-3·5 (-12·6, 6·3)
Greenland	121 (110, 133)	314·3 (286·4, 343·9)	182 (163, 204)	246·5 (220·4, 273·2)	-21·6 (-31·7, -9·9)
USA	252664 (249271, 256404)	83·7 (82·6, 85)	405490 (391034, 417965)	78·6 (75·8, 81)	-6·1 (-9·6, -2·8)
**Australasia**	**20399 (19824, 21025)**	**85·7 (83·3, 88·3)**	**35360 (31942, 39040)**	**78·1 (70·5, 86·4)**	**-8·9 (-18·3, 1·4)**
Australia	16742 (16216, 17328)	84·4 (81·7, 87·4)	30171 (26821, 33778)	79·3 (70·5, 89)	-6·1 (-17·1, 6·1)
New Zealand	3657 (3470, 3836)	91·8 (87·2, 96·3)	5188 (4746, 5664)	71·7 (65·7, 78·2)	-21·9 (-29, -14)
**High-income Asia-Pacific**	**216941 (213511, 220720)**	**102 (100·4, 103·7)**	**268467 (255959, 279731)**	**68·3 (65, 71·3)**	**-33·1 (-36·5, -30)**
Brunei	88 (79, 98)	79·9 (71·8, 89·2)	167 (147, 188)	49 (43·2, 55·3)	-38·7 (-48·2, -28·3)
Japan	172127 (169542, 174893)	96·9 (95·4, 98·4)	227447 (218017, 236700)	77·8 (74·5, 81·1)	-19·7 (-23·3, -16·3)
Singapore	1856 (1750, 1958)	77·3 (72·9, 81·4)	1866 (1680, 2059)	26·3 (23·8, 29)	-65·9 (-69·7, -62·2)
South Korea	42870 (41041, 44820)	124·5 (119·4, 129·8)	38988 (34415, 43822)	44·6 (39·3, 50)	-64·2 (-68·8, -59·6)
**Western Europe**	**535431 (528202, 543648)**	**98·2 (96·8, 99·8)**	**592534 (569919, 617019)**	**75·7 (72·7, 78·8)**	**-22·9 (-26·1, -19·6)**
Andorra	45 (39, 54)	75 (64·2, 89·7)	85 (71, 102)	64·6 (54, 77·5)	-13·8 (-32·8, 6·1)
Austria	5824 (5583, 6079)	53·8 (51·5, 56·4)	8037 (7357, 8761)	51·9 (47·4, 56·9)	-3·5 (-12·7, 6·7)
Belgium	11352 (10892, 11843)	78·8 (75·5, 82·3)	16940 (15468, 18452)	85·8 (78·3, 93·5)	8·9 (-1·5, 20·1)
Cyprus	204 (180, 229)	24·2 (21·3, 27·1)	425 (370, 491)	23 (20·1, 26·3)	-5·1 (-20·8, 15·6)
Denmark	6961 (6698, 7230)	93·2 (89·6, 97)	8866 (8124, 9600)	84·9 (77·9, 92)	-9 (-17·1, -0·4)
Finland	4088 (3935, 4258)	57·9 (55·7, 60·3)	5029 (4617, 5481)	46·4 (42·5, 50·7)	-19·8 (-27·2, -11·6)
France	133841 (129264, 138641)	179 (172·7, 185·4)	88073 (79794, 96043)	77·5 (70·2, 84·8)	-56·7 (-60·9, -52·4)
Germany	95228 (91957, 98523)	81·3 (78·5, 84·2)	127210 (112423, 144197)	80·8 (71, 92)	-0·6 (-12·8, 14·1)
Greece	5089 (4855, 5335)	33·5 (32, 35·1)	5077 (4644, 5599)	25·8 (23·4, 28·6)	-23·1 (-30·9, -13·9)
Iceland	275 (257, 295)	99·9 (93·2, 107·5)	405 (371, 441)	81·1 (74·4, 88·4)	-18·8 (-27·7, -8·8)
Ireland	5611 (5391, 5850)	136·8 (131·4, 143)	7662 (6888, 8398)	108·5 (97·5, 119·1)	-20·7 (-28·5, -11·9)
Israel	1539 (1457, 1638)	31·3 (29·6, 33·2)	2678 (2433, 2957)	24·9 (22·7, 27·5)	-20·3 (-28·5, -11·1)
Italy	54658 (52802, 56422)	63·8 (61·6, 65·8)	39353 (36152, 43005)	32·2 (29·6, 35·4)	-49·4 (-54, -44·4)
Luxembourg	531 (492, 569)	99·6 (92·4, 106·9)	622 (543, 712)	68·3 (59·3, 78·2)	-31·4 (-41·3, -19·5)

Malta	252<br>(233, 273)	57·7<br>(53·3, 62·3)	393<br>(358, 434)	49<br>(44·5, 53·8)	-15<br>(-24·3, -4·2)
Netherlands	16541<br>(16006, 17136)	85·1<br>(82·3, 88·3)	41376<br>(38226, 44710)	131·9<br>(121·9, 142·4)	55<br>(42, 68·6)
Norway	3209<br>(3135, 3283)	52·1<br>(50·9, 53·3)	3870<br>(3679, 4070)	44·7<br>(42·4, 47)	-14·2<br>(-19·1, -9·5)
Portugal	13444<br>(12900, 14065)	98·8<br>(94·7, 103·5)	14561<br>(13157, 16177)	76·7<br>(68·9, 85·6)	-22·4<br>(-30·7, -12·1)
Spain	43734<br>(42147, 45378)	84·8<br>(81·6, 88·1)	43427<br>(39752, 47362)	53·8<br>(49·1, 58·8)	-36·5<br>(-42·4, -29·9)
Sweden	7288<br>(7046, 7545)	52<br>(50·2, 53·8)	9437<br>(8851, 10079)	50·7<br>(47·5, 54·3)	-2·4<br>(-9·2, 4·9)
Switzerland	8654<br>(8320, 8974)	88·7<br>(85·2, 92·2)	9267<br>(8460, 10142)	60<br>(54·7, 65·8)	-32·3<br>(-38·9, -24·5)
United Kingdom	116545<br>(115075, 118202)	132·9<br>(131·3, 134·8)	159127<br>(155383, 163054)	138·6<br>(135·4, 142)	4·2<br>(1·5, 7·2)
**Southern Latin America**	**73691<br>(71697, 75810)**	**154·5<br>(150·4, 158·9)**	**70110<br>(64358, 77363)**	**86·9<br>(79·7, 96)**	**-43·8<br>(-48·6, -37·3)**
Argentina	51070<br>(49318, 52995)	153·6<br>(148·4, 159·2)	48758<br>(43210, 55673)	93·9<br>(83, 107·3)	-38·9<br>(-46·2, -29·6)
Chile	14797<br>(14183, 15371)	144·2<br>(138·2, 149·7)	15040<br>(13457, 16974)	64·6<br>(57·9, 72·9)	-55·2<br>(-60·2, -49·3)
Uruguay	7821<br>(7457, 8199)	200·7<br>(191·4, 210·4)	6309<br>(5524, 7130)	127·3<br>(110·7, 144·4)	-36·6<br>(-45·1, -27)
**Eastern Europe**	**285809<br>(278415, 292687)**	**97·4<br>(94·8, 99·7)**	**253360<br>(246462, 261310)**	**75·7<br>(73·7, 78·2)**	**-22·3<br>(-24·6, -19·9)**
Belarus	9154<br>(8730, 9584)	68·6<br>(65·4, 72)	10997<br>(9781, 12480)	70·1<br>(62·4, 79·2)	2·2<br>(-9·6, 16·1)
Estonia	1578<br>(1488, 1679)	75·8<br>(71·5, 80·6)	1423<br>(1200, 1681)	62·8<br>(52·6, 74·5)	-17·2<br>(-31·4, -0·8)
Latvia	2553<br>(2410, 2696)	69·3<br>(65·5, 73·3)	2932<br>(2527, 3372)	85·3<br>(72·9, 98·1)	23<br>(4·9, 42·9)
Lithuania	3380<br>(3207, 3552)	72·8<br>(69·1, 76·4)	4967<br>(4515, 5481)	100·9<br>(91·6, 111·5)	38·7<br>(24·6, 54·6)
Moldova	2893<br>(2719, 3095)	60·9<br>(57·2, 65·1)	2530<br>(2304, 2790)	44·4<br>(40·5, 48·7)	-27<br>(-34·9, -18·7)
Russia	207747<br>(202125, 213218)	108·7<br>(105·6, 111·6)	175425<br>(171521, 179375)	76·2<br>(74·4, 77·9)	-29·9<br>(-31·8, -28·1)
Ukraine	58504<br>(55788, 61361)	80·3<br>(76·7, 84·3)	55085<br>(50580, 60112)	76·9<br>(70·6, 84·2)	-4·2<br>(-13·4, 5·1)
**Central Europe**	**116569<br>(114208, 118969)**	**76·8<br>(75·3, 78·4)**	**133458<br>(128234, 139090)**	**68·7<br>(66, 71·6)**	**-10·6<br>(-14·4, -6·6)**
Albania	1121<br>(1026, 1214)	47·9<br>(43·9, 51·8)	1285<br>(1036, 1606)	31·8<br>(25·7, 39·8)	-33·5<br>(-47·9, -16·5)
Bosnia and Herzegovina	2762<br>(2565, 2970)	60·3<br>(56·3, 64·6)	2975<br>(2634, 3348)	50·1<br>(44·4, 56·3)	-16·8<br>(-27·6, -4·9)
Bulgaria	7354<br>(7023, 7694)	57·9<br>(55·2, 60·5)	6016<br>(5423, 6619)	48<br>(43·1, 52·9)	-17<br>(-25·9, -7·1)
Croatia	6741<br>(6387, 7109)	100·1<br>(95, 105·3)	4837<br>(4393, 5250)	61·7<br>(56, 67)	-38·4<br>(-44·7, -31·8)
Czech Republic	10353<br>(9972, 10745)	77·4<br>(74·4, 80·3)	13526<br>(12406, 14783)	72·8<br>(66·6, 79·8)	-6<br>(-14·8, 3·8)
Hungary	16618<br>(15883, 17408)	118<br>(112·6, 123·9)	15629<br>(14306, 17093)	93·3<br>(85·4, 102·1)	-20·9<br>(-28·5, -12·9)
Macedonia	670<br>(614, 733)	33<br>(30·4, 36)	1092<br>(971, 1226)	33·2<br>(29·6, 37·3)	0·5<br>(-13·5, 14·7)
Montenegro	414<br>(365, 468)	61·8<br>(54·6, 69·8)	587<br>(507, 686)	59·9<br>(51·6, 69·9)	-3·1<br>(-19·4, 17·2)
Poland	41441<br>(40032, 42911)	92·2<br>(89, 95·4)	47531<br>(43403, 51778)	73·4<br>(67·1, 80)	-20·4<br>(-27·8, -12·5)
Romania	12168<br>(11633, 12791)	41·9<br>(40·1, 44)	20837<br>(19138, 22702)	65·6<br>(60, 71·7)	56·4<br>(42·2, 72·8)

Serbia	8387<br>(7532, 9373)	67·5<br>(60·9, 75·1)	9176<br>(8274, 10182)	62·9<br>(56·8, 70)	-6·9<br>(-18·7, 7·7)
Slovakia	5974<br>(5611, 6361)	101·8<br>(95·7, 108·7)	7616<br>(6732, 8705)	86·8<br>(76·7, 99·6)	-14·8<br>(-26·2, -1·5)
Slovenia	2566<br>(2423, 2709)	101<br>(95·6, 106·6)	2351<br>(2101, 2611)	62<br>(55·4, 68·9)	-38·7<br>(-46·3, -31·2)
**Central Asia**	**153270<br>(149652, 156784)**	**306·6<br>(299·2, 313·7)**	**105852<br>(100402, 111524)**	**135·2<br>(128·7, 142)**	**-55·9<br>(-58·1, -53·3)**
Armenia	1567<br>(1449, 1688)	53·5<br>(49·8, 57·4)	1494<br>(1370, 1628)	35·7<br>(32·9, 38·7)	-33·3<br>(-40·4, -25·5)
Azerbaijan	11311<br>(10663, 12027)	205·6<br>(194·4, 217·9)	18940<br>(16410, 22269)	191·6<br>(166·5, 225·1)	-6·8<br>(-20·3, 10·6)
Georgia	2716<br>(2509, 2937)	42<br>(38·9, 45·2)	3631<br>(3305, 3931)	65·9<br>(60·2, 71·3)	56·9<br>(38·9, 75·5)
Kazakhstan	57555<br>(55547, 59643)	424·6<br>(410·5, 439·2)	25754<br>(23733, 28025)	147·2<br>(136·3, 159·5)	-65·3<br>(-68·2, -62·5)
Kyrgyzstan	5955<br>(5560, 6396)	186·6<br>(175·1, 199·7)	3431<br>(3183, 3702)	77·4<br>(71·8, 83·3)	-58·5<br>(-62·1, -54·4)
Mongolia	5612<br>(4727, 6593)	536·6<br>(451·9, 629·2)	8055<br>(7115, 9035)	386·1<br>(342·6, 434)	-28·1<br>(-39·9, -13·1)
Tajikistan	7403<br>(7008, 7841)	246·4<br>(232·7, 260·6)	7327<br>(6446, 8321)	125·5<br>(110·4, 142·3)	-49<br>(-55·9, -41·1)
Turkmenistan	16101<br>(15517, 16645)	772·3<br>(745, 798·5)	8982<br>(8120, 9882)	216·7<br>(195·6, 237·9)	-71·9<br>(-74·7, -69)
Uzbekistan	45052<br>(43405, 46733)	369·2<br>(355·8, 382·9)	28236<br>(24845, 31855)	115·2<br>(101·8, 129·2)	-68·8<br>(-72·4, -64·8)
**Central Latin America**	**41668<br>(40962, 42471)**	**46·1<br>(45·3, 47)**	**73603<br>(70141, 76826)**	**31·1<br>(29·6, 32·4)**	**-32·6<br>(-35·9, -29·4)**
Colombia	14079<br>(13569, 14608)	75·4<br>(72·6, 78·2)	17363<br>(15392, 19623)	32·2<br>(28·6, 36·4)	-57·3<br>(-62·3, -51·8)
Costa Rica	1059<br>(1003, 1115)	57·9<br>(54·8, 61)	1846<br>(1674, 2028)	37·2<br>(33·7, 40·9)	-35·7<br>(-42·8, -28·4)
El Salvador	917<br>(848, 990)	28·8<br>(26·6, 31·1)	1819<br>(1538, 2168)	32<br>(27, 38·1)	11·2<br>(-7·7, 34·5)
Guatemala	1277<br>(1209, 1357)	30·8<br>(29·2, 32·6)	4080<br>(3632, 4566)	35·3<br>(31·5, 39·6)	14·8<br>(0·4, 31·3)
Honduras	680<br>(584, 779)	29·1<br>(25·1, 33·3)	2019<br>(1579, 2561)	32·9<br>(25·7, 41·8)	13·1<br>(-13·5, 50·6)
Mexico	17428<br>(17019, 17852)	37·1<br>(36·2, 38)	32953<br>(31838, 33991)	27·9<br>(27, 28·8)	-24·8<br>(-27·7, -22·1)
Nicaragua	409<br>(369, 457)	23·7<br>(21·3, 26·5)	753<br>(653, 878)	16·1<br>(14, 18·7)	-32·2<br>(-43·6, -18·6)
Panama	526<br>(490, 563)	33·5<br>(31·2, 36)	1226<br>(1108, 1355)	30·9<br>(27·9, 34·2)	-7·8<br>(-19·3, 4·8)
Venezuela	5293<br>(5032, 5564)	51·5<br>(49, 54·1)	11545<br>(9712, 13431)	39·7<br>(33·3, 46·4)	-22·9<br>(-35·5, -9)
**Andean Latin America**	**9977<br>(9412, 10611)**	**45<br>(42·4, 47·8)**	**15643<br>(13998, 17245)**	**28·7<br>(25·7, 31·7)**	**-36·1<br>(-43, -28·7)**
Bolivia	2476<br>(2121, 2848)	70·8<br>(60·8, 81·5)	4331<br>(3407, 5454)	49·2<br>(38·7, 61·8)	-30·5<br>(-46·9, -11·1)
Ecuador	2770<br>(2637, 2903)	48·6<br>(46·1, 51)	4050<br>(3604, 4560)	27·2<br>(24·2, 30·7)	-43·9<br>(-50·7, -36·1)
Peru	4731<br>(4279, 5208)	36·3<br>(32·9, 40)	7262<br>(6106, 8492)	23·6<br>(19·9, 27·6)	-34·8<br>(-46, -20·7)
**Caribbean**	**22709<br>(21586, 24066)**	**84·2<br>(80, 89·1)**	**41093<br>(37610, 45294)**	**80·1<br>(73·3, 88·2)**	**-4·8<br>(-12·6, 3·7)**
Antigua and Barbuda	38<br>(35, 41)	75·2<br>(69·1, 81·6)	63<br>(57, 69)	60·2<br>(54·8, 66)	-20<br>(-28·6, -8·4)
The Bahamas	256<br>(228, 286)	151·1<br>(134·4, 168·8)	487<br>(426, 555)	118·4<br>(104·1, 134·5)	-21·7<br>(-34·2, -5·7)
Barbados	338<br>(315, 364)	124·1<br>(115·2, 133·4)	423<br>(378, 474)	90·5<br>(80·7, 101·5)	-27·1<br>(-35·8, -15·7)

Belize	40<br>(37, 44)	41·8<br>(38, 46)	157<br>(142, 173)	54·7<br>(49·8, 60·2)	30·9<br>(14·6, 51·8)
Bermuda	98<br>(90, 107)	150·4<br>(137·7, 163·4)	109<br>(97, 121)	90·3<br>(81·1, 100·4)	-40<br>(-47·5, -31·2)
Cuba	8361<br>(8026, 8733)	79·9<br>(76·6, 83·5)	21010<br>(18117, 24130)	114·8<br>(99·1, 131·9)	43·7<br>(23·4, 66)
Dominica	63<br>(59, 68)	92·3<br>(86·2, 99·2)	87<br>(78, 96)	97·5<br>(87·4, 107·5)	5·6<br>(-7·8, 19·1)
Dominican Republic	1558<br>(1397, 1730)	37<br>(33·1, 41·1)	4307<br>(3612, 5067)	45·5<br>(38·1, 53·5)	23<br>(-0·6, 49·8)
Grenada	101<br>(94, 109)	151·8<br>(140·3, 164·2)	194<br>(175, 214)	141<br>(126·7, 156·5)	-7·1<br>(-18·4, 4·9)
Guyana	185<br>(172, 199)	43·9<br>(40·9, 47·2)	279<br>(243, 320)	41·3<br>(36·1, 47·4)	-6·1<br>(-19·2, 8·6)
Haiti	4026<br>(3033, 5212)	111<br>(84·4, 143·3)	6112<br>(4500, 8409)	83·2<br>(61·1, 114·9)	-25·1<br>(-42·3, -0·9)
Jamaica	1165<br>(1077, 1264)	65·3<br>(60·3, 70·9)	2061<br>(1706, 2435)	71·8<br>(59·4, 84·9)	10·1<br>(-9·8, 32·9)
Puerto Rico	4898<br>(4680, 5153)	132·4<br>(126·4, 139·3)	3058<br>(2747, 3391)	48·1<br>(43·3, 53·3)	-63·7<br>(-67·5, -59·2)
Saint Lucia	103<br>(96, 111)	114·7<br>(106·3, 122·9)	196<br>(177, 218)	91·3<br>(82·3, 101·3)	-20·3<br>(-29·8, -9·7)
Saint Vincent and the Grenadines	36<br>(33, 40)	50·1<br>(45·8, 55·1)	81<br>(72, 91)	59<br>(52·7, 65·9)	17·7<br>(0·2, 37·3)
Suriname	87<br>(79, 96)	31·7<br>(28·8, 34·6)	197<br>(170, 228)	32·1<br>(27·9, 36·9)	1·4<br>(-15·3, 19·7)
Trinidad and Tobago	501<br>(463, 539)	56·1<br>(51·9, 60·4)	625<br>(495, 777)	34·2<br>(27·2, 42·4)	-39<br>(-51·7, -23·1)
Virgin Islands	76<br>(68, 86)	81·3<br>(72·4, 91·5)	171<br>(136, 199)	92·8<br>(74·2, 107·5)	14·2<br>(-9·4, 37·2)
**Tropical Latin America**	**142864<br>(140031, 146085)**	**141·3<br>(138·5, 144·3)**	**280946<br>(273964, 288173)**	**116·1<br>(113·3, 119)**	**-17·8<br>(-20·2, -15·3)**
Brazil	141236<br>(138356, 144442)	142·9<br>(140·1, 146·1)	276669<br>(269727, 283809)	117·1<br>(114·2, 120·1)	-18·1<br>(-20·6, -15·5)
Paraguay	1628<br>(1450, 1793)	71<br>(63·4, 78·1)	4277<br>(3435, 5289)	78·8<br>(63·7, 97·1)	11<br>(-12·2, 40)
**East Asia**	**4207134<br>(4011555, 4449226)**	**436·1<br>(416, 460·7)**	**4665986<br>(4447417, 4890303)**	**220·9<br>(210·7, 231·6)**	**-49·3<br>(-53·3, -45·7)**
China	4078281<br>(3890143, 4314430)	446·4<br>(425·7, 471·7)	4464980<br>(4247816, 4690846)	222·6<br>(211·9, 233·6)	-50·1<br>(-54·1, -46·4)
North Korea	38187<br>(30127, 47085)	211<br>(168, 257·5)	64891<br>(48202, 81054)	199·5<br>(149·3, 249·5)	-5·4<br>(-28·2, 22)
Taiwan (Province of China)	20574<br>(19908, 21302)	118<br>(114·3, 122)	60949<br>(55938, 66379)	162<br>(148·7, 176)	37·3<br>(25, 49·6)
**Southeast Asia**	**215284<br>(192785, 235968)**	**74·7<br>(66·9, 81·6)**	**380534<br>(355815, 408779)**	**59·6<br>(55·9, 63·9)**	**-20·2<br>(-28·5, -8·5)**
Cambodia	5891<br>(4936, 6865)	113·8<br>(95·6, 132·5)	7650<br>(6265, 9485)	62·1<br>(51·1, 76·2)	-45·5<br>(-56·9, -31·3)
Indonesia	25698<br>(23178, 29296)	22·6<br>(20·4, 25·8)	45586<br>(42349, 48978)	19·4<br>(18, 20·9)	-14<br>(-26·7, -1·8)
Laos	2823<br>(2278, 3377)	119<br>(95·7, 141·9)	2871<br>(2159, 3650)	60·9<br>(46·2, 77·4)	-48·8<br>(-60·8, -34·5)
Malaysia	6403<br>(5863, 7030)	65·6<br>(60·1, 71·9)	15286<br>(13549, 17422)	57·5<br>(51, 65·5)	-12·4<br>(-25·5, 4·7)
Maldives	98<br>(67, 123)	90<br>(65·1, 111·5)	95<br>(86, 106)	31·3<br>(28·2, 34·9)	-65·2<br>(-72·8, -49·8)
Mauritius	559<br>(523, 597)	70·2<br>(65·6, 74·9)	856<br>(777, 940)	49·6<br>(45·2, 54·3)	-29·3<br>(-36·8, -20·9)
Myanmar	80796<br>(64541, 99218)	311·2<br>(250·5, 378·6)	103323<br>(89139, 120844)	214·2<br>(186, 249·8)	-31·2<br>(-47·5, -11·3)
Philippines	11835<br>(11042, 12659)	34<br>(31·9, 36·3)	23704<br>(20359, 27080)	29·8<br>(25·8, 34)	-12·4<br>(-25·3, 2·3)

Sri Lanka	13412 (12191, 14703)	111·2 (101·1, 122·1)	22780 (18718, 27244)	88·4 (73·1, 105·4)	-20·5 (-35·8, -1·8)
Seychelles	88 (79, 98)	155·1 (138·2, 173·3)	144 (129, 160)	125·2 (112·6, 138·2)	-19·3 (-30·9, -6)
Thailand	28382 (25915, 31082)	70·9 (65, 77·4)	73832 (64408, 84687)	72·2 (63, 82·4)	1·8 (-13·1, 18·8)
East Timor	266 (214, 323)	75·9 (61, 91·8)	414 (309, 541)	49·4 (37·4, 64·5)	-34·9 (-50·7, -16·2)
Vietnam	38747 (31744, 46761)	91·7 (75·2, 110·3)	83493 (68934, 104311)	83·1 (69·1, 102·5)	-9·4 (-31·9, 23·7)
**Oceania**	**2021 (1721, 2374)**	**57·2 (49·2, 66·4)**	**4240 (3548, 5080)**	**54·4 (46·3, 63·6)**	**-4·8 (-18, 9·3)**
American Samoa	7 (6, 8)	26·3 (23·4, 29·4)	14 (12, 16)	29·4 (24·9, 33·9)	11·8 (-9·9, 34)
Federated States of Micronesia	31 (25, 36)	57·8 (48, 68·7)	38 (30, 47)	49·2 (39·7, 59·2)	-15 (-34·5, 8·9)
Fiji	177 (151, 207)	42·6 (36·6, 49·5)	429 (368, 501)	54·5 (47·1, 62·8)	28 (2·9, 59·2)
Guam	39 (34, 43)	44 (39·1, 49·5)	112 (98, 127)	59·6 (52·8, 67·3)	35·6 (14·2, 59·9)
Kiribati	36 (32, 40)	86·2 (77, 95·4)	76 (64, 90)	98·7 (84·7, 116·3)	14·5 (-7, 40)
Marshall Islands	14 (12, 17)	77·9 (66·4, 91·7)	29 (24, 36)	78·4 (64·9, 95·2)	0·6 (-18·1, 22·9)
Northern Mariana Islands	7 (6, 8)	31·4 (26·5, 38·7)	20 (17, 23)	35·1 (30·6, 40·5)	11·8 (-12·1, 38·2)
Papua New Guinea	1379 (1124, 1679)	61 (50·1, 73·8)	2916 (2278, 3683)	53·8 (42·9, 66·7)	-11·8 (-28·3, 7·9)
Samoa	32 (26, 41)	35·2 (29, 45·1)	47 (37, 61)	33·4 (26·7, 43·7)	-5·2 (-24·9, 17·7)
Solomon Islands	99 (79, 121)	59·9 (47·7, 72·4)	193 (154, 236)	52·8 (42·1, 64·4)	-11·9 (-29·6, 8·1)
Tonga	29 (24, 34)	49·7 (41·8, 58·3)	44 (37, 53)	53·8 (45·3, 64·1)	8·4 (-13·2, 39·4)
Vanuatu	39 (28, 51)	51·2 (36·9, 66·4)	89 (66, 119)	49·5 (37·1, 65·1)	-3·2 (-25·2, 26·3)
**North Africa and Middle East**	**128396 (113808, 141600)**	**66·6 (59·5, 73·3)**	**233839 (216220, 254338)**	**51·2 (47·5, 55·5)**	**-23·1 (-29·1, -15·2)**
Afghanistan	18786 (10669, 25240)	246 (140·7, 326·9)	25966 (19576, 32518)	204·5 (157·2, 255·1)	-16·9 (-36·6, 44·4)
Algeria	3342 (2976, 3727)	24·9 (22·2, 27·7)	7396 (6589, 8277)	21·7 (19·3, 24·4)	-12·9 (-25·8, 1)
Bahrain	157 (141, 173)	79·6 (71·6, 87·6)	285 (247, 332)	25·8 (22·7, 29·5)	-67·6 (-72·4, -61·8)
Egypt	12457 (11331, 13719)	38·1 (34·7, 42)	30195 (26397, 34535)	44·2 (38·9, 50·3)	16 (-1·6, 35·9)
Iran	33089 (30631, 36216)	113·2 (105·1, 123·6)	61103 (58577, 64963)	85·3 (81·9, 90·6)	-24·6 (-30·6, -18·1)
Iraq	3214 (2694, 3776)	37·3 (31·5, 43·5)	4278 (3856, 4710)	16 (14·5, 17·6)	-57 (-64·9, -46·8)
Jordan	490 (421, 561)	30·1 (25·8, 34·5)	1445 (1226, 1684)	22·1 (18·9, 25·8)	-26·4 (-40·7, -6·7)
Kuwait	272 (253, 294)	34 (31·6, 36·8)	591 (528, 662)	20·8 (18·6, 23·3)	-38·9 (-46·5, -30·5)
Lebanon	788 (645, 922)	32·7 (26·8, 38·2)	1582 (1381, 1799)	25·6 (22·4, 29·1)	-21·8 (-37·4, 2·3)
Libya	679 (563, 819)	32·4 (27, 39)	2070 (1652, 2566)	40·1 (32·3, 49·2)	23·6 (-7·3, 64·4)
Morocco	5005 (4363, 5696)	32·4 (28·1, 36·8)	9796 (8071, 11594)	29·4 (24·3, 34·7)	-9·2 (-28, 14·8)
Palestine	300 (248, 358)	31 (25·5, 36·8)	598 (541, 664)	22·1 (19·9, 24·5)	-28·6 (-42·5, -10·5)

Oman	493<br>(391, 611)	63·9<br>(51·2, 79)	1230<br>(979, 1491)	53·1<br>(42·9, 63·4)	-17<br>(-37·8, 8·5)
Qatar	134<br>(114, 157)	100·4<br>(86, 116·3)	445<br>(360, 542)	42<br>(34·5, 50·5)	-58·2<br>(-67·7, -46·2)
Saudi Arabia	3226<br>(2634, 3973)	48·3<br>(39·5, 59·4)	8044<br>(6789, 9699)	42·2<br>(36·6, 49·5)	-12·7<br>(-31·1, 12·4)
Sudan	12331<br>(8928, 16600)	119·2<br>(86·8, 163·3)	20473<br>(14297, 27764)	104<br>(72·6, 140·3)	-12·7<br>(-32·5, 12)
Syria	1399<br>(1211, 1641)	23·8<br>(20·7, 27·8)	3121<br>(2514, 3835)	22·4<br>(18·1, 27·5)	-5·8<br>(-28·5, 23·4)
Tunisia	948<br>(854, 1052)	17·8<br>(16·1, 19·7)	2189<br>(1728, 2722)	17·7<br>(14, 21·9)	-0·8<br>(-23·6, 25·6)
Turkey	23592<br>(21110, 26339)	58·3<br>(52·3, 65·2)	28775<br>(26069, 31873)	32·1<br>(29·1, 35·6)	-44·9<br>(-52·4, -36·4)
United Arab Emirates	874<br>(614, 1176)	146·3<br>(102, 199·9)	10864<br>(7877, 14807)	202<br>(149·3, 278·2)	38·1<br>(2·7, 92·8)
Yemen	6738<br>(4360, 9498)	117·9<br>(77·4, 166·4)	13175<br>(9564, 17628)	92·7<br>(68·4, 123·7)	-21·4<br>(-44·6, 19·1)
**South Asia**	**771588<br>(729335, 814205)**	**113·4<br>(106·8, 119·7)**	**1424910<br>(1347200, 1507781)**	**97·8<br>(92·5, 103·5)**	**-13·7<br>(-19·7, -6·2)**
Bangladesh	59861<br>(52001, 70428)	109·6<br>(96, 128·1)	75777<br>(64674, 88173)	58·6<br>(50·3, 67·6)	-46·6<br>(-57·9, -33·7)
Bhutan	557<br>(459, 686)	191·7<br>(158·1, 235·6)	681<br>(542, 855)	103·9<br>(82·8, 130·4)	-45·8<br>(-60·1, -26·9)
India	582282<br>(548223, 614467)	105<br>(98·7, 110·8)	1074440<br>(1014268, 1135388)	91·5<br>(86·2, 96·7)	-12·9<br>(-18·4, -5·9)
Nepal	17725<br>(14877, 21313)	160·1<br>(135·3, 192·8)	27772<br>(22568, 33243)	120·8<br>(98·3, 144·1)	-24·5<br>(-40·2, -7·1)
Pakistan	111163<br>(97207, 128080)	180·9<br>(158·1, 207·4)	246239<br>(201579, 296353)	194·6<br>(159·8, 233·7)	7·6<br>(-13·9, 34·7)
**Southern sub-Saharan Africa**	**98588<br>(91197, 107083)**	**319·7<br>(294·9, 347·1)**	**142739<br>(135250, 150139)**	**242·4<br>(229·9, 254·7)**	**-24·2<br>(-31, -16·1)**
Botswana	1885<br>(1486, 2370)	293·5<br>(233·4, 366·2)	2498<br>(2089, 3008)	172·8<br>(144·9, 207·9)	-41·1<br>(-53·6, -24·8)
Lesotho	3673<br>(3100, 4287)	342·4<br>(289, 399·7)	4655<br>(3887, 5540)	361·3<br>(301·8, 427·8)	5·5<br>(-15·4, 31·1)
Namibia	744<br>(632, 867)	95<br>(81·1, 110·9)	1108<br>(928, 1321)	73·9<br>(62·3, 87·7)	-22·2<br>(-37, -3·4)
South Africa	76343<br>(70229, 83459)	325·4<br>(298·3, 355·6)	105318<br>(100437, 110817)	226·9<br>(216·6, 238·3)	-30·3<br>(-36·4, -22·8)
Swaziland	1529<br>(1215, 1868)	454<br>(362·3, 552·2)	2490<br>(1907, 3063)	394·6<br>(303·8, 483·9)	-13·1<br>(-33·6, 12·8)
Zimbabwe	14415<br>(12373, 16660)	316<br>(271, 364·1)	26670<br>(22286, 31584)	349<br>(293·6, 410·4)	10·4<br>(-12·6, 40·2)
**Western sub-Saharan Africa**	**67419<br>(58462, 79219)**	**69·9<br>(60·9, 82)**	**178401<br>(154536, 214115)**	**91·9<br>(80, 109·4)**	**31·4<br>(12·3, 55·4)**
Benin	1858<br>(1609, 2142)	87<br>(75·3, 100·3)	7468<br>(5856, 9540)	148·9<br>(117·3, 189)	71·1<br>(33·6, 122·3)
Burkina Faso	6216<br>(4842, 7274)	134·9<br>(106·4, 157·1)	12927<br>(10855, 15191)	139·7<br>(117·9, 164)	3·6<br>(-18·2, 36·6)
Cameroon	5422<br>(4151, 6391)	108·4<br>(83·3, 127·3)	20387<br>(15500, 25499)	162·8<br>(124·8, 201·3)	50·2<br>(14·1, 92·4)
Cape Verde	408<br>(366, 448)	186<br>(166·3, 204·3)	1125<br>(975, 1294)	254·7<br>(221, 292·3)	36·9<br>(13·9, 62·5)
Chad	2086<br>(1734, 2464)	69·3<br>(57·7, 81·9)	7272<br>(5730, 9247)	124·8<br>(98·5, 158·5)	80<br>(41·8, 128·2)
Côte d'Ivoire	1143<br>(968, 1336)	24·6<br>(20·9, 28·7)	3359<br>(2700, 4122)	29·8<br>(24·1, 36·6)	21·3<br>(-4·9, 54·1)
The Gambia	186<br>(150, 228)	46·4<br>(38·1, 56·6)	510<br>(421, 616)	51·6<br>(43·1, 61·9)	11·1<br>(-12·4, 42·8)
Ghana	5628<br>(4717, 6764)	79·8<br>(67, 95·4)	14896<br>(12617, 17348)	89·4<br>(76·5, 103·2)	11·9<br>(-12·4, 41·7)

Guinea	1535<br>(1353, 1740)	42·8<br>(37·7, 48·4)	3267<br>(2665, 3967)	56·8<br>(46·2, 68·8)	32·7<br>(6·3, 66·3)
Guinea-Bissau	600<br>(495, 715)	132·4<br>(109·9, 156·8)	1560<br>(1239, 1978)	198·1<br>(158·3, 249·6)	49·7<br>(17·5, 97·9)
Liberia	1105<br>(928, 1299)	91·8<br>(77·1, 107·5)	2854<br>(2264, 3569)	135<br>(107·8, 167·6)	47·1<br>(15·8, 89·7)
Mali	2946<br>(2569, 3365)	64·5<br>(56·5, 73·4)	5194<br>(4278, 6358)	57·1<br>(47·1, 69·6)	-11·5<br>(-27·8, 10·3)
Mauritania	1115<br>(903, 1378)	102·9<br>(83·6, 127·4)	2331<br>(1780, 2975)	112·4<br>(85·9, 142·6)	9·3<br>(-14·2, 37·6)
Niger	2511<br>(1844, 3077)	77·1<br>(57·2, 94)	8083<br>(6218, 10102)	100·2<br>(77·9, 124)	29·9<br>(2·3, 64·3)
Nigeria	29048<br>(21441, 39700)	60·3<br>(45, 81·8)	69446<br>(49093, 100923)	78·2<br>(56·2, 112·3)	29·7<br>(-8, 85·4)
São Tomé and Príncipe	39<br>(31, 47)	55·6<br>(44·5, 68)	109<br>(80, 142)	99·2<br>(73·5, 127·9)	78·4<br>(28·9, 141·4)
Senegal	2974<br>(2489, 3669)	84·4<br>(70·6, 104·1)	8809<br>(7160, 11308)	115·4<br>(94, 147·9)	36·7<br>(9·6, 65·7)
Sierra Leone	1530<br>(1129, 1836)	74·3<br>(54·8, 89·1)	4259<br>(3407, 5248)	116·3<br>(93·5, 143·1)	56·5<br>(19·7, 108·4)
Togo	1070<br>(884, 1272)	75·7<br>(63·1, 89·5)	4543<br>(3512, 5845)	116·1<br>(91·5, 148)	53·3<br>(19·9, 92·3)
**Eastern sub-Saharan Africa**	**220675<br>(196648, 245176)**	**262·4<br>(237, 289·6)**	**320631<br>(294337, 350301)**	**187·5<br>(172·4, 205·4)**	**-28·6<br>(-36·9, -19·4)**
Burundi	11911<br>(9601, 15357)	481<br>(391·4, 616·3)	9997<br>(8022, 12425)	215·9<br>(175, 265·9)	-55·1<br>(-65·4, -42·4)
Comoros	889<br>(702, 1158)	388·8<br>(307·8, 506·2)	925<br>(760, 1117)	192·9<br>(158·4, 230·8)	-50·4<br>(-61·9, -35·3)
Djibouti	579<br>(391, 910)	320·1<br>(220·5, 503·7)	1238<br>(833, 1807)	195·9<br>(134·4, 282·4)	-38·8<br>(-60·4, -7·2)
Eritrea	5600<br>(4132, 7265)	487·5<br>(380·1, 620·2)	6468<br>(5248, 7845)	245·5<br>(200, 293·9)	-49·6<br>(-61·7, -33·9)
Ethiopia	49433<br>(40550, 59635)	227·7<br>(190·1, 271·8)	44776<br>(40252, 50216)	108·4<br>(97·1, 121·7)	-52·4<br>(-62·7, -39·8)
Kenya	19482<br>(15212, 25805)	213·1<br>(167·2, 282·4)	43672<br>(36040, 55567)	188·7<br>(155·2, 241·9)	-11·4<br>(-19·9, -2·7)
Madagascar	17015<br>(14294, 20494)	292·2<br>(245·7, 350·8)	21639<br>(17223, 26595)	181·4<br>(146, 221·3)	-37·9<br>(-51·6, -22)
Malawi	25604<br>(14591, 32058)	556·1<br>(334·4, 688·6)	47941<br>(39938, 55773)	598<br>(500·4, 691·8)	7·5<br>(-19, 80·7)
Mozambique	11337<br>(9745, 13128)	170·9<br>(147·5, 196·2)	21364<br>(17818, 25797)	179·7<br>(150·3, 215·3)	5·1<br>(-15, 30·8)
Rwanda	12987<br>(8287, 15804)	397·3<br>(252·5, 482·3)	9646<br>(6801, 12132)	156<br>(110·3, 194·8)	-60·7<br>(-68·6, -50)
Somalia	12435<br>(7157, 18422)	408·5<br>(259·7, 579·4)	18156<br>(13392, 23670)	258·9<br>(193·3, 333·6)	-36·6<br>(-57·2, 4·2)
South Sudan	11210<br>(6797, 17029)	423·7<br>(270·1, 633)	11880<br>(8590, 16204)	282·3<br>(206·9, 381·3)	-33·4<br>(-55·4, 4·9)
Tanzania	14226<br>(10636, 17474)	122·5<br>(94·3, 148·9)	23229<br>(19707, 27404)	93·7<br>(79·8, 109·9)	-23·5<br>(-39·4, 1·8)
Uganda	22341<br>(18734, 26467)	308·7<br>(260·6, 365·2)	50757<br>(42538, 60109)	336·1<br>(282·6, 395·6)	8·9<br>(-13·3, 34·7)
Zambia	5516<br>(4413, 6443)	177·3<br>(144·5, 205·7)	8743<br>(7393, 10137)	129·4<br>(110·3, 148·9)	-27<br>(-42, -8·1)
**Central sub-Saharan Africa**	**67942<br>(58543, 78768)**	**260<br>(224·9, 300·3)**	**102057<br>(86079, 119191)**	**178·8<br>(151·7, 208·6)**	**-31·2<br>(-42·9, -19·2)**
Angola	14715<br>(11112, 18398)	317·4<br>(246·6, 393·4)	25045<br>(20376, 29777)	206·5<br>(170·3, 244·4)	-34·9<br>(-50·9, -11·6)
Central African Republic	4864<br>(3890, 5742)	356·8<br>(290·4, 420·6)	6392<br>(4871, 8158)	256·1<br>(197·2, 322·7)	-28·2<br>(-43·2, -7·6)
Congo	4511<br>(3758, 5468)	368·8<br>(310·4, 443·8)	6078<br>(4751, 7598)	218·7<br>(175, 270·2)	-40·7<br>(-52·5, -26·1)

DR Congo	41231<br>(33336, 50627)	228·8<br>(185·5, 278·3)	60971<br>(47153, 75964)	160·6<br>(125·7, 200·6)	-29·8<br>(-46·1, -11·7)
Equatorial Guinea	881<br>(682, 1104)	392·7<br>(310·1, 487·1)	1185<br>(822, 1666)	235·2<br>(166·8, 327·7)	-40·1<br>(-57·7, -16·2)
Gabon	1740<br>(1338, 2100)	286·1<br>(220·7, 345·1)	2385<br>(1824, 2925)	210·8<br>(162·3, 255·4)	-26·3<br>(-40·6, -6·9)

Appendix Table 5. Country-level proportion of oesophageal squamous cell carcinoma (OSCC) to all oesophageal cancer (OC) cases, Socio-demographic Index (SDI), access to piped water, indoor air pollution index, and Healthcare Access and Quality (HAQ) Index. Results are sorted by OSCC to OC proportion.

Country or territory	OSCC to OC	SDI	Access to piped water	Indoor air pollution	HAQ Index
Benin	97.4	0.3304089	0.7796332	0.9398518	41.98363
Swaziland	97.2	0.5618348	0.7472622	0.5587243	39.6641
Cameroon	96.7	0.4275668	0.6297141	0.8036034	42.99063
Niger	96.5	0.1792128	0.693911	0.9877465	40.53796
Gabon	96	0.5990072	0.8983477	0.1786359	49.86312
Mali	95.9	0.2430999	0.6608586	0.9927573	44.31631
Ethiopia	95.8	0.2837085	0.6444851	0.971646	42.18656
Guinea	95.8	0.293301	0.6895447	0.992389	37.95065
Myanmar	95.8	0.5471697	0.7521398	0.8987807	46.04166
Senegal	95.7	0.3356177	0.7860705	0.7666046	43.31968
Burkina Faso	95.6	0.2436228	0.7681949	0.9427786	42.88351
Eritrea	95.5	0.3593767	0.6274313	0.5782523	37.5352
Ghana	95.5	0.4660732	0.7692041	0.7690164	48.30972
Somalia	95.5	0.2506504	0.3849675	0.9826643	33.9607
Angola	95.4	0.3883473	0.4913355	0.5723949	38.60574
Burundi	95.4	0.252218	0.7412183	0.9879677	40.88814
Malawi	95.4	0.3196185	0.8217424	0.9812899	45.68607
Kenya	95.3	0.4892204	0.6503803	0.8369064	47.919
Madagascar	95.3	0.3440305	0.4014947	0.9962132	42.2817
Mozambique	95.3	0.2678759	0.4586158	0.9636069	41.38808
Rwanda	95.3	0.3539438	0.7308414	0.994279	47.5149
Zambia	95.2	0.4284829	0.6199216	0.8764653	38.90112
Central African Republic	95.1	0.3217212	0.4883434	0.992653	28.89511
Chad	95.1	0.2736233	0.5406239	0.9646205	36.91996
Nigeria	95.1	0.4434152	0.5474162	0.7552633	50.06386
South Sudan	95.1	0.169572	0.6391081	0.9916456	39.23368
Tanzania	95.1	0.3924812	0.5688567	0.9772893	49.20856
Uganda	95.1	0.337937	0.7467108	0.9867676	42.09132
Mongolia	94.9	0.6670125	0.6764177	0.7859513	56.42357
Lesotho	94.8	0.5212262	0.8217002	0.6309745	35.47194
Comoros	94.4	0.4187506	0.8964573	0.8052982	47.73715
Namibia	94.4	0.5971622	0.8950112	0.592881	52.2355
Botswana	94.3	0.6754337	0.9601774	0.4042182	50.02428
Togo	94.2	0.373095	0.500731	0.949856	42.67131
Mauritius	94	0.7387585	0.9858173	0.0428925	63.97056
Sierra Leone	93.8	0.3217548	0.5785236	0.9905465	39.49081
Thailand	93.3	0.7087615	0.5789928	0.2042466	69.65243
Estonia	92.3	0.8765746	0.9836423	0.1059044	79.51588
Liberia	92.3	0.2975346	0.7128679	0.9918571	44.07752

Papua New Guinea	92	0.3936445	0.4669533	0.8296739	36.99497
Slovakia	89.3	0.8683832	0.9637777	0.0370221	76.32784
Latvia	89	0.8501076	0.9838138	0.0829824	75.93285
Singapore	88.7	0.8727415	0.9843521	0.001393	85.16716
Cambodia	87.8	0.4699593	0.5026168	0.8607864	47.55624
Guyana	87.5	0.6208591	0.6512252	0.1022214	48.00732
Poland	87.2	0.8552011	0.9624795	0.0809213	77.51173
Lithuania	86.7	0.8618109	0.9679642	0.0304079	75.07384
Indonesia	86.4	0.6432723	0.6838619	0.4665855	47.89911
China	86.3	0.698382	0.9131855	0.462104	71.97395
Croatia	86.3	0.8429422	0.986375	0.1245127	79.68633
Belarus	86.1	0.8098246	0.9753374	0.0122462	72.75752
Pakistan	85.6	0.4782369	0.9216352	0.6812232	42.10178
Chile	85.2	0.782218	0.9443579	0.0663029	74.66572
Serbia	85.1	0.7614746	0.8851467	0.287456	74.17399
Slovenia	85	0.8714591	0.9955854	0.0720408	85.78991
Sri Lanka	85	0.6656639	0.8730677	0.6901498	70.55164
Turkmenistan	83.4	0.7258834	0.9039219	0.0133138	55.94375
Belize	83.3	0.6079586	0.7350473	0.1599554	56.99096
Fiji	83.3	0.7205338	0.9458302	0.3085322	45.64901
Uzbekistan	83.3	0.6707062	0.9093039	0.1939895	60.7042
India	82.9	0.5430225	0.8903347	0.5929375	42.59068
Kyrgyzstan	82	0.6140479	0.8899494	0.3045161	58.98535
Kazakhstan	81.2	0.7396165	0.9474311	0.1340845	59.98231
Albania	80.9	0.710576	0.8464829	0.2981147	76.56293
Afghanistan	80.7	0.2548034	0.5715241	0.8136431	30.64038
Tajikistan	80.6	0.563803	0.7245759	0.3493501	56.7099
Ukraine	80.6	0.7854332	0.9399671	0.0454767	71.97182
Bangladesh	80.1	0.4759489	0.9811701	0.8964234	50.05209
Bhutan	80	0.5524619	0.9484282	0.4482564	51.0114
Hungary	80	0.8383694	0.9951285	0.1326214	77.2195
Romania	80	0.8222468	0.8043945	0.1768524	72.51046
Suriname	80	0.6734836	0.9144762	0.1625742	54.5168
Brazil	79.9	0.6855165	0.9844739	0.080044	63.62204
Trinidad and Tobago	78.6	0.7599835	0.9342995	0.006505	61.27617
Japan	78.4	0.876547	0.9856223	0.0009266	88.50639
Nicaragua	78.1	0.5620469	0.8729433	0.5179757	62.91613
Haiti	77.7	0.4127922	0.4884083	0.9370795	36.90585
Nepal	77.7	0.4171257	0.8930547	0.8091615	48.94768
Cuba	77.5	0.7572473	0.931891	0.0296171	72.3793
Paraguay	77.3	0.635765	0.9003635	0.4127544	59.28289
Bolivia	77.2	0.5861735	0.8867787	0.2813707	57.46043
Venezuela	77.2	0.7208425	0.955869	0.0168833	64.94327

Guatemala	77	0.5367455	0.9348995	0.4860598	54.04214
Portugal	77	0.7866725	0.9977095	0.024514	82.56662
Dominican Republic	76.9	0.6683926	0.4720352	0.1204211	61.6235
Montenegro	76.9	0.8030106	0.9569953	0.4192286	79.38941
Mexico	76.7	0.728041	0.9473426	0.066959	61.28267
Mexico	76.7	0.712159	0.9513972	0.1444901	62.98865
Honduras	76.6	0.5448053	0.6802787	0.541191	52.876
Panama	76.4	0.7175655	0.9454027	0.1622151	62.72926
Peru	76.3	0.6632558	0.8379584	0.324628	67.29581
Jamaica	75.8	0.6911253	0.9044116	0.119185	63.1513
El Salvador	75.6	0.6169341	0.9096631	0.2106771	62.94157
Uruguay	75.3	0.7294097	0.9616365	0.0167805	71.22957
Maldives	75	0.6509901	0.8761854	0.0762308	74.98264
Greece	74.8	0.8525788	0.9946353	0.0072727	85.34872
Ecuador	72.3	0.6553559	0.8382855	0.0695997	59.80069
Italy	72.2	0.8616537	0.9731147	0.0094357	87.99769
Argentina	71.8	0.7445155	0.9773318	0.0403481	67.26058
Puerto Rico	71.3	0.8682803	0.9678878	0.0027099	74.85159
Turkey	70.9	0.7309635	0.7236533	0.0289763	75.37779
Philippines	70.5	0.6190118	0.6470509	0.6294172	50.63124
Barbados	70	0.7899293	0.9874884	0.0015912	66.13252
Spain	69.9	0.8431252	0.9979746	0.0146491	88.14818
Yemen	69.7	0.3979337	0.4910526	0.3491732	48.76109
Zimbabwe	69	0.4737589	0.7733714	0.6886539	44.06696
Bulgaria	68.9	0.8323442	0.9602885	0.1264881	69.03984
Germany	68.9	0.8796307	0.995062	0.0119275	85.48669
Colombia	68.2	0.6790322	0.9231676	0.2088578	65.65761
Costa Rica	65.1	0.7036828	0.9938776	0.078099	71.65074
Iraq	65	0.4323598	0.8237174	0.0120258	58.54839
Saudi Arabia	65	0.7458847	0.9221632	0.0203691	78.42365
Sudan	64.9	0.4801974	0.6798086	0.6500466	48.42831
Tunisia	64.9	0.6666551	0.8573115	0.0073916	68.5005
Egypt	62.3	0.6225914	0.9696563	0.0048356	59.06907
Malaysia	62.1	0.7422902	0.9788215	0.0150146	64.65762
Lebanon	61.9	0.7966968	0.6335686	0.0001295	78.45633
Czech Republic	61.5	0.8730644	0.9968561	0.0260234	83.04813
United Arab Emirates	60.9	0.7506719	0.8273565	0.0495193	71.54
Azerbaijan	60.6	0.7466661	0.8429279	0.163501	61.82902
Austria	60.5	0.8845051	0.9786069	0.0133661	86.91602
Switzerland	58.9	0.8932123	0.9952022	0.0140723	90.88026
Finland	56.7	0.9001482	0.9955437	0.0073241	88.23843
Morocco	55.6	0.5893786	0.8282948	0.0573885	59.87075
Belgium	54.6	0.8930314	0.9953721	0.0049786	86.94695

Armenia	54.5	0.7347708	0.9558374	0.0229359	65.35338
Oman	54.5	0.675623	0.7605554	0.0725301	73.62875
Georgia	52	0.7314408	0.9586898	0.4526661	61.34325
Cyprus	50	0.8675599	0.9949666	0.0089806	83.90007
Jordan	50	0.6694312	0.7833459	0.0005995	75.79024
Kuwait	50	0.8081329	0.9239886	0.0177137	79.12867
Malta	50	0.8450488	0.9944727	0.0090271	83.53316
Denmark	49.3	0.9222978	0.9959613	0.0041093	84.44193
Sweden	47.5	0.866955	0.9946761	0.0175916	89.31772
Algeria	47	0.6418771	0.8718388	0.0041284	63.00277
Luxembourg	45.8	0.928878	0.9958673	0.0065134	88.09482
Australia	45.6	0.8785403	0.9855105	0.0047648	88.75545
Ireland	45.4	0.8737457	0.9948518	0.0093545	86.77013
Iceland	45	0.9094412	0.9957234	0.006488	92.61529
Norway	44.1	0.9162598	0.9956161	0.0098957	88.90649
Qatar	42.9	0.7693111	0.91709	0.000657	84.70576
Canada	40.9	0.902691	0.993894	0.0019987	86.90737
New Zealand	39.8	0.8556332	0.9844599	0.0060908	85.09032
Israel	39.6	0.7956893	0.9928728	0.0100482	84.61298
Libya	38.9	0.7928386	0.9236626	0.0080853	70.90197
United Kingdom	36.4	0.8528052	0.9943225	0.008295	83.44392
Bahrain	28.6	0.7180979	0.9014279	0.061035	78.42273
